# Altruism by age and social proximity

**DOI:** 10.1371/journal.pone.0180411

**Published:** 2017-08-24

**Authors:** Mark C. Long, Eleanor Krause

**Affiliations:** 1 Evans School of Public Policy and Governance, University of Washington, Seattle, Washington, United States of America; 2 Evans School of Public Policy and Governance, University of Washington, Seattle, Washington, United States of America; Universidad de Alicante, ITALY

## Abstract

This study evaluates the extent to which an individual’s stated altruistic sentiments can be influenced by context–most importantly, by the age and social proximity of the other person and by the nature of what is being sacrificed. We measure willingness to sacrifice own health for another person’s health and willingness to sacrifice own wealth for another person’s wealth. To evaluate these sentiments, two surveys were administered to representative samples of Americans which contained hypothetical scenarios with context randomly assigned; the first survey posed a dictator game question and the second survey was designed to elicit marginal rates of substitution between own and other’s health/wealth. As expected, we find less altruism towards those who are more socially distant (e.g., strangers relative to family). We find individuals are more health altruistic towards young children and more wealth altruistic towards adults, and health altruism tends to be lowest for survey respondents near retirement age. We find no relationship between levels of altruism and the distance between the respondent’s state of birth and state of current residence. These findings improve society’s understanding of situational altruism and kinship and reciprocity as motivations for altruism, and they have practical implications concerning the economic valuation of human lives used to guide public policy-making.

## Introduction

Understanding the extent to which individuals exhibit altruistic behavior under different circumstances and towards individuals of varying levels of social proximity are fundamental inquiries for social science research. Social preferences play an integral role in guiding the provision of public goods, but social preferences differ tremendously in different contexts. Specifically, prosocial actions taken to improve either the health or wealth of others likely depend upon the relationship between the givers and beneficiaries of these actions. This study addresses how much these relationships and circumstances determine the level certain types of altruistic behavior. This paper evaluates whether individuals are more likely to express altruistic sentiments towards others who are more socially proximate; whether that altruism is conditioned on the age of the other person and self; whether there are differences in levels of altruism regarding health or wealth of self and other; and whether individual characteristics, such as the extent to which the individual has moved away from his/her birth state, are associated with levels of altruism.

While theories of kin and reciprocal altruism (discussed in greater detail below) guide our understanding of the motivations for altruism, the relative magnitude of altruistic sentiments by age and social proximity has not been studied extensively. Genetic relatedness certainly promotes greater levels of altruism in the context of basic evolutionary biology, but genetic relatedness is not a prerequisite for entering prosocial cooperative agreements. Reciprocity is also a fundamental motivator for prosocial behavior, but society’s understanding of *how much* prosocial behavior is motivated by other, non-genetic factors is relatively limited. To understand the implications of these variables on an individual’s altruistic sentiments, we created two stated-preference surveys, and administered these surveys to representative samples of Americans. Our methodology enables us to determine how levels of giving, in both the allocation of monetary wealth and of products designed to improve one’s health, differ in different circumstances. We find that four-fifths of the altruism that is explained by our model can be attributed to variation across survey respondents, while the remaining fifth reflects variation in a respondent’s reported altruism related to the situation presented. That is, most of the variation in altruism is *between* individuals rather than situational. As expected, we find less altruism towards those who are more socially distant (e.g., strangers relative to immediate family members). Though not surprising, this result casts important empirical support for the large body of theoretical literature concerning the existence of reciprocal altruism. Perhaps more interestingly, we find that individuals are more health altruistic towards young children and more wealth altruistic towards adults, and health altruism tends to be lower for older survey respondents. We find no relationship between the level of altruism and the distance between the respondent’s state of birth and state of current residence. Social preferences are critical in guiding how policy-makers seek to improve the health and economic wellbeing of the public. Thus, understanding how these social preferences related to supporting others’ health and wealth differ based on the variables included in this study can improve the empirical grounding of public policy.

## Literature and theory

### Theoretical explanations for altruism: Effects of the situation and the nature of the other person

A large body of literature supports the notion that the relationship or social distance between two individuals will influence the magnitude of their altruistic behaviors toward one another. W.D. Hamilton first explored the theory behind “kin” altruism in 1964 –the observation that there may be genetic drivers behind altruistic behaviors [1]. This theory suggests that an individual is more likely to be altruistic toward another individual with stronger genetic ties so as to increase the odds that the individual’s own genes are passed down. “Reciprocal” altruism, by contrast, does not rely on genetic proximity between the giver and receiver of altruism. Reciprocal altruism exists when an individual makes sacrifices for an unrelated other who is likely to act in a reciprocal manner in the future–such a theory explains cooperation among nonrelatives [2]. Robert Axelrod uses game theory to explain biological cooperation between unrelated species and individuals [3]. He found that when participants recognize the opportunity for mutual benefit, they often behave in such a manner to preserve this benefit. Axelrod’s work supports R.L. Trivers’ original insight into why individuals may behave altruistically toward unrelated others–these “acts of kindness” may actually be executed with the hope that the other will eventually return the favor.

Yet, there are moral appeals to disregard familial relations, social proximity, and the likelihood of reciprocity when faced with opportunities to act altruistically. For example, the Parable of the Good Samaritan (Gospel of Luke 10:25–37) presents a story in which a Samaritan provides assistance and compassion to a socially distant traveler who had been robbed and beaten, and who had been ignored by other more socially proximate passersby. An act of a “good Samaritan” has now become a colloquial expression for any act of kindness offered by a stranger. If such pleas to act without regard for situation and social proximity were broadly heeded, then evidence for kin and reciprocal altruism would be less available.

Most of the empirical work that considers social proximity and altruism indicates that relationship does play an important role in determining altruistic sentiments. Madsen et al. show that individuals demonstrate greater levels of altruism towards others who were more closely related, but the relationship between relatedness and altruism is not perfectly linear [4]. A study conducted by Rachlin and Jones found that the closer an individual felt to another person, the more altruistic sentiments would be demonstrated toward that person [5]. However, even at the same social distance, participants were willing to sacrifice significantly more money in order to benefit relatives compared to unrelated individuals, a finding that is consistent with Hamilton’s kin-selection theory. Curry et al. confirmed this finding, showing that altruism was greater toward family members than friends [6]. Participants were more altruistic toward kin even controlling for the effects of emotional closeness, a phenomenon that the authors call the “kinship premium.”

Other empirical work investigates how social relationships beyond kinship affect altruistic sentiments. Hurley and Mentzakis show that survey respondents were more willing to provide charitable donations to improve the health of friends than strangers, and more willing to contribute to provide for medically necessary care for residents of their neighborhood than for residents in a distant city [7]. Curry and Dunbar show that individuals are more likely to demonstrate altruism toward well-connected members of their social networks [8]. If one assumes that a well-connected member of society is more able to offer future social or financial benefits to the giving individual, the theory of reciprocal altruism provides some theoretical insight into these findings.

Finally, there is a strand of literature that finds that altruistic behavior can be affected by priming and activated by evoking norms and moral obligation (see, for example, Schwartz [9]; Macrae and Johnston [10]; Nelson and Norton [11]). Studies by Simpson and Willer similarly suggest that there is significant heterogeneity regarding the motivations and reputational incentives for pro-social and altruistic behavior [12]. This research strongly suggests that individuals are likely to behave differently when faced with different situations. Our findings discussed below affirm that priming matters.

Based on this literature, we test the following null hypotheses:

H1: Social proximity between the potential giver and receiver has no effect on the giver’s altruistic sentiments.
H2: Priming has no effect on altruistic sentiments.

Our goal is not only to test these hypotheses, but as importantly, to compute the extent of deviations from these null hypotheses and the magnitude of the impacts of these (and other attributes) on altruistic sentiments.

### Individual attributes as determinants of altruism

Other studies estimate the variation in altruistic sentiments depending on characteristics of the giving individual. Lowe and Ritchey find that altruism is exhibited more strongly in the adult population [13]. Eckel and Grossman find that women, on average, give away twice as much as men during double-anonymous dictator experiments [14]. However, despite this rich base of literature, there is a lack of empirical evidence considering how the *age of the receiving individual* combined with the social proximity between the giver and recipient affects altruistic sentiments. There is also a lack of evaluation regarding whether there are different relationships of altruism by age and social proximity for different types of donations.

The prior literature also has not directly considered how population mobility affects altruism. Yamada [15] finds neither significant relationship between adults’ altruistic sentiments towards their parents and the likelihood of co-residence with their parents nor distance between their residence and their parents’ residence. Rotton [16] found that type or region of residence of the potential giver (i.e., living in apartments/single-family dwellings/commercial sites and city/suburbs) did not significantly affect altruistic behavior. Angeline and Laferrère [17] find that more constrained parents were “proximity altruists” (i.e., helping their children to stay in co-residence for a longer period), while parents that are more affluent were able to be “active altruists” and help the children move out of the nest at an earlier age.

One might expect that deeper relationships are formed when people stay in small geographical areas. Individuals living in closer proximity to their birthplace may feel more closely connected to their neighbors, having fostered a larger sense of community over time and therefore would exhibit greater levels of altruism if social proximity does play a role in the magnitude of altruistic sentiments. On the other hand, as people travel the world and get exposure to other cultures and peoples, their fear of others may decline and their altruistic sentiments grow. For context, the U.S. Census Bureau reported that the percentage of people who moved residences reached a record low between 2010 and 2011 (11.6 percent) since statistics were first collected on this subject in 1948, and this mobility has steadily declined during this sixty-year period [18]. Of course, radical changes in telecommunications, the advent of the Internet, and the rise of social media have fundamentally changed the ability of people to make and sustain distant relationships.

Based on this literature, we test the following null hypotheses:

H3: The giver’s age has no effect on altruistic sentiments.
H4: The receiver’s age has no effect on altruistic sentiments.
H5: The giver’s gender has no effect on altruistic sentiments.
H6: The distance moved away from the giver’s birth state has no effect on altruistic sentiments.

### Dictator games

Researchers (frequently economists) have often used a method known as a “dictator game” to understand the magnitude of altruistic sentiments. A dictator game provides an individual with an original endowment *q* of money or a specified good. The dictator is free to choose any allocation between 0 and *q* of this good to send to the other person, leaving 1-*q* for him or herself to keep [19]. By changing the relationship of the receiver to the sender, researchers can elicit the variation in altruistic sentiments or behavior across relationship types. In economic terms, “an altruist is willing to reduce his own consumption in order to increase the consumption of others” [20]. If self-interest is the primary motive for individual behavior, and if the utility of the dictator is not affected by the amount given to the other person, then self-interest would yield the dictator keeping the entire endowment.

Forsythe et al. find that most players in a dictator game give away nontrivial proportions of the original wealth endowment provided to them [21]. Falk and Fischbacher summarize the findings of various dictator experiments as follows: (1) Dictators rarely offer more than half of the original endowment to the other person; (2) Dictators offer between zero and half of the original endowment to the other person about 80 percent of the time; and (3) the rest of the time (20 percent), dictators offer nothing to the other person [22]. In a meta study summarizing the results of 131 papers containing 616 different treatments, Engel finds that the dictators on average gave away 28% of the money to the other participant [23]. One of the two surveys conducted for this study is a stated-preference dictator game. As shown below, we find an average level of wealth altruism quite similar to that found by Engel (27.7%), but with significant variation based on the characteristics of the giver and receiver.

### Marginal rate of substitution: Economic theory

The second survey that we construct (discussed below) is designed to elicit the marginal rate of substitution (MRS) for both health and wealth altruism. If multiple units of a single product are allocated between a utility-maximizing individual and another person, economic theory would indicate the following: As the individual accumulates more and more of a product, the marginal utility of having one additional unit of that product for himself should diminish with each unit. Similarly, as the other person has less and less of that product, the marginal utility to the individual of giving the product away to the other person should increase. The point at which an individual switches from receiving to giving away the good is the MRS, or the marginal willingness of person *i* to exchange a slight decrease in his own health or wealth for a slight increase in the other person’s health or wealth.

### The role of altruism in regulatory policies

To estimate whether the benefits of a policy affecting the longevity of citizens’ lives outweigh the policy’s costs, benefit-cost analysts use a concept called the “value of a statistical life” (VSL), computed using estimates of how much people are willing to pay for a small reduction in their risk of death. While altruistic sentiments are typically ignored in these analyses, capturing the full value of life to all affected parties (rather than to a single individual) has enormous implications on public policies. If *safety* altruism (i.e., “health” altruism) contributes to how much society is willing to pay to reduce an individual’s chance of death, the VSLs used by regulatory agencies might be significant understatements. Jones-Lee suggests that if people are willing to pay for other people’s safety, the VSL should be adjusted to account for this altruism [24].

Andersson and Lindberg find that people are not purely selfish when it comes to the safety of others, and it is most likely that altruistic sentiments take the form of safety paternalism [25]. This notion is supported by Jacobsson et al., finding that people are more likely to donate a health-benefiting product than the equivalent monetary donation, even if the receiving individual would prefer cash [26]. Brady confirms that people are willing to pay to reduce others’ health risks [27]. These studies largely confirm the statement made by Kenneth J. Arrow in 1963, “The taste for improving the health of others appears to be stronger than for improving other aspects of their welfare” [28]. The true value of preventing an individual’s death (and the resulting VSL) should include the willingness to pay of others’ to prevent this death (see also Johannesson et al. [29]). Jones-Lee shows that the altruism-adjusted VSL will increase with more safety-focused altruism and decrease with more wealth-focused altruism [30].

Given the importance of this distinction in the regulatory and economics literature regarding health and wealth altruism, it is critical to understand how the characteristics of the giver and receiver may affect the relative magnitude of these types of altruism differently.

## Methods and data

There are no quantifiable real-world contexts in which an individual person “reveals” her valuation of own health or safety relative to another’s health or safety. Thus, it is necessary to use survey-based stated preferences to estimate levels of health altruism. While wealth altruism is exhibited in the real world through acts such as charitable donations, so as to compare health and wealth altruism and how they are affected by the age and social proximity of the receiver, we construct parallel stated-preference scenarios. Survey 1 presents a typical dictator game, while Survey 2 elicits similar preferences using a standard dichotomous choice framing technique. The graphics used in these survey questions as well as some of the preliminary questions designed to explain probability and test understanding were designed to mimic the contingent valuation work in Krupnick et al. [31]. The supporting information files (S1 File and S2 File) includes the full surveys. In both surveys, contextual factors, including the age and social proximity of the other person, are randomly assigned.

The two surveys were fielded by Knowledge Networks (KN) to members of their KnowledgePanel®, which is an online panel based on random sample of residential addresses that covers approximately 97% of U.S. households and with weights provided to make the sample representative of the full U.S. population. “Non-Internet households that are selected in the sample are provided a web-enabled computer and free Internet service so they can also participate as online panel members” [32]. Weights are used to produce descriptive statistics and in regression analysis.

Survey 1 was fielded in winter 2013 to 528 member of the panel. We drop from the analysis 32 respondents who answered fewer than 10 of the 20 questions measuring health and wealth altruism, leaving an analysis sample with 496 respondents. Survey 2 was fielded in the summer of 2015 by KN to 1,144 panel. With around 50,000 adults on the KnowledgePanel and a good degree of turnover, there is little chance of overlap between the respondents on either survey. (Only 17 of the 1,144 respondents to the second survey were participants in the first). These surveys, respectively took 23 and 27 minutes to complete for the median respondent. The University of Washington’s Human Subjects Division (IRB) specifically approved this study.

### Survey 1: Dictator game

The key questions in Survey 1 revolve around a hypothetical scenario comparable to a dictator game. In the first set of questions, which measure health altruism, the respondent was told that a company is prepared to distribute 10 medical products or safety inventions, where each product/invention would lower the recipient’s chance of death during the next ten years by one chance in 1,000. The respondent was told that the company had asked the respondent to allocate the 10 medical products and safety inventions between himself and one other person, with the age and social relation of the other person given in the question. The respondent was provided 10 variants of this question, each of which varied the age and social relation of the other person.

A parallel scenario was presented to measure wealth altruism: the respondent was told that the company was prepared to distribute 10 scratch-off tickets, where each ticket had one chance in 1,000 of winning $25,000 from the company. The respondent was again asked to allocate the 10 tickets between herself and one other person, with the age and social relation of the other person given in the question. Again, 10 variants of this question were asked.

The reason that we make the “wealth” question about giving a scratch-off ticket with the prospect of receiving money, rather than a direct payment of cash, is that we wanted the “wealth” and “health” questions to be symmetrical. In the case of health, one cannot directly *give health*; rather, one can only give things that increase the probability of survival. Thus, correspondingly, we frame the wealth questions to be about increasing the probability of an improvement in wealth. Note that asking people to distribute probabilities rather than payoffs might yield different levels of altruism. For example, Krawczyk and Le Lec [33] find smaller contributions in a dictator game when respondents distribute probabilities of payoffs rather than actual payoffs. See also Brock, Lange, and Ozbay [34] and Krawczyk and Le Lec [35].

To investigate whether framing influenced responses, half of the survey respondents were asked the set of 10 wealth altruism questions before the set of 10 health altruism questions. Additionally, whether the “other person” was shown on the left or right of the screen was randomized.

There are 70 possible combinations of age and social relation of the other person, and each survey respondent was asked about only 10 of these combinations in each set of health and wealth altruism questions. These combinations include ten age groups and seven social proximity groups (such as close friends or acquaintances). The set of questions each respondent answered varied across the sample. Survey respondents were only asked about relationships that the respondent actually had. For example, the respondent was only given a question about safety altruism towards an immediate family member age 10–17 if the respondent reported having such a relationship in earlier questions.

One might be concerned that we will observe lower stated altruism when asked about acquaintances, for example, in a particular age group than when asked about immediate family members of the same age range due to the fact that a reference to an immediate family members in a particular age group may conjure in the respondent’s mind a *specific* individual whereas an acquaintance of the same age conjures a less distinct person. We have some ability to test this hypothesis. Our data allow us to test whether a respondent who has more than one immediate family member in a particular age group, say 50–59, is less altruistic than respondents who have only one immediate family member in that age group. We do not find consistent evidence to support this hypothesis. Across the 10 age groups that are specified, the mean differences are +0.1 for health and 0.0 for wealth, with no clear patterns by age of the immediate family member. Our data do not allow us to do the same test for other relationship groups, as we did not ask for detailed inventories of number of persons in each age group for other relationship types.

The data allow us to evaluate the effect of distance between birth state and current state. While it might be reasonable to expect that the effect of moving away from a birth state as a young child on altruistic sentiments will be different from the effect of moving as a young adult for work or college, the data do not allow us to tell when, why, or how many times the person moved. Also, note that we cannot tell the difference between someone who returned to his or her birth state after moving away from someone who never moved.

Since each respondent contributes up to 10 observations to each regression, we use robust standard errors clustered by respondent in our regression analysis.

### Survey 2: Dichotomous choice

In Survey 1 (explained above), it is theoretically possible that the choice presented to the respondent will result in what is known as a “corner solution” in microeconomics. This occurs when the optimal point on an individual’s budget constraint exists in the corner of the constraint and the graph’s axis, implying a utility-maximizing quantity of zero for one of the goods. In this case, the survey respondent might indicate that they would like to keep all of the product or money for himself and give none to the other person, even if they do in fact care about the other person’s safety or wealth. Survey 2, in contrast, is designed to elicit the respondent’s marginal rate of substitution–i.e., how much would the other person need to receive before the respondent would be willing to give up one unit of health/wealth.

The second survey is largely similar to the first, but the questions are framed in a dichotomous choice method, insisting that the survey respondent choose between two mutually exclusive options. To elicit levels of health altruism, respondents were told to “imagine that a company decided to give out special medical products and/or safety inventions that could lower a person's chance of dying during the next 10 years.” The respondents were asked to “decide if the company should either give you a given number of medical products or safety inventions or give some other person a given number of medical products or safety inventions.” The respondents were told the age range of the other person (for example, 40–49 year old), and their relationship to the respondent (for example, “acquaintance”). As in Survey 1, the respondent was only asked questions about a relationship type that the person reported having. In each question, the respondent was asked to select whether she would like the products/inventions to go to her or to the other person.

For example, the respondent was asked to “select the option that you most prefer” from the following graphic (Table 1):

**Table 1:** “Select the option that you most prefer”.

☐	Option 1:
	The company gives **8** medical products or safety inventions to **you** (and gives nothing to the other person)
☐	Option 2:
	The company gives **8** medical products or safety inventions to a **40–49 year old acquaintance** (and gives nothing to you)

Each respondent was told to remember that “each product/invention increases the recipient’s chance of surviving 10 years by 1 chance in 10,000.” The dichotomous choice presented next would depend on the respondent’s answer to the initial question. If the respondent selected Option 1 (reflecting more regard for the health of herself than the other person) when faced with the above choice, then the bold, underlined numbers were changed to 4 versus 8 and the question was asked again so to assess how far the regard for self over the other person goes. If the respondent continued to select Option 1, he was given progressively less generous scenarios (2 versus 8, 1 versus 8, and finally 1 versus 15). Otherwise, if he switched to Option 2 when faced with 4 versus 8, he was then given a new scenario where the ratio was increased to 6 versus 8. Thus, the relative generosity possible in each round of questioning was dependent upon the respondent’s choice to keep or give the product in the previous question.

Survey 2 asked questions about allocating products that each had 1 chance in 10,000 of impact, whereas Survey 1 asked questions about allocating products that each had 1 chance in 1,000. We made this switch to account for the fact that we would be asking questions where the person could be allocating as many as 15 medical products or safety inventions and baseline risks of death during the next 10 years are less than 15 in 1,000 for some age groups. Thus, by switching to 1 in 10,000 impacts, we could feasibly ask questions about giving medical products/safety inventions that would lower risk of death in the next ten years by 15 in 10,000 chances.

The respondent was asked five sets of questions to elicit health altruism, varying the age and relationship of the other person yielding 5 of the possible 70 MRSs of interest. The original choice offered to the respondent varied in each set of questions to avoid the anchoring bias that could result if each respondent began with an even initial allocation [36]. One-third of each set of questions had the initial scenario with allocations to self and the other person (8 versus 8), one-third started with a less generous initial allocation (12 versus 4), and one-third started with a more generous initial allocation (4 versus 8). Varying this initial allocation provides us the opportunity to evaluate if the priming influences the level of exhibited altruism. The full question progression is shown in the supporting information files (S1 Fig).

The object is to find the point at which the respondent switched from Option 1 to Option 2. This switching point reflects the respondent’s marginal willingness to exchange a slight increase in the probability of his own death for a slight decrease in the probability of the other person’s death (the marginal rate of substitution discussed earlier). For example, if we were to find that the respondent switched from Option 1 to Option 2 between 6 v. 8 and 5 v. 8, then we would conclude that the respondent was willing to decrease his survival probability by more than 5 chances in 10,000 (but less than 6 chances in 10,000) to increase the other person’s survival probability by 8 chances in 10,000. Thus, the MRS for this person lies in the interval between $\frac{5}{8}$ and $\frac{6}{8}$. The series of questions allows us to identify that the MRS lies below $\frac{1}{15}$, above $\frac{15}{1}$, or in one of 16 intervals in between these values. If the person always selected Option 2 (even when offered 15 to self versus 1 to the other person), then she was asked a question to identify whether she rejects the scenario (e.g., she does not want the medical products/safety inventions even if they were given for free). Respondents who rejected the scenario were dropped from the analytical sample. To compute the MRS, we make the following ad hoc assignments: MRS = mid-point for each interval (e.g., assigning $\frac{5.5}{8}$ for the MRS when the MRS lies in the interval between $\frac{5}{8}$ and $\frac{6}{8}$); MRS = $\frac{1}{20}$ for the cases where the MRS lies below $\frac{1}{15}$; and finally, MRS = $\frac{20}{1}$ for the cases where the MRS lies above $\frac{15}{1}$.

The wealth altruism questions were constructed in a parallel fashion, where the respondent was told to “(i)magine that a company decided to give out scratch-off tickets and that each ticket has one chance in 10,000 of winning $25,000 from the company.” Again, the respondent was asked to choose between Options 1 and 2 (i.e., to select whether she would like the scratch-off tickets to go to herself or to the other person). The value at which the respondent switched from Option 1 to Option 2 yields the respondent’s marginal willingness of to forgo her own wealth for a gain in the other person’s wealth (the MRS).

Just as in Survey 1, half of the survey respondents were asked the wealth altruism questions before the health altruism questions and whether the “other person” was listed as Option 1 or Option 2 was randomized. Since each respondent contributes up to 5 observations to each regression, we use robust standard errors clustered by respondent in our regression analysis.

### Analytical methods

To test the statistical significance of the association between age, social proximity, and individual characteristics with levels of health and wealth altruism, we estimate ordered probit regressions as given in Eq 1:

$$Pr(Y_{ij}=k)=Pr(T_{k-1}<(P_{ij}\prime \beta_{1}+A_{j}\prime \beta_{2}+X_{i}\prime \beta_{3}+F_{ij}\prime \beta_{4})(1-W)+(\gamma_{0}+P_{ij}\prime \gamma_{1}+A_{j}\prime \gamma_{2}+X_{i}\prime \gamma_{3}+F_{ij}\prime \gamma_{4})W+\varepsilon_{ij}<T_{k})$$ (1)

The elements in Eq 1 are defined as follows:

- For Survey 1, *Y*_*ij*_ represents the amount that survey respondent *i* stated that they would give to hypothetical other person *j* (i.e., 0, 1, …, 9, or 10), while for Survey 2, *Y*_*ij*_ represents the MRS interval (i.e., below $\frac{1}{15}$, …, or above $\frac{15}{1}$) for survey respondent *i* with respect to hypothetical other person *j*;
- *k* reflects the level chosen, and *k* goes from 0 to 10 for Survey 1 and 1 to 17 for Survey 2 (given its 17 intervals);
- *T*_*k*_ are threshold parameters that are estimated in the model;
- *P*_*ij*_ reflects a vector of 6 categories of social proximity between *i* and *j* (with foreign stranger as the base category);
- *A*_*j*_ is a vector of 9 age categories for hypothetical person *j* (with age 80 and older omitted as the base category);
- *X*_*i*_ is a vector of characteristics of person *i* (including, in our main analysis, gender, age, age-squared, and a vector of categorical measures of distance between birth location and current state of residence, where the base case is a respondent who resides in his birth state. Other indicators are: whether the respondent was born outside the U.S. or in a state whose population-weighted centroid is less than 500, 501 to 1,000, or greater than 1,000 miles from the population-weighted centroid of the respondent’s state of residence. State population centroids are taken from the 2010 U.S. Census (https://www.census.gov/geo/reference/centersofpop.html);
- *F*_*ij*_ is a vector of framing effects;
- *W* is an indicator variable that equals 1 if the question is asking about wealth altruism as opposed to health altruism; and
- *ε*_*ij*_ is assumed to be normally distributed.

A positive value for the coefficient indicates that the characteristic is positively associated with a higher level of altruism (holding constant the other characteristics). β and γ coefficients respectively correspond to questions about health and wealth altruism, and we evaluate the equivalence of the β and γ coefficients for each independent variable. Our hypothesis, based on the prior literature and theory discussed above, is that altruism will be lowest for foreign strangers given the low likelihood of reciprocation (and thus each of the *β*_1_ and *γ*_1_ coefficients should be negative) and highest for immediate family (given kinship altruism). We do not have strong theoretical prior beliefs for the likely signs of the other coefficients, although the prior empirical literature suggests that females exhibit higher level of altruism.

We did not ask survey questions that would allow us to measure and directly control for risk preferences or health status, but we included a variety of variables that might be correlated with these characteristics to our regressions in an attempt to control for them as proxies. These included things such as household income, educational level, and self-reported understanding of probability. The inclusion of these variables did not substantively change the results, which can be found in the supporting information files (S1 Table).

Nonetheless, variation in risk preference may not be perfectly controlled with the inclusion of these additional variables. Consequently, the lack of control for risk preference may cause bias. This omitted variable bias is a particular problem for our ability to assess hypothesis 6 as people who have moved farther away from their birth state may have more tolerance for risk. Likewise, since we have cross-sectional data, the effect of “age” may in fact be due to differences in risk preference across birth cohorts rather than age *per se*. Lack of control for risk preferences does not affect our ability to evaluate hypotheses 1, 2, and 4 as social proximity, priming factors, and the receiver’s age were randomly assigned to the survey respondent (and thus would be uncorrelated with risk preferences); nor does it affect our ability to assess the effect of the giver’s gender, hypothesis 5, as risk preferences are unlikely to affect the respondent’s stated gender (while causation could flow the other way; gender may cause differences in risk preference).

For a broader discussion of risk preferences, altruism, and related subjects, see Hayashi, Altonji, and Kotlikoff [37]; Andreoni and Vesterlund [38]; Cowell and Schokkaert [39]; Carlsson, Daruvala, and Johansson-Stenman [40]; Fehr [41]; and Buurman, Delfgaauw, Dur, and Van den Bossche [42].

## Results

### Distribution of health and wealth altruism

In the dictator game, the mean amount given (out of 10 possible) was 4.34 for health and 2.77 for wealth. The mean marginal rate of substitution implied by the series of dichotomous choices, which could range from $\frac{1}{20}$ to $\frac{20}{1}$, was 3.00 for health and 1.16 for wealth (median values are less than 1 for both). Full descriptive statistics for the observations that are included in the ordered probit regressions are shown in Table 2.

**Table 2:** Descriptive statistics for variables included in ordered probit regressions.

	Survey 1: Dictator Game				Survey 2: Dichotomous Choices			
	Health		Wealth		Health		Wealth	
	Mean	s.d.	Mean	s.d.	Mean	s.d.	Mean	s.d.
**Amount Given by Dictator**	4.34	3.40	2.77	2.87				
**MRS Implied by Dichotomous Choices**					3.00	5.97	1.16	3.37
**Characteristics of the Other Person**								
Social Proximity:								
Immediate Family Member	7.9%		7.9%		9.4%		8.8%	
Extended Family Member	15.8%		14.8%		13.0%		14.1%	
Close Friend	5.1%		5.5%		4.9%		4.5%	
Co-Worker	3.3%		3.8%		3.9%		3.8%	
Acquaintance	9.9%		8.1%		8.5%		8.8%	
Stranger: U.S.	28.4%		32.0%		29.9%		29.1%	
De	29.6%		27.9%		30.5%		30.9%	
Age of other person:								
0–4	6.9%		5.8%		7.0%		7.2%	
5–9	7.1%		7.7%		7.3%		7.3%	
10–17	9.2%		9.3%		7.7%		7.7%	
18–29	10.2%		12.0%		11.1%		12.0%	
30–39	13.1%		9.0%		12.7%		11.3%	
40–49	13.4%		12.1%		12.9%		13.3%	
50–59	12.3%		11.8%		12.1%		11.7%	
60–69	11.2%		12.5%		11.2%		11.5%	
70–79	9.1%		12.1%		9.5%		9.6%	
80 and older	7.5%		7.9%		8.5%		8.6%	
**Characteristics of the Respondent**								
Female	0.52		0.52		0.51		0.52	
Age	46.40	17.43	46.44	17.45	47.12	17.48	47.04	17.49
Live in Same State as Birth State	41.6%		41.9%		45.8%		46.2%	
Live in Different State from Birth, Within 500 Miles	21.9%		21.9%		23.6%		23.5%	
Live in Different State from Birth, 500–999 Miles	11.1%		10.9%		9.1%		9.0%	
Live in Different State from Birth, 1000+ Miles	13.7%		13.7%		10.6%		10.5%	
Born Outside U.S.	11.7%		11.6%		10.9%		10.7%	
**Framing Effects**								
Asked safety altruism questions first	44.4%		44.7%		50.1%		50.8%	
Other person placed on left (before the respondent)	52.5%		52.2%		49.0%		48.2%	
Initially asked about an equal allocation:					33.7%		32.0%	
Initially asked about a less generous allocation					32.8%		34.8%	
Initially asked about a more generous allocation					33.5%		33.2%	
**Number of Observations Contributed to the Regression**	4,906		4,923		5,302		5,532	
**Number of Respondents Contributed to the Regression**	496		496		1,087		1,117	

**Fig 1:**
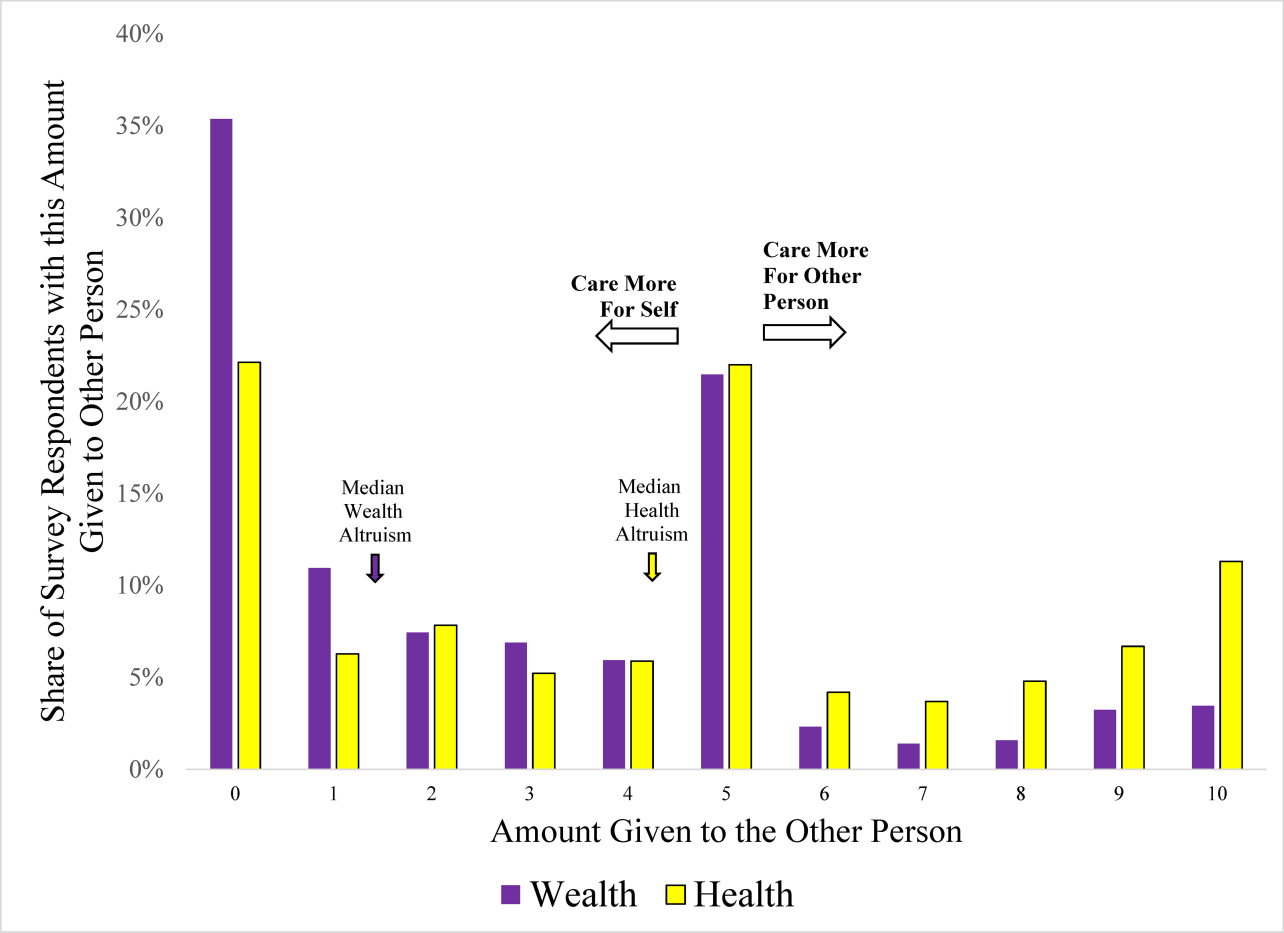
Distributions of levels of health and wealth altruism (survey 1).

**Fig 2:**
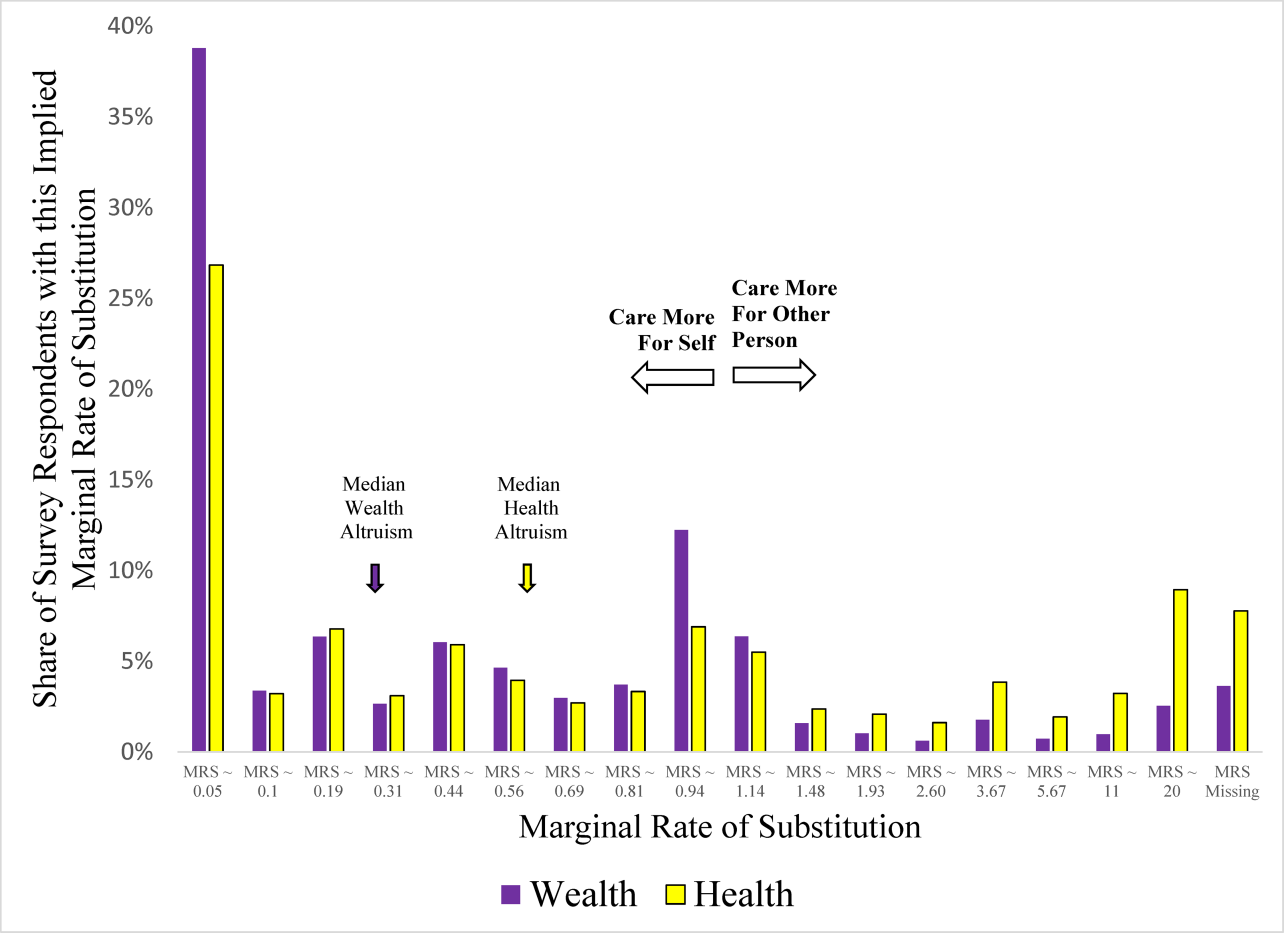
Distributions of levels of health and wealth altruism (survey 2).

Fig 1 and Fig 2 show the unconditional distributions of levels of health and wealth altruism for Surveys 1 and 2, respectively. These figures graphically show the distribution of the dependent variables used in the subsequent regressions, and illustrate that the median respondent demonstrated (not surprisingly) more care for self than the other person. These figures also illustrate that the two surveys yielded qualitatively similar results. For both surveys, we find higher levels of health altruism than wealth altruism. Respondents were far more likely to exhibit no wealth altruism in the dictator game (i.e. by giving none of the scratch-off lottery tickets to the other person) than no health altruism (i.e. by giving none of the medical products or safety inventions to the other person); 22% (35%) give nothing to the other person regarding health (wealth). Similarly, for 27% (39%) of respondents the MRS was less 1/15 with regard to health (wealth), indicating that the individual would prefer having 1 given to self over 15 given to the other person.

Table 3 shows the mean amounts given to the other person in the dictator game and the median MRS by age of the other person and social proximity. We exclude from this table the means/medians that are computed based on less than 10 responses for a particular age/relationship type. For example, we do not show the mean responses for the two individuals in Survey 1 who reported who having a close-friend age 0–4 and who were asked about their preferences for allocation of medical/safety devices to a person of this type. Note that when the level is 5 for Survey 1 and 1 for Survey 2 that would reflect a level of altruism such that the respondent treated self and the other person equally. As expected, respondents generally exhibited the highest level of altruism with respect to immediate family members and the least with respect to foreign strangers. Health altruism is highest for young children and tended to decline as the age of the recipient increased regardless of the recipient’s social proximity to the giver. Conversely, wealth altruism appears to be lowest for young children and higher toward immediate family members in older age groups, though this trend is not clear for other relationships. Caution is warranted in reading too much into the lower apparent wealth altruism towards young children; the lower amount of giving may reflect aversion to giving young children scratch-off tickets rather than aversion to directly giving kids money.

**Table 3:** Levels of health and wealth altruism by age of other person and social proximity.

		Health Altruism										Wealth Altruism									
		Age of Other Person										Age of Other Person									
		0–4	5–9	10–17	18–29	30–39	40–49	50–59	60–69	70–79	80+	0–4	5–9	10–17	18–29	30–39	40–49	50–59	60–69	70–79	80+
		Mean Amount Given By Respondent (Survey 1)																			
Relationship to Other Person	Immediate Family	5.4	5.9	6.0	5.5	5.6	4.9	5.8	5.4	4.9	4.1	3.6		4.6	3.6	4.2	4.5	3.0	3.1	3.5	2.4
	Extended Family	5.7	4.9	5.1	4.4	5.4	5.6	4.7	5.7	4.9	4.9	2.1	2.7	3.3	3.3	3.8	2.8	3.3	3.1	3.3	3.1
	Close Friend				5.3	5.8	4.2	5.5	4.5	3.4					4.3	3.2	3.4	3.6	3.8	3.3	
	Co-Worker				4.2	4.1	3.7	3.7	4.6						3.3	3.0	2.7	3.0			
	Acquaintance			4.2	4.2	4.4	3.9	4.5	4.1	4.6	2.7		1.5	2.0	2.9	2.7	2.3	2.7	3.0	2.5	2.0
	Stranger in U.S.	4.7	4.4	3.7	3.9	3.9	4.1	3.7	4.4	4.3	3.6	2.4	2.4	2.7	2.5	1.9	2.5	2.8	2.8	2.5	3.0
	Stranger in Another Country	4.1	4.2	3.7	3.9	3.6	3.5	3.8	3.8	4.1	3.7	2.1	2.3	2.7	2.4	2.6	2.5	1.9	2.6	2.3	1.9
		Median Marginal Rate of Substitution (Survey 2)																			
Relationship to Other Person	Immediate Family	3.7	11.0	0.9	1.1	0.9	1.1	0.9	1.1	0.9	0.4		0.8	0.8	0.8	0.9	0.9	0.9	0.8	0.4	0.9
	Extended Family	1.5	3.7	0.8	0.6	0.8	0.8	0.9	0.9	0.8	0.4	0.4	0.3	0.4	0.6	0.4	0.4	0.4	0.6	0.6	0.6
	Close Friend				0.8	0.8	0.8	0.7	0.4	0.7	0.2				0.9	0.7	0.7	0.7	0.7	0.2	0.1
	Co-Worker				0.2	0.4	0.3	0.4	0.2						0.3	0.3	0.3	0.1	0.2		
	Acquaintance			0.9	0.6	0.3	0.3	0.4	0.6	0.6	0.4			0.1	0.2	0.1	0.1	0.1	0.1	0.2	0.2
	Stranger in U.S.	0.3	0.7	0.4	0.7	0.6	0.4	0.6	0.6	0.4	0.4	0.2	0.1	0.1	0.3	0.3	0.3	0.1	0.3	0.4	0.2
	Stranger in Another Country	0.9	0.4	0.4	0.4	0.2	0.3	0.3	0.2	0.4	0.2	0.1	0.1	0.1	0.2	0.1	0.1	0.2	0.2	0.2	0.1

Note that since we only ask the respondents to address questions about types of relationships (by social proximity and age of the other person) that the respondent actually has, we consequently asked fewer questions for some categories. For example, fewer questions were asked about close friends and co-workers because when we randomly selected an age range for the other person, if the respondent did not have a close friend, for example, in this age range, the question was skipped and we drew the next combination. That process led to more questions being asked about strangers, because strangers could be of any age group. The age of the survey respondents ranged from 18 to 87 in Survey 1 and 18 to 94 in Survey 2. Finally, note that less than half of the survey respondents currently reside in their birth state.

### Extent to which levels of altruism is situationally versus individually varying

In Table 4, we group respondents into those who never responded altruistically to any of the health questions; those who were sometimes at least somewhat altruistic in giving the medical products / safety inventions to the other person, but who never favored the other person over themselves in the allocation; and those who sometimes favored the other person. We use the same groupings for the wealth questions and produce the cross-tabs shown in Table 4. While the diagonals are strong here (54% and 50% of the samples are along the diagonal for Surveys 1 and 2 respectively), there are notable exceptions. For example, among the 13% of the Survey 1 sample who *never* reported willingness to give any wealth (i.e., scratch-off tickets) to the other person across the 10 situations presented, nearly half (6% out of 13%) sometimes gave more than half of their endowment of medical products / safety inventions to the other person. This result suggests that situations do matter, even though most of the variation in altruistic behavior is across individuals.

**Table 4:** Consistency of the respondent’s levels of altruism across contexts.

		**With Regard to Wealth, Share Who Are:**			
		**Never Altruistic**	**Sometimes Modestly Altruistic, but Never Very Altruistic**	**Sometimes Very Altruistic**	**Row Sum**
**With Regard to Health, Share Who Are:**	**Never Altruistic**	4% (21)	2% (11)	0% (2)	7% (34)
	**Sometimes Modestly Altruistic, but Never Very Altruistic**	2% (11)	27% (135)	2% (10)	31% (155)
	**Sometimes Very Altruistic**	6% (31)	33% (161)	23% (115)	62% (307)
	**Column Sum**	13% (63)	62% (307)	25% (126)	100% (496)
**Survey 2: MRS Implied by Dichotomous Choices**					
		**With Regard to Wealth, Share Who Are:**			
		**Never Altruistic**	**Sometimes Modestly Altruistic, but Never Very Altruistic**	**Sometimes Very Altruistic**	**Row Sum**
**With Regard to Health, Share Who Are:**	**Never Altruistic**	8% (85)	4% (42)	2% (24)	14% (152)
	**Sometimes Modestly Altruistic, but Never Very Altruistic**	4% (44)	13% (140)	4% (48)	21% (231)
	**Sometimes Very Altruistic**	8% (86)	27% (293)	29% (316)	64% (696)
	**Column Sum**	20% (215)	44% (475)	36% (389)	100% (1079)

Notes: Weighted frequencies are shown in parentheses. For Survey 1 (2), "Never Altruistic" is defined as always giving 0 to the other person (MRS always <1/15) regardless of the age or social proximity of the other person, "Sometimes Modestly Altruistic, but Never Very Altruistic" is defined as sometimes giving more than zero (MRS sometimes >1/15), but never giving more than half to the other person (MRS always <1), and "Sometimes Very Altruistic" is defined as sometimes giving more than half to the other person (MRS sometimes >1).

A more precise method to evaluate the relative contributions of situational variability versus individual variance is presented in Table 5. In this analysis, we run our ordered probit regression shown in Eq 1, however with *X*_*i*_ (characteristics of person *i*) and *F*_*ij*_ (framing effects) omitted and replaced with a vector of individual respondent fixed effects. We first evaluate the extent to which the model can reduce the baseline log-likelihood (i.e., McFadden’s Pseudo R-Squared [43]); these Pseudo R-Squared values are 22% and 19% respectively. The corresponding R^2^ values for ordinary least squares regressions, rather than ordered probit regressions, are 47% and 59% for the two surveys respectively. We then repeat the ordered probit regression with just including individual fixed effects, individual fixed effects plus *W* (wealth indicator), individual fixed effects plus *P*_*ij*_ (social relationship), and individual fixed effects plus *A*_*j*_ (age of the other person). We find that roughly four-fifths of the Pseudo R-Squared is achieved solely by the inclusion of individual fixed effects; that is, the vast majority of explained variation in altruism is between rather than within individuals. Of the remainder, the most important situational factor is the nature of the question (health versus wealth), which contributes roughly a 10^th^ of the explained variation in altruism. The social relationship of the respondent with the hypothetical other person contributed 8% of the explained variation in altruism in both surveys, while the age of the other person was a very small contributor (less than 1%) for both surveys.

**Table 5:** Extent to which altruism is explained by individual variation and context.

	Survey 1: Amount Given in Dictator Game	Survey 2: MRS Implied by Dichotomous Choices
**Pseudo R-Squared From Full Model** (i.e., share of baseline log likelihood explained by individual variation and full context):	22.1%	18.6%
**Of this Pseudo R-Squared, share explained by:**		
Individual Variation:	77.4%	82.6%
Individual Variation + Nature of the Question (i.e., Health or Wealth):	90.4%	90.3%
Individual Variation + Social Relationship with the Other Person:	85.1%	90.1%
Individual Variation + Age of the Other Person:	78.1%	83.3%

Notes: "Individual variation" identified by a model that includes dummy variables for individual survey respondents. "Full context" means a model that includes interactions of the "Nature of the Question" with "Social Relation with the Other Person" and the interactions of the "Nature of the Question" with "Age of the Other Person".

### Ordered probit results: Predictors of altruistic sentiments

Table 6 shows the results of the ordered probit regressions. The estimated *T*_*k*_ parameters are not shown, but available from the authors. Controlling for other factors, respondents in both surveys gave significantly more to all relationship types relative to foreign strangers (except for co-workers, where the differences with foreign strangers were mostly insignificant). The most altruism was exhibited towards immediate family members for both surveys and for both health and wealth questions. At the bottom of Table 6, we report p-values for the tests of joint significances of important groups of variables, and we show that for all four regressions social proximity is a highly significant predictor of levels of altruism. We also show that for most of the social proximity relationships there were insignificant differences in how a given relationship was treated for the health and wealth questions.

**Table 6:** Predictors of the respondent’s level of health and wealth altruism (ordered probit estimated coefficients).

	Survey 1: Amount Given in Dictator Game								Survey 2: MRS Implied by Dichotomous Choices							
	Health			Wealth					Health			Wealth				
	Coef.	(s.e.)	Sig.	Coef.	(s.e.)	Sig.	Sig. of Diff.		Coef.	(s.e.)	Sig.	Coef.	(s.e.)	Sig.	Sig. of Diff.	
**Question is about Wealth**	Baseline			-1.61	0.44	***			Baseline			-0.55	0.23	**		
**Characteristics of the Other Person**																
Social Proximity (relative to Stranger: Rest of World):																
Immediate Family Member	0.54	(0.08)	***	0.54	(0.08)	***			0.62	(0.06)	***	0.55	(0.05)	***		
Extended Family Member	0.43	(0.06)	***	0.33	(0.07)	***			0.48	(0.06)	***	0.35	(0.05)	***	*	
Close Friend	0.49	(0.08)	***	0.49	(0.09)	***			0.30	(0.08)	***	0.45	(0.07)	***		
Co-Worker	0.14	(0.09)		0.31	(0.11)	***			-0.06	(0.08)		0.06	(0.08)			
Acquaintance	0.25	(0.06)	***	0.15	(0.07)	**			0.24	(0.07)	***	0.07	(0.06)		**	
Stranger: U.S.	0.09	(0.04)	**	0.12	(0.04)	***			0.14	(0.04)	***	0.08	(0.04)	**		
Age of other person (relative to age 80 and older):																
0–4	0.20	(0.09)	**	-0.17	(0.11)		**		0.34	(0.09)	***	-0.18	(0.08)	**	***	
5–9	0.16	(0.09)	*	-0.06	(0.08)		*		0.42	(0.09)	***	-0.08	(0.08)		***	
10–17	0.07	(0.09)		0.11	(0.07)				0.23	(0.09)	***	-0.14	(0.08)	*	***	
18–29	-0.02	(0.09)		0.06	(0.06)				0.13	(0.08)		0.08	(0.06)			
30–39	0.15	(0.09)	*	0.01	(0.09)				0.09	(0.07)		-0.04	(0.06)			
40–49	0.04	(0.08)		0.03	(0.07)					0.11	(0.07)		0.06	(0.06)			
50–59	0.09	(0.08)		0.02	(0.06)				0.10	(0.07)		0.00	(0.07)			
60–69	0.19	(0.08)	**	0.07	(0.07)				0.15	(0.08)	**	0.06	(0.07)			
70–79	0.13	(0.09)		0.03	(0.06)				0.14	(0.08)	*	0.05	(0.06)			
**Characteristics of the Respondent**																
Female	0.00	(0.10)		0.06	(0.10)				0.17	(0.06)	***	0.06	(0.06)		**	
Age	-0.054	(0.017)	***	-0.005	(0.017)		***		-0.022	(0.010)	**		-0.005	(0.010)		*
Age^2^	0.00045	(0.00018)	**	0.00004	(0.00018)		**		0.00014	(0.00010)		0.00001	(0.00010)			
Relative to those who reside in birth state:																
Live in Different State from Birth, Within 500 Miles	-0.18	(0.11)		-0.18	(0.12)				0.03	(0.07)		0.00	(0.07)			
Live in Different State from Birth, 500–999 Miles	-0.23	(0.19)		-0.37	(0.17)	**			-0.04	(0.11)		-0.14	(0.10)			
Live in Different State from Birth, 1000+ Miles	-0.20	(0.14)		-0.19	(0.13)				0.03	(0.10)		0.10	(0.10)			
Born Outside U.S.	0.16	(0.21)		0.03	(0.19)				-0.15	(0.10)		-0.05	(0.10)			
**Framing Effects**																
Asked health altruism questions first	0.17	(0.14)		0.05	(0.14)				0.27	(0.07)	***	0.11	(0.07)		**	
Other person placed on left (before the respondent)	0.01	(0.14)		-0.07	(0.12)				-0.08	(0.08)		-0.16	(0.08)	*		
Interaction of above framing issues	0.21	(0.19)		0.15	(0.20)				-0.01	(0.11)		0.05	(0.11)			
Relative to initially asked about an equal allocation:																
Initially asked about a less generous allocation									0.04	(0.04)		0.08	(0.04)	**		
Initially asked about a more generous allocation									-0.10	(0.04)	***	-0.01	(0.04)		*	
**P-Value for Test of Joint Significance of:**	**Health**			**Wealth**			**Combined**		**Health**			**Wealth**			**Combined**	
Relationship	0.0%	***		0.0%	***		0.0%	***	0.0%	***		0.0%	***		0.0%	***
Age of other person	3.2%	**		23.8%			2.5%	**	0.0%	***		0.2%	***		0.0%	***
Age and Age^2^	0.0%	***		91.2%			0.0%	***	0.0%	***		6.4%	*		0.0%	***
Migration from birth location	22.7%			15.3%			31.5%		53.1%			44.6%			49.7%	
Framing Effects	1.9%	**		51.2%			8.9%	*	0.0%	***		0.2%	***		0.0%	***
**Number of Observations in the Regression**	9,829								10,834							
**Number of Respondents in the Regression**	496								1,125							

Note: Robust standard errors are used, clustered by survey respondent. Two-tailed statistical significance at the 1%, 5%, and 10% levels are indicated, respectively, by ***, **, and *.

Health altruism is highest for those age 0–4 and 5–9 in both surveys, while wealth altruism is lower for these age groups, and these differences in treatment for the health and wealth questions are statistically significant. One possible explanation for this sign reversal between health and wealth is that respondents may think that young children are entitled to good health (whereas adults may be held to be more responsible for their own health status), whereas with regard to wealth, individuals may not see children as having an entitlement to wealth. (We thank an anonymous referee for this suggestion). The coefficients for the other age groups do not show consistent, stable patterns, nor significant differences in treatment for the health and wealth questions. Nonetheless, the set of coefficients on age of the other person is jointly significant for three of the four specifications, with the exception of wealth altruism in the dictator game.

Female survey respondents had significantly higher health and wealth altruism as measured in Survey 2, yet gender was an insignificant factor in altruistic sentiments in the Survey 1 regressions.

**Fig 3:**
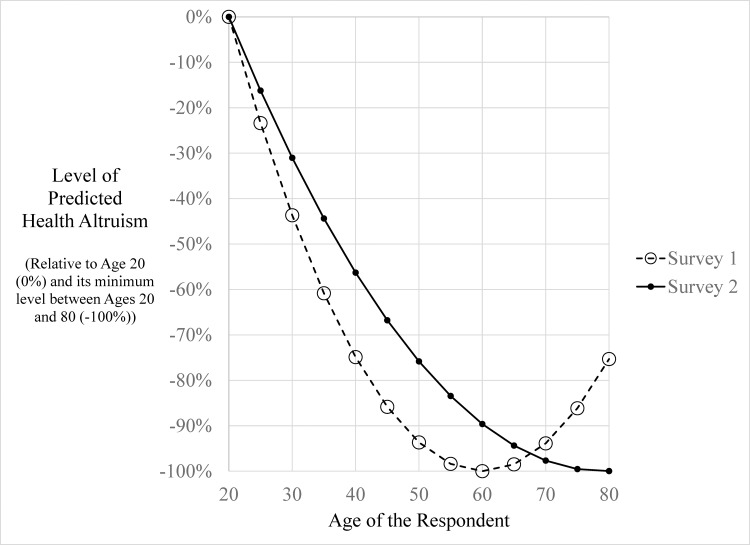
Levels of health altruism by respondent’s age (from ordered probit regression results).

We find interesting patterns of results with respect to the survey respondent’s age. Age and Age-squared were jointly significant factors in three of the four regressions (using a 90% confidence level), with the exception for wealth in the dictator game (although, the coefficient on Age-squared was significant only for the health regression for Survey 1). To better understand the coefficients with respect to health, Fig 3 shows the plot of these coefficients when interacted with age. We compute ${\hat{\beta }}_{Age}Age+{\hat{\beta }}_{{Age}^{2}}{Age}^{2}$ for ages 20 to 80, find the minimum and maximum values for this sum, and then show in Fig 3 the value $\left[Max-({\hat{\beta }}_{Age}Age+{\hat{\beta }}_{{Age}^{2}}{Age}^{2})\right]/[Max-Min]$ – that is, we scale the values so that they lie in the interval of 0% to -100%. We find that in both surveys, health altruism declines with an increase in the age of the respondent and is lowest for respondents who are 60 and 79 years old in Surveys 1 and 2 respectively; after these ages, levels of health altruism modestly increase. One interpretation of these results is that individuals are more sensitive to their own mortality as they reach middle and near retirement age, and thus are less willing to forgo the offered medical products/safety inventions. Yet, above a certain age, as the prospect of imminent death nears, altruistic sentiments increase. (Note that as shown in the supporting information files, prior to completing the survey, as a means of explaining the upcoming survey questions, respondents were presented statistics on the probability of survival for 10 additional years by age using mortality tables from the National Vital Statistics Report) [44]. Alternatively, it should be noted that we cannot separately identify the effects of own age from own birth cohort given the cross-sectional nature of this panel. It could be the case that more recent birth cohorts are more health altruistic than birth cohorts from prior decades.

We find no support for the hypothesis that distance from birth location to current residence influences levels of altruism. Only one of the sixteen estimated coefficients was statistically significant, and the eight included variables were jointly insignificant in both regressions (with p-values of 0.315 and 0.497 respectively). To investigate further, using the data from Survey 1, we conducted an OLS regression of health and wealth altruism using the same variables that are included in Table 6‘s regression except the distance indicators, computed the residuals, plotted them against distance from birth state to current residence state (for the U.S. born), and conducted a non-parametric locally weighted scatterplot smoothing (LOWESS) regression; we found no pattern in these residuals suggesting a relation to distance (i.e., the LOWESS plot was nearly flat throughout the distribution). Despite these null results, a future study could investigate whether changing residences within a state, the number of moves, and the timing and reason for a move affects altruistic sentiments. It might be possible to do incorporate an investigation of altruistic behavior in the context of a future randomized study like the Moving to Opportunity study (Sanbonmatsu et al. [45]) that randomly assigned “Section 8 rental assistance certificates or vouchers that could use only in census tracts with 1990 poverty rates below 10 percent” (p. xiv)–this study found the treatment group developed more social ties with relatively more affluent people and felt safer in their neighborhoods, among other impacts, but the study did not specifically assess the effect of distance or moving on altruistic behaviors.

Four of the sixteen estimated coefficients for the framing effect variables were significant (at the 10% level), and the framing effect variables were jointly significant for both regressions. This result supports the argument that context matters for altruistic behaviors.

In results shown in the supporting information files, we explored specifications that added additional characteristics of the survey respondent. Our main results that are shown in Table 6 are qualitatively robust to the addition of these added controls, which included number of warm-up questions answered incorrectly, self-reported understanding of probability, race/ethnicity, an indicator for being household head, household size, housing type, income, marital status, an indicator for living in a Metropolitan Statistical Area, Census region, work status, and number of kids. Given the large number of variables added, and the correlations between some variables (e.g., income and education), we may have lower power to separately identify the contributions of these variables. Note that while income and education are positively correlated, the correlation is modest; the 0.22 correlation between high school dropout and household income less than $15,000 is the largest correlation between the included income and education indicator variables for the second survey. The correlations between the added variables are, nonetheless, modest enough to yield some notable patterns of significant coefficients. For example, we find that, relative to high school dropouts, all higher education levels are significantly less health altruistic in the dictator game, but more altruistic in the second survey. We do not have an explanation for this divergence. In both surveys for both health and wealth, we find a positive association between the number of warm-up questions answered incorrectly and the amount given to the other person, and many of these coefficients are significant (but not consistently significant across surveys). We do not have a theory for why having more wrong control questions would be positively associated with donations.

## Conclusion

Existing empirical work has investigated the effects of respondent characteristics (such as gender and own age) as well as genetic and social relatedness on altruistic sentiments. This study contributes to this growing body of literature by considering how the age of the other person and nature of the good being sacrificed (i.e., health versus wealth) affect the levels of altruistic sentiment. By also considering survey respondent characteristics such as distance to birthplace, age, and gender, this study provides important insights into how individuals value the health and wealth of other residents.

The results of this study indicate that age (of both the giver and recipient) and social proximity do play roles in the magnitude of altruistic sentiments, though these effects are not linear nor are they equivalent for both health and wealth altruism. Notably, we find more health altruism towards young children, and more wealth altruism towards adults. The results indicate that individuals appear to exhibit less health altruism as they age, with the lowest altruism exhibited from those ages 60 to 79 across our two surveys.

Given the strong effects of the age of the recipient on both wealth and health altruism, and given the fact that using hypothetical contributions, rather than actual contributions, introduces some unknown amount of bias in our results, it would be highly useful for subsequent incentivized studies to reevaluate these findings using actual monetary contributions. For example, a dictator game could be used where the age of the recipient was randomly assigned. It would be highly preferable for such a study to properly control for the giver’s attitude to risk of the respondents (see Krawczyk and Le Lec [33] as a good example for how to control for risk attitudes).

The distance of the respondent to his birthplace was statistically insignificant in both of the surveys conducted for this study. While Americans are moving less than in the past, individuals across the globe are also becoming more tightly integrated thanks to globalization and improvements in communications technology. Thus, it is perhaps not surprising that distance from one’s birthplace did not have a noticeable impact on altruistic sentiments. However, our failure to reject this null hypothesis does not mean that geography does not matter.

Our results are consistent with the theories of kinship and reciprocal altruism. Individuals demonstrate greater degrees of both health and wealth altruism toward members of their immediate family than other groups, which supports the theory of kinship altruism. We also find strong levels of altruism with respect to close friends (much stronger than for acquaintances and strangers), supporting the theory of reciprocal altruism, as reciprocity is more likely to occur between individuals of closer social proximity. Whether or not these sentiments are intended for genetic survival cannot be determined through this study, but the results suggest that social proximity does play a role in an individual’s propensity to sacrifice for another person. The results generally support the notion that individuals are willing to endure some personal sacrifice for group survival beyond that of their gene pool.

It is impossible, with these results alone, to confirm whether or not individuals endure personal sacrifices for reasons beyond self-interest. This study does confirm, however, that individuals tend to express greater degrees of health altruism than wealth altruism. Perhaps respondents are more comfortable sacrificing in order to preserve the health of others than in order to provide a monetary endowment left to spend at the receiver’s discretion. This result is consistent with the findings of studies considering public attitudes toward government assistance, which find that support for cash assistance is much lower on average than support for other types of assistance (i.e. health insurance, educational assistance, and job training) [46].

## Supporting information

S1 File Survey 1 questions. (PDF)

S2 File Survey 2 questions. (PDF)

S1 Fig Question progression for survey 2. (PDF)

S1 Table Predictors of the respondent’s level of health and wealth altruism including additional respondent characteristics (ordered probit estimated coefficients). (PDF)
