# Supplementary material for: Altruism by age and social proximity
Source: PLoS One. 2017 Aug 24;12(8):e0180411. doi: 10.1371/journal.pone.0180411 (PMC5570493; doi:10.1371/journal.pone.0180411)
Supplement: S1 File — (PDF) [file pone.0180411.s001.pdf]

SURVEY 1

Question 1: What is your age? Click on the box next to your age.

- Age 18-29 ☐
- Age 30-39 ☐
- Age 40-49 ☐
- Age 50-59 ☐
- Age 60-69 ☐
- Age 70-79 ☐
- Age 80 or older ☐

SURVEY 1

Question 2: How many living immediate family members do you have in each age group? If you do not know the age of the family member, please give your best guess. If you do not have any immediate family members who are in this age group, please leave the cell blank. The sum of your entries should match the total number of living immediate family members you have of each type.

	Enter the *number* of family members of each type in each cell										
**Type of immediate family member**	**Age 0-4**	**Age 5-9**	**Age 10-17**	**Age 18-29**	**Age 30-39**	**Age 40-49**	**Age 50-59**	**Age 60-69**	**Age 70-79**	**Age 80 or older**	**Sum of your entries**
Your parents (including mother, father, stepmother, stepfather)											
Your siblings (including brothers, sisters, step-siblings, half-siblings)											
Your spouse or partner (including long-term relationships)											
Your children (including biological and adopted children)											

SURVEY 1

Question 3: How many living extended family members do you have in total by type?
Enter the TOTAL NUMBER of living extended family members you have of each type. If you do not know the exact number, please give your best guess. If you do not have any family members living in this category (for example, if your grandparents are not living or you have never had children), then enter "0".

**Type of extended family member**	**Enter the *TOTAL NUMBER* of each type in each cell**
Your grandparents<br>(your parents' / stepparents' biological parents)	
Your aunts and uncles<br>(your parents' / stepparents' siblings and their spouses)	
Your cousins<br>(children of your aunts and uncles)	
Your siblings' spouse(s)<br>(your sisters-in-laws & brothers-in-law via your siblings)	
Your siblings' children<br>(your nieces and nephews)	
Your spouse or partner's parents<br>(your mother-in-law, father-in-law)	
Your spouse or partner's siblings<br>(your sisters-in-law & brothers-in-law via your spouse)	
Your children's spouses<br>(your daughters-in-law, sons-in-law)	
Your grandchildren<br>(your children's children)	

SURVEY 1

Question 4: Do you have ANY living extended family members in each age group? Click on the box if you have ANY living extended family members in this age group. Please give your best guess if you are not sure of extended family members' ages.

	Age 0-4	Age 5-9	Age 10-17	Age 18-29	Age 30-39	Age 40-49	Age 50-59	Age 60-69	Age 70-79	Age 80 or older	*TOTAL NUMBER* of extended family members from question 3.
**Click on the box if you believe the you have *ANY* living extended family members in this cell.**											
**Extended family include your:**	☐	☐	☐	☐	☐	☐	☐	☐	☐	☐	☐

Grandparents,
Aunts and Uncles,
Cousins,
Siblings' Spouse(s),
Siblings' Children (Nephews and Nieces),
Spouse's Parents,
Spouse's Siblings,
Children's Spouse(s), and
Grandchildren

SURVEY 1

Question 5: How many close friends, co-workers, and acquaintances do you have? Enter the TOTAL NUMBER of your close friends, co-workers, and acquaintances. We understand that you are unlikely to know the exact number -- please give your best guess. If you do not have any persons in this category (for example, you have no co-workers as you are unemployed), then enter "0".

**Type of relation**	**Enter the *TOTAL NUMBER* of each type in each cell**
Close Friends<br>(Include non-family members who you have close friendship with. For example, someone you talk with regularly and have a strong friendly bond).	☐
Co-workers<br>(Include anyone you work with regularly who is not a family member or close friend.)	☐
Acquaintances<br>(Include anyone who is not a family member, close friend, or co-worker who you know well enough that you would say "hello" to if you passed them on the street.)	☐

SURVEY 1

Question 6: What are the ages of your close friends, co-workers, and acquaintences? Click on the boxes that best reflect the ages of your close friends, co-workers, and acquaintences. For example, if most of your close friends are aged 30-39 or 40-49, then you would click on both of these boxes in the "Close Friends" row. If your co-workers are equally spread between the ages 18 and 65, you would click all of the boxes in the "Co-workers" row except Aged 0-4, 5-9, 10-17, 70-79, and 80 or older.

	Click on the boxes that best reflect the ages of this type of relation										
**Type of relation**	**Age 0-4**	**Age 5-9**	**Age 10-17**	**Age 18-29**	**Age 30-39**	**Age 40-49**	**Age 50-59**	**Age 60-69**	**Age 70-79**	**Age 80 or older**	***TOTAL NUMBER* of this type from question 5.**
Close Friends (Include non-family members who you have close friendship with. For example, someone you talk with regularly and have a strong friendly bond).											
Co-workers (Include anyone you work with regularly who is not a family member or close friend.)											
Acquaintances (Include anyone who is not a family member, close friend, or co-worker who you know well enough that you would say "hello" to if you passed them on the street.)											

Preview to Questions 7 and 8

The next two sets of questions will ask you to make choices about things where chance or luck plays a role in the outcome.

An example of a chance event is the flipping of a coin. If a coin were tossed 100 times, on average it would land on heads 50 times and land on tails 50 times.

Likewise, if a coin were tossed 1,000 times, on average it would land on heads 500 times and land on tails 500 times.

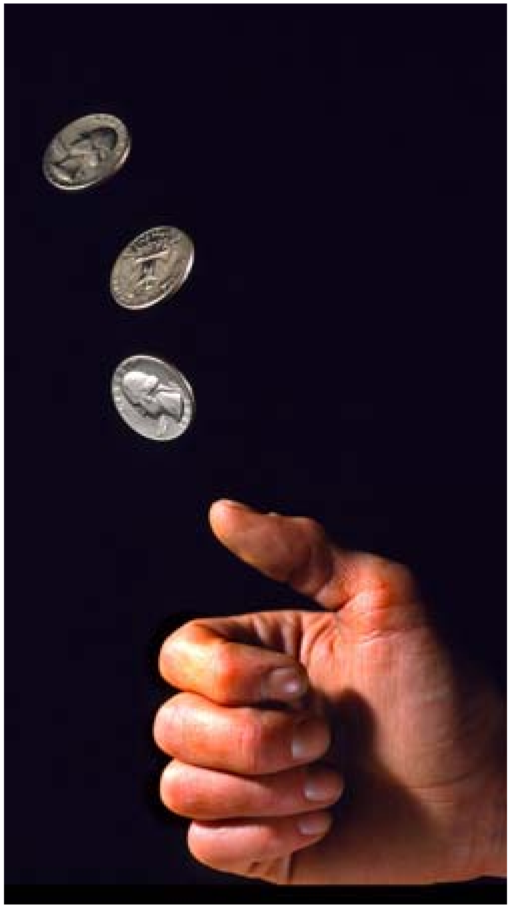

Preview to Questions 7 and 8 (Continued)

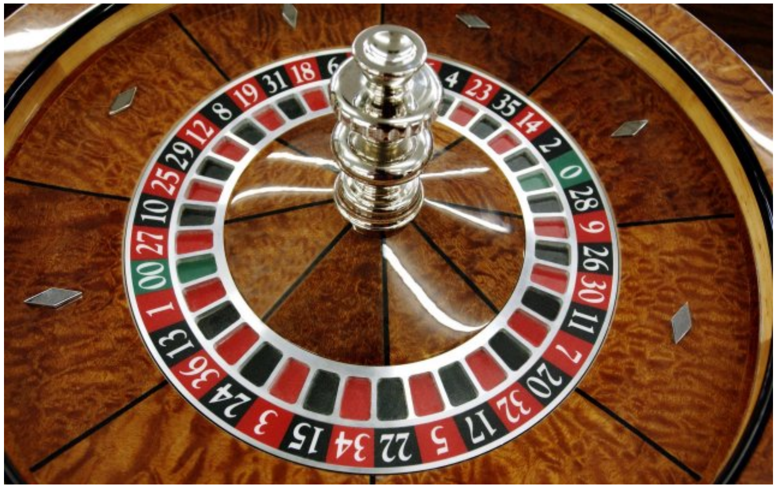

Another example is a roulette wheel.

An American roulette wheel has 38 slots.

The ball should land in any given spot (for example the #7 slot), once out of every 38 spins.

If the roulette wheel were spun 38,000 times, on average the ball will land in the #7 slot 1,000 times.

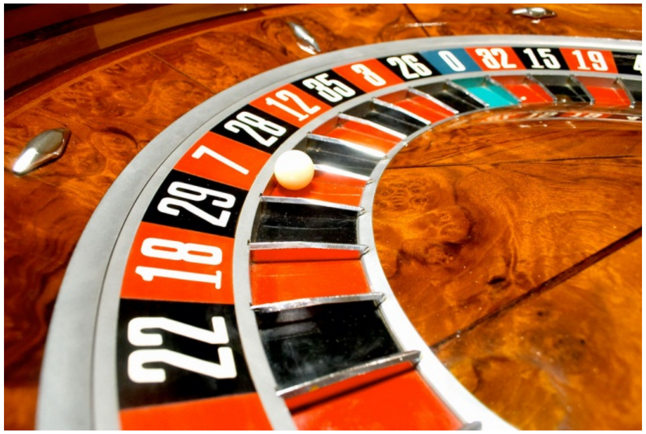

Preview to Question 7: Chance of Dying During the Next 10 Years

Question 7 will include an estimate of the chance of you or others dying during the next 10 years. For example, for a typical 25 year old living in the United States, the chance of dying before age 35 is 12 out of 1,000:

Age **25**: Chance of Dying During Next 10 Years = **12** out of 1,000.

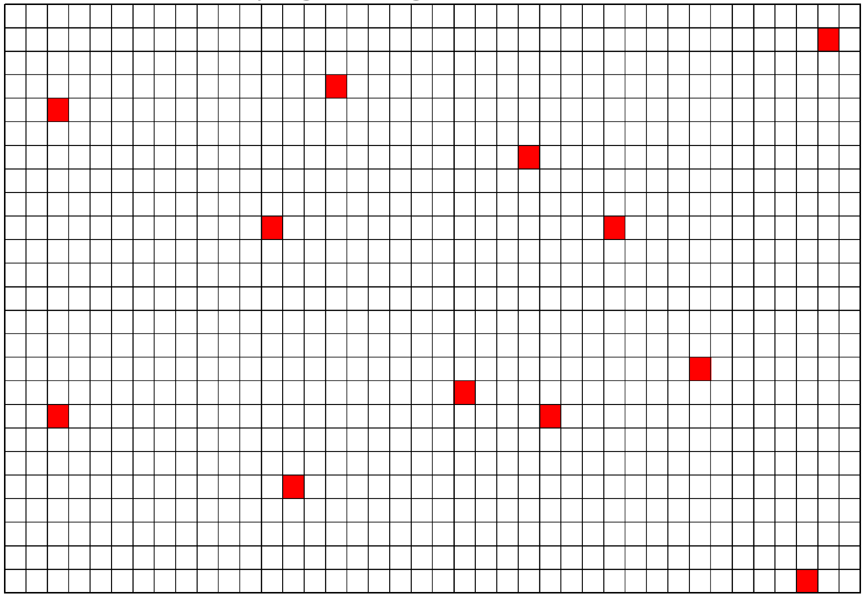

There are 1,000 squares shown. The number of red squares is equal to the number of chances out of 1,000 that the typical person will die within 10 years. You can think of each square like the slot on a roulette wheel, where if by chance the roulette ball landed in a red square, the person would unfortunately die. As shown on the next several pages, as people age, the chance of dying during the next 10 years rises.

Preview to Question 7 (continued)

Age **35**: Chance of Dying During Next 10 Years = **22** out of 1,000.

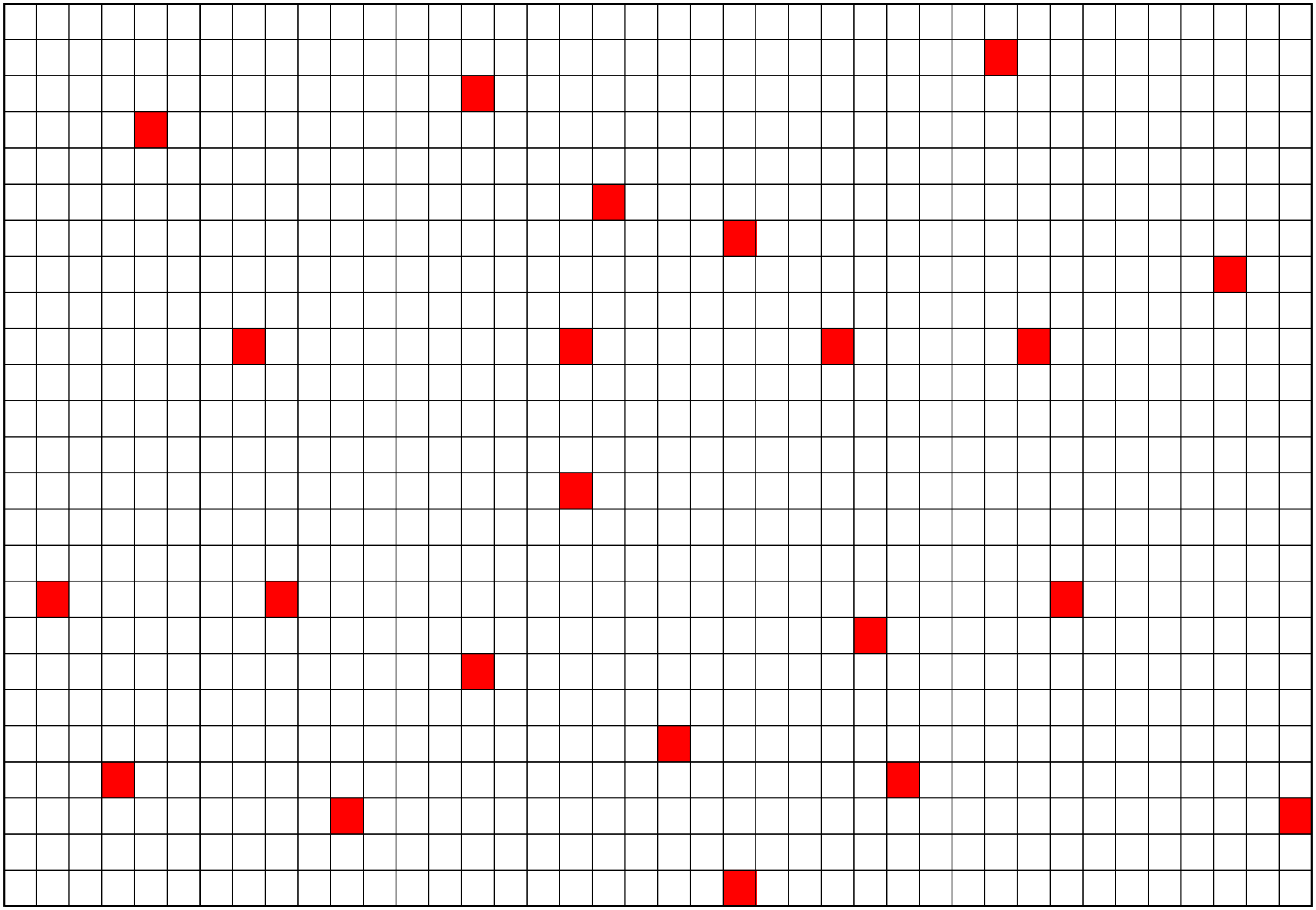

Preview to Question 7 (continued)

Age **45**: Chance of Dying During Next 10 Years = **48** out of 1,000.

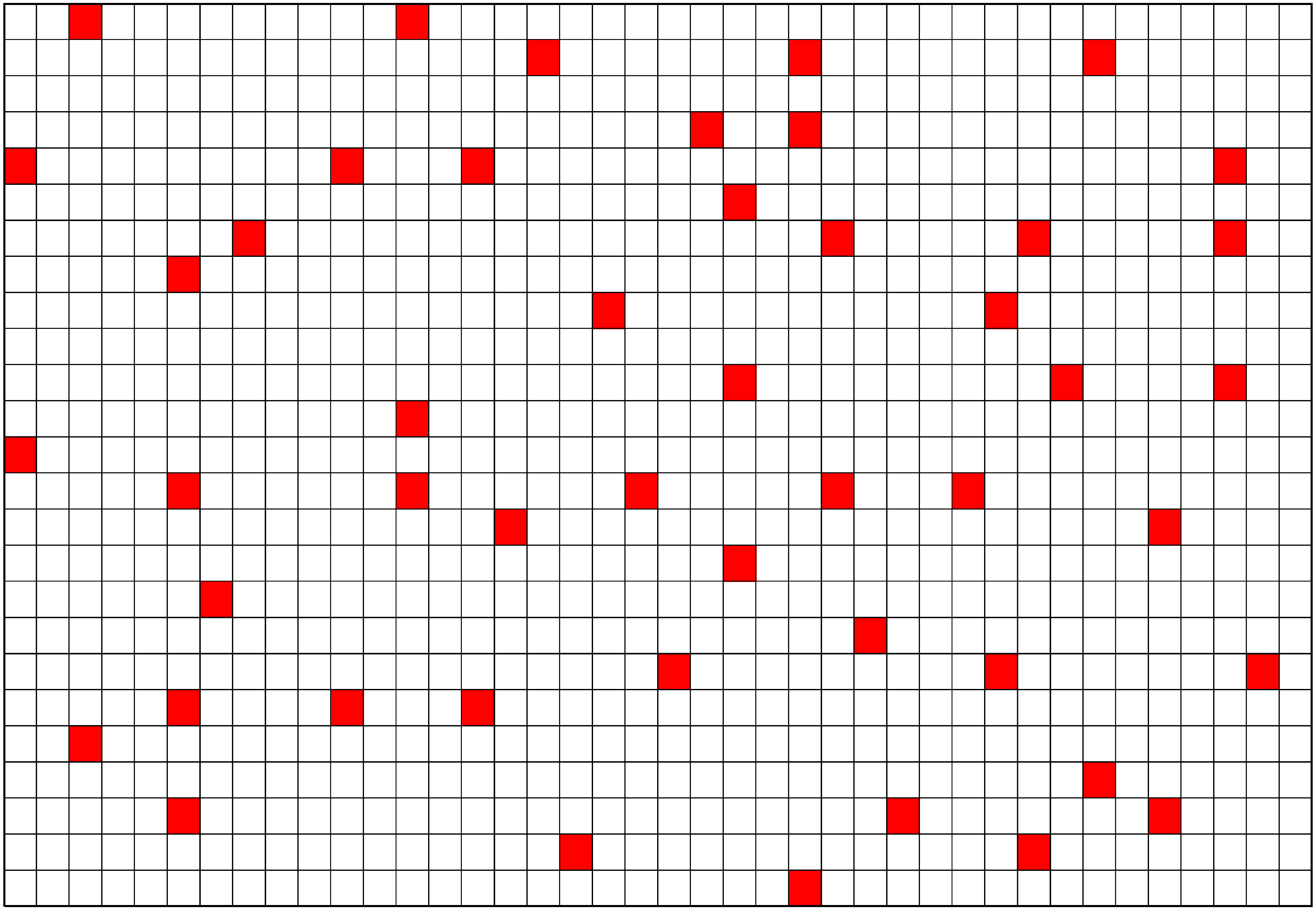

Preview to Question 7 (continued)

Age **55**: Chance of Dying During Next 10 Years = **101** out of 1,000.

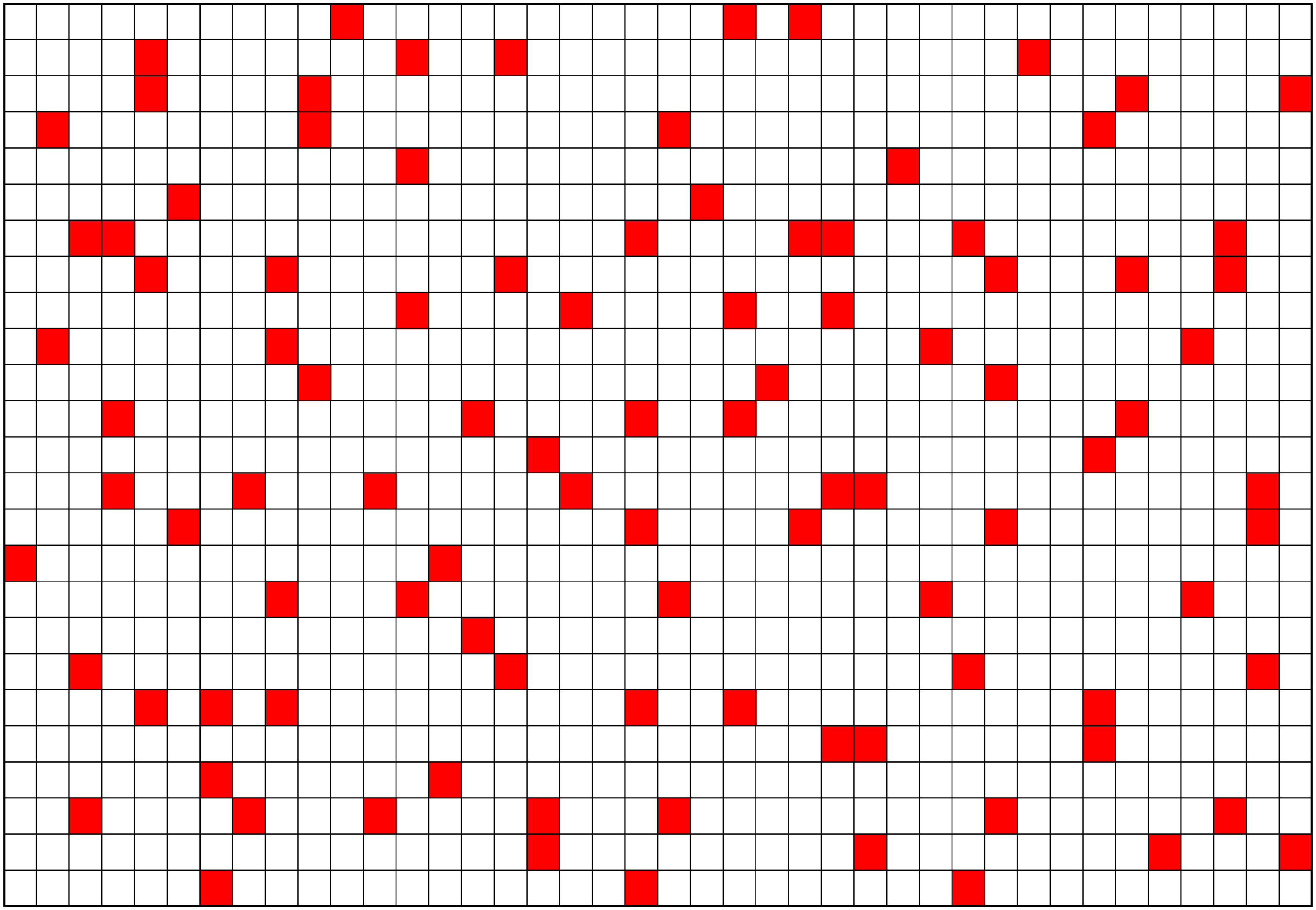

SURVEY 1

<u>Preview to Question 7 (continued)</u>

Age **65**: Chance of Dying During Next 10 Years = **219** out of 1,000.

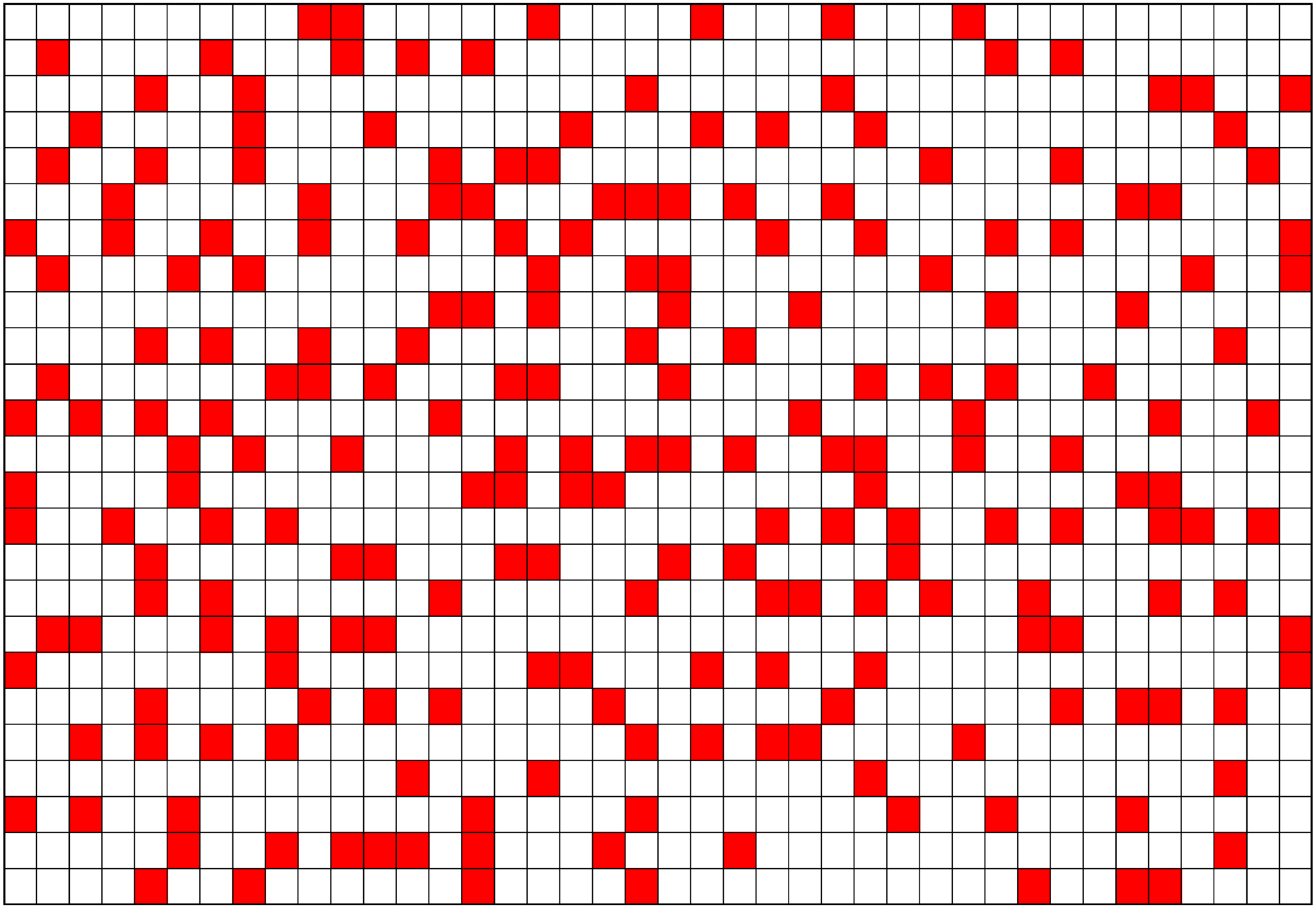

Preview to Question 7 (continued)

Age **75**: Chance of Dying During Next 10 Years = **490** out of 1,000.

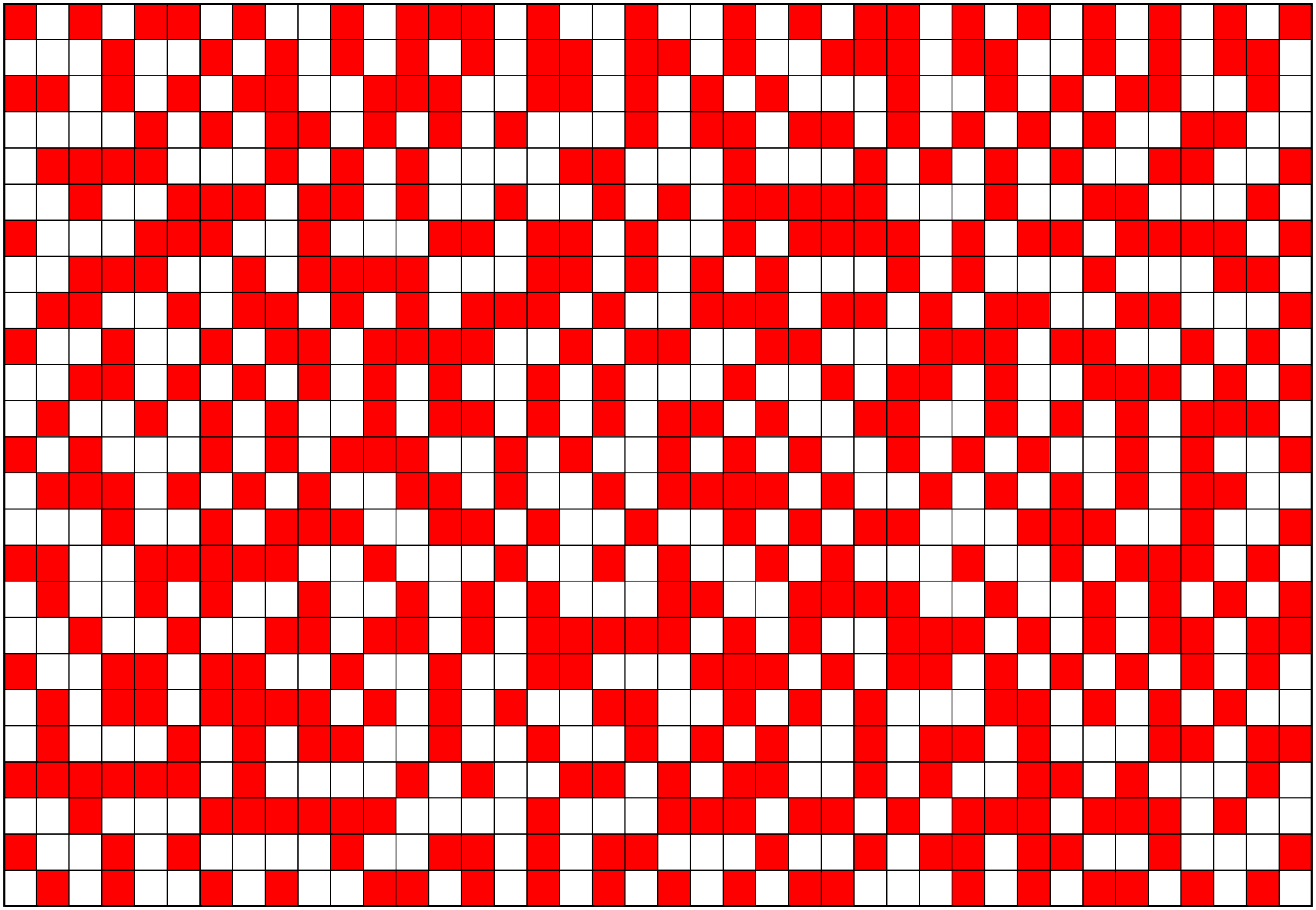

Preview to Question 7 (continued)

Age **85**: Chance of Dying During Next 10 Years = **863** out of 1,000.

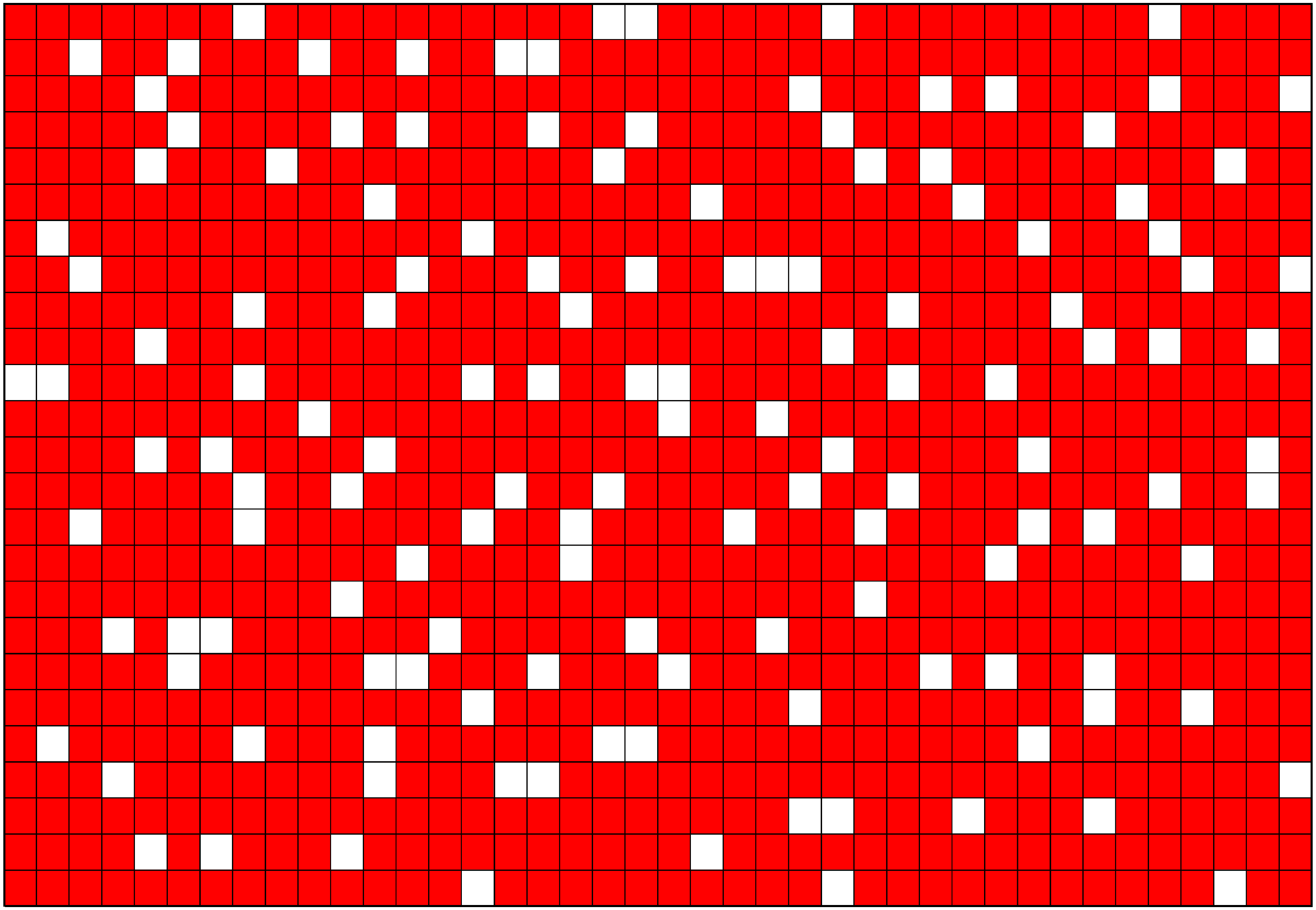

Preview to Question 7 (continued)

Take the case of a 65 year old. Out of one thousand 65 year olds, 219 die before they reach 75 years old. Imagine a roulette ball randomly landing in one of the squares on the grid. If the ball lands in a white square, the person lives to age 75.

Age **65**: Chance of Dying During Next 10 Years = **219** out of 1,000.

(Result: Lives to Age 75)

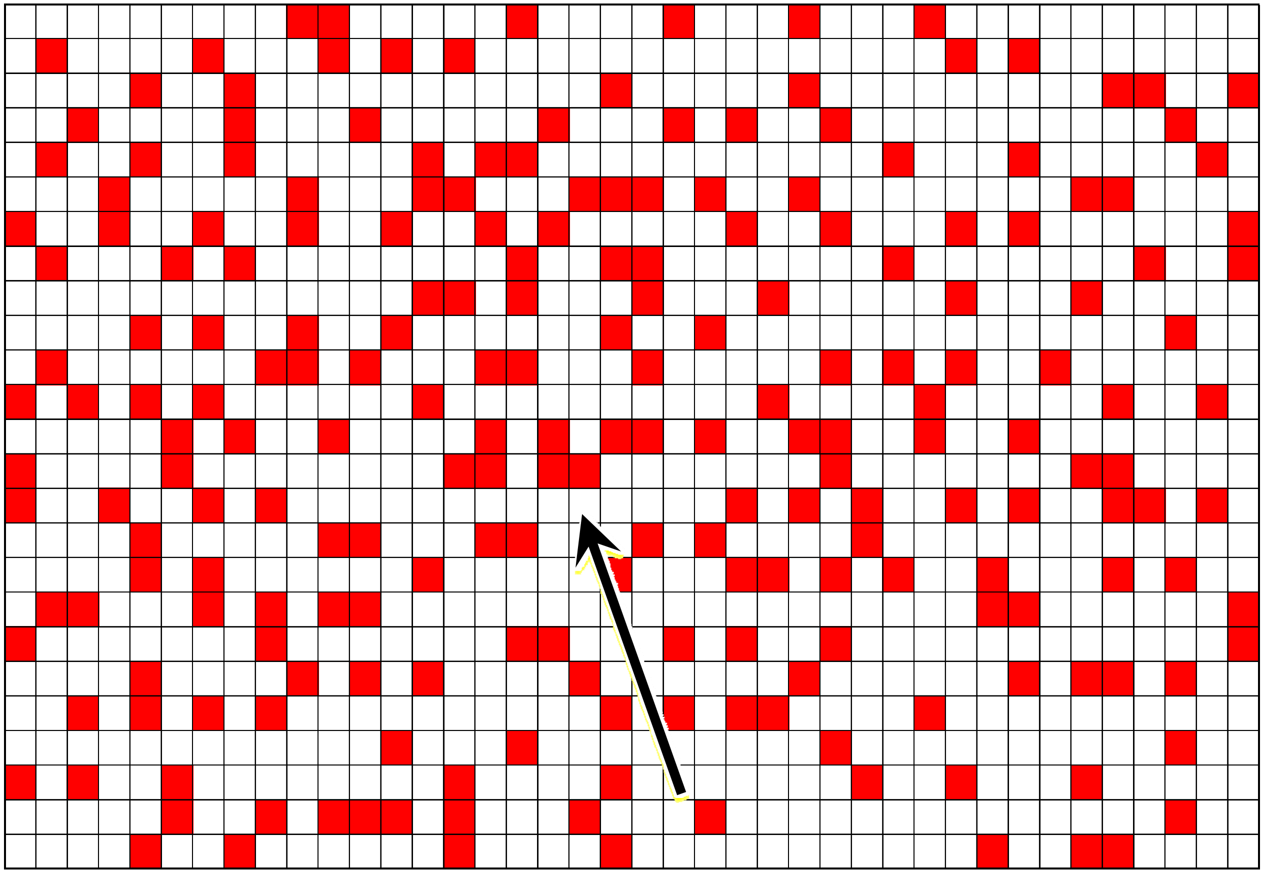

<u>Preview to Question 7 (continued)</u>

If the ball lands in a red square the person dies before age 75. Out of 1,000 spins of the roulette wheel, we would expect the ball to land in a red square 219 times.

Age **65**: Chance of Dying During Next 10 Years = **219** out of 1,000.

(Result: Dies Before Age 75)

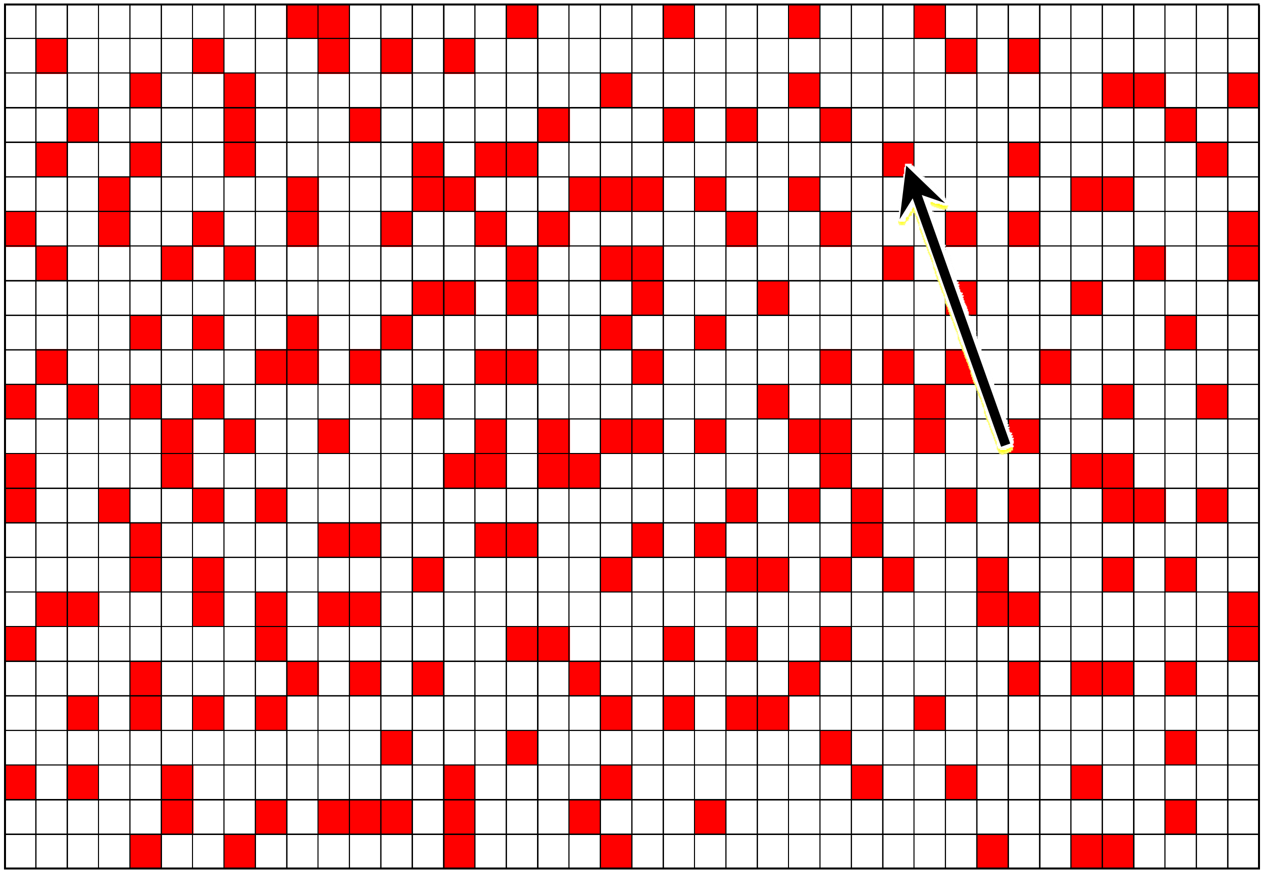

Preview to Question 7 (continued): Medical Products & Safety Inventions

Now, imagine that a company decided to give out special medical products and/or safety inventions that could lower a person's chance of dying during the next 10 years. A medical "product" could include things like drugs, immunizations, and new medical screening technologies that can catch diseases and cancers while they are still treatable. Life-saving inventions include things like car safety devices that lower the risk of serious accidents or technologies that help prevent accidental drowning, poisoning, falling, electrocution, etc. Assume that these products and inventions have no side effects and that these products / inventions are not available on the market and can be obtained only by the company's donation to a person.

Assume that the company has decided to give out 10 of these medical products and/or safety inventions and they will be given to you and/or one other person. Once they are given out, assume that they cannot be given or sold to someone else.

Each product or invention given will lower the recipient's chance of dying during the next 10 years by one chance in 1,000. Thus, if the company gives 5 medical products or inventions to a person, the person's chance of dying during the next 10 years would decline by 5 chances in 1,000.

Preview to Question 7 (continued)

Imagine three medical products or safety inventions are given to a 65 year old. The three products or inventions would lower the person's chance of death before age 75 from 219 out of 1,000 to 216 out of 1,000. The change in the person's chance of death is shown in the following figure. The three blue squares were previously red squares, and reflect the reduction in the chance of death that is caused by the receipt of the three medical products or safety inventions.

Age **65**: Chance of Dying During Next 10 Years = ***216*** out of 1,000 after the donation by the company.

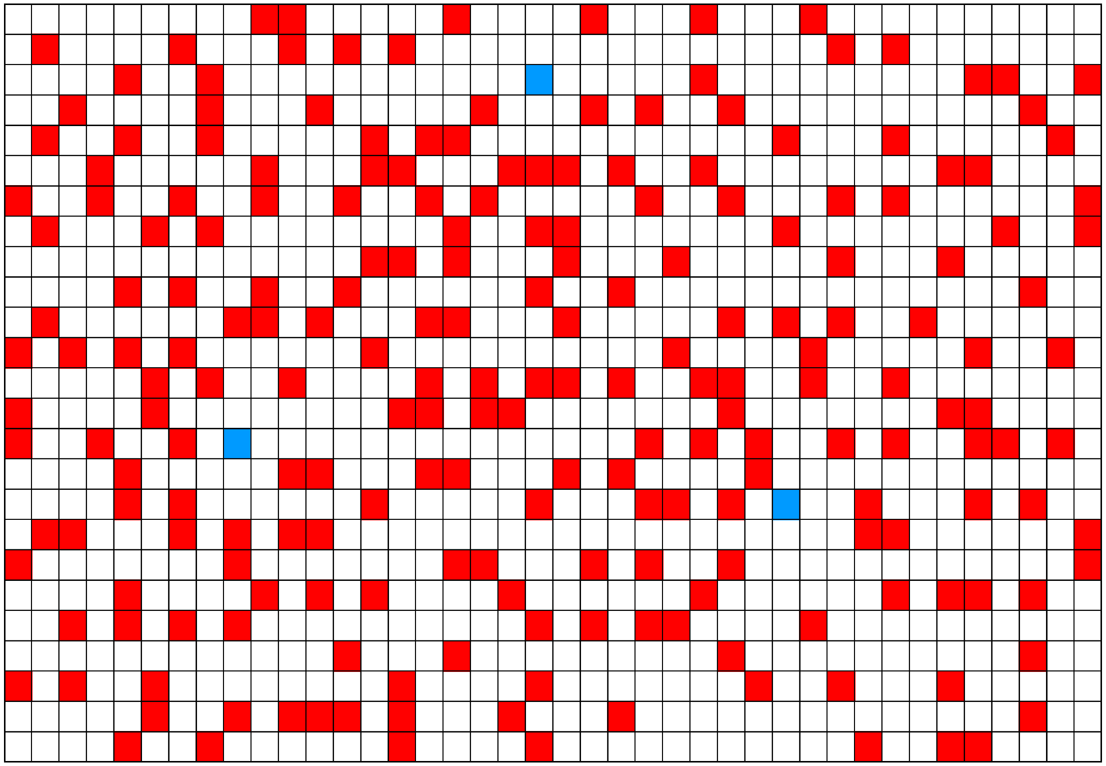

Preview to Question 7 (continued)

If the roulette ball happens to land in one of these three blue squares, then the person's life is extended by the new product or invention.

Age **65**: Chance of Dying During Next 10 Years = **216** out of 1,000.

(Result: Saved by Product)

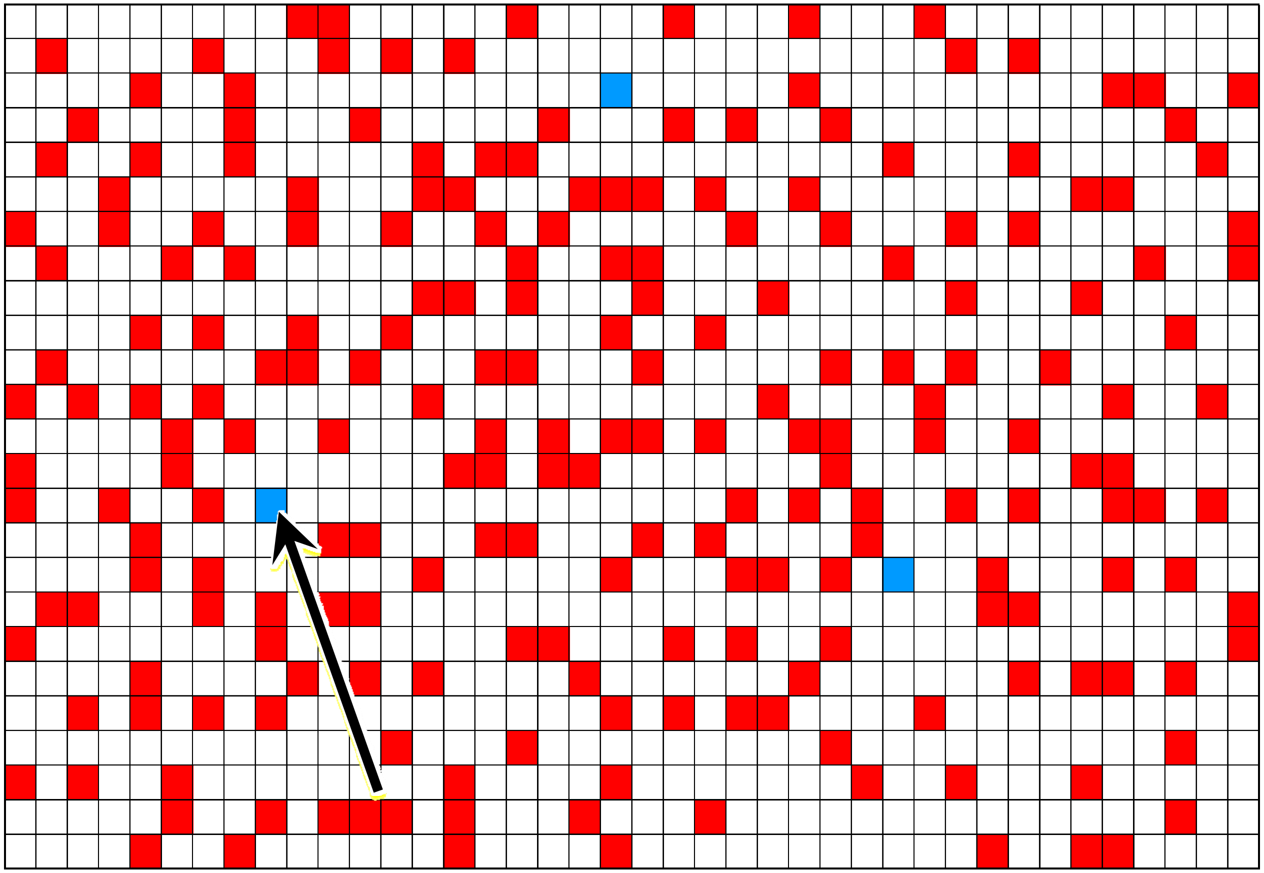

Preview to Question 7 (continued)

If the ball lands in a red square, the person dies before age 75, and this reflects a death that the new product or invention could not prevent.

Age **65**: Chance of Dying During Next 10 Years = **216** out of 1,000.

(Result: Dies Despite Having 3 Products)

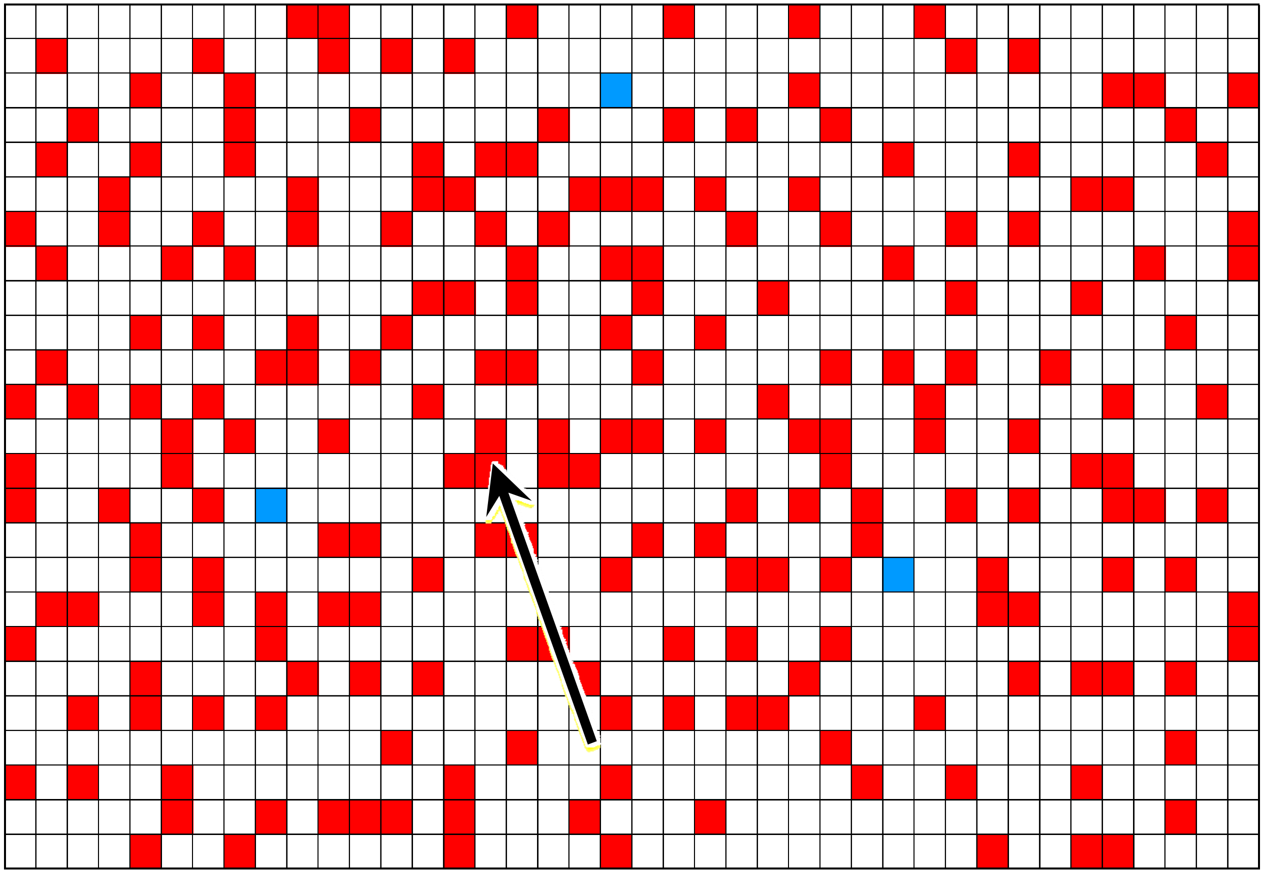

Preview to Question 7 (continued)

Finally, if the ball lands in a white square, the person lives to age 75, for reasons unrelated to having the new product/invention.

Age **65**: Chance of Dying During Next 10 Years = **216** out of 1,000.

(Result: Lives 10 Years Not Due to Products)

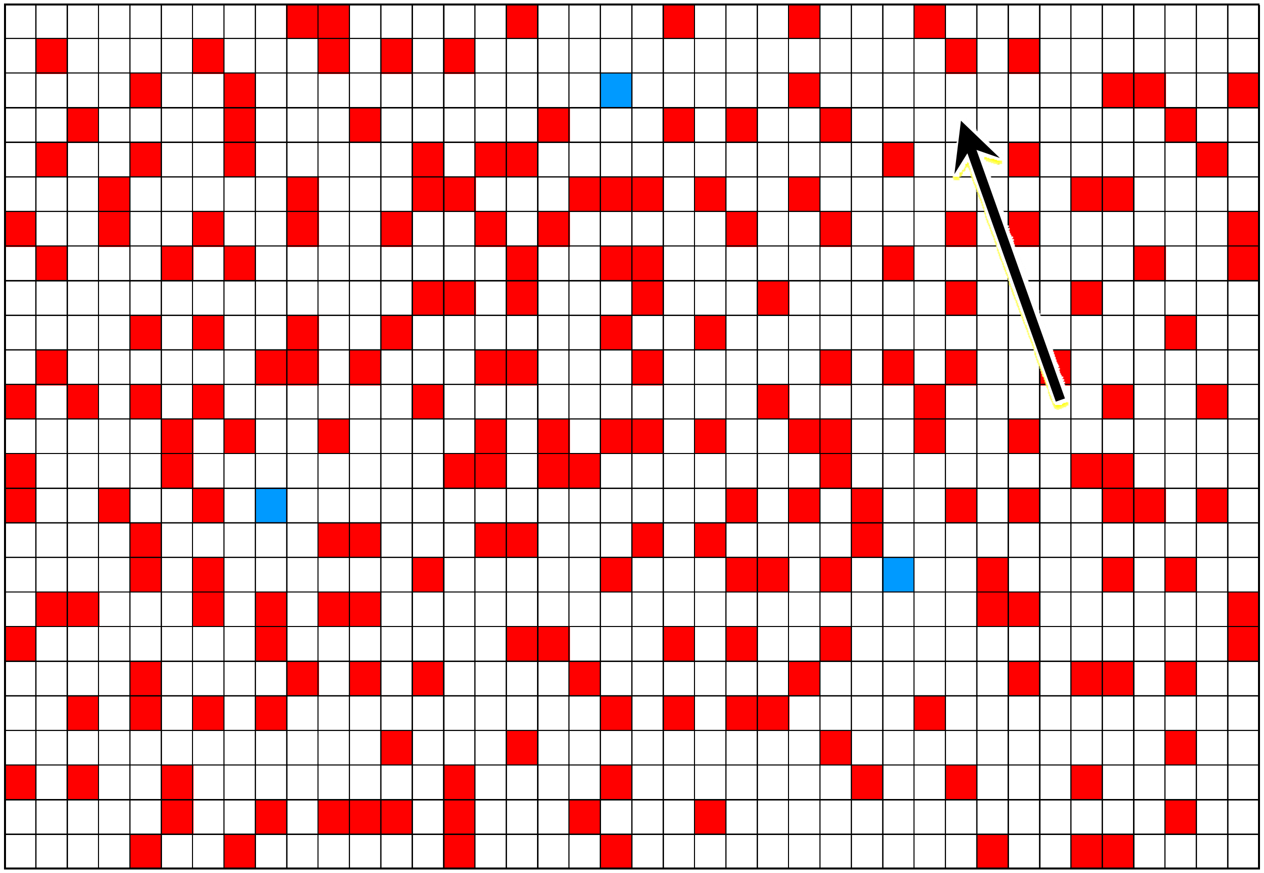

SURVEY 1

Preview to Question 7 (continued)

Suppose that the company has asked you to deal out the 10 medical products and safety inventions between yourself and one other person. You are allowed to deal out the products and inventions in any way you choose. You can select any of the following choices:

Number given to:	
**You**	**Other Person**
All 10	None
9	1
8	2
7	3
6	4
5	5
4	6
3	7
2	8
1	9
None	All 10

In Question 7, you will be told the age range of the other person (for example, age 60-69), and their relationship to you (for example, "an acquaintance"). You will be asked ten versions of Question 7 (labeled 7ɸм-7ɸмл), with each version varying the age of the other person and/or the relationship of the other person to you. In each question, you will be asked to select the number of medical products and safety inventions (between 0 and 10) that you would like to give to yourself, with the remainder going to the other person.

Preview to Question 7 (continued)

You will be shown the "baseline chance of dying within the next 10 years" for yourself and the other person. These figures are based on the mid-point of the age range. For example, when asked about someone aged 60-69, you will be shown his or her baseline chance of death during the next 10 years as 219 out of 1,000, which corresponds to the risk of death for a typical 65 year old. However, when asked about someone less than 20 years old, you will be shown their baseline chance of death during the next 10 years as 11 out of 1,000 (even though the typical youth has a slightly lower baseline chance of death).

Likewise, the questions will list a chance of death during the next 10 years for you based on the mid-point of your age range. For example, if you are in your twenties, you will be shown the baseline chance of death during the next 10 years for a typical 25 year old. We would like to stress that this baseline risk of death is simply used for illustration purposes and may not correspond well with your actual risk of death in the next 10 years. A person's risk of death is a function of the person's age, gender, health status, and many other factors.

For each of these questions, you must enter a number between 0-10 in a blue box. The number you enter will be the number of medical products and safety inventions that go to you. After you do this, you will be shown the number of medical products and safety inventions that go to the other person, and will be shown how this donation of products and inventions has changed your chance of death during the next 10 years and how this donation has changed the other person's chance of death during the next 10 years.

Assume that the other person will not know that you have been asked to make this choice to deal out the products. That is, your choice is unknown to the other person.

Before we get to Question 7, the next few questions are included to make sure you understand the concepts related to the chance of death within the next 10 years.

SURVEY 1

<u>Preview to Question 7</u>. Warm-Up Question A: Which person who has the lower chance of dying in the next 10 years? Click on the box of the person who has the lower chance of dying in the next 10 years.

	**Person 1:**		**Person 2:**
**Click on the box to select:**	☐	***OR***	☐
Chance of dying within the next 10 years:	75 in 1,000 Chance		38 in 1,000 Chance
	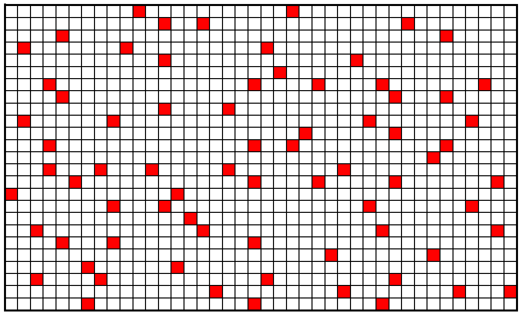		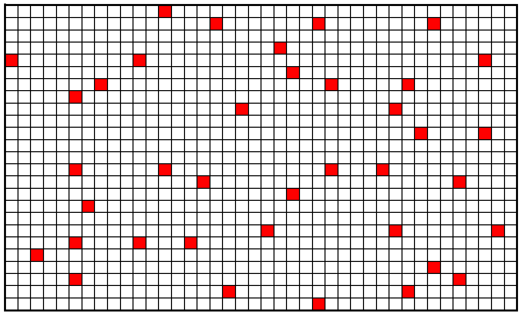
Add the following cell if they click on this person	**Not Correct. Person 1 has a *higher* chance of dying in the next 10 years than person 2.**		**Correct! Person 2 has a *lower* chance of dying in the next 10 years than person 1.**

SURVEY 1

<u>Preview to Question 7</u>. Warm-Up Question A (Try Again): Which person who has the lower chance of dying in the next 10 years? Click on the box of the person who has the lower chance of dying in the next 10 years.

	Person 1:		Person 2:
**Click on the box to select:**	☐	***OR***	☐
Chance of dying within the next 10 years:	12 in 1,000 Chance		21 in 1,000 Chance

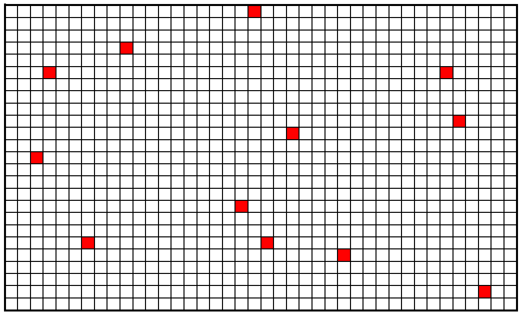

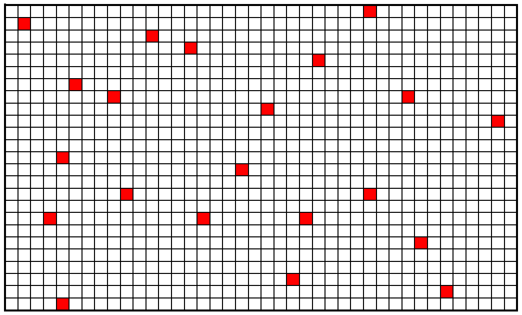

Add the following cell if they click on this person	**Correct! Person 1 has a *lower* chance of dying in the next 10 years than person 2.**	**Not Correct. Person 2 has a *higher* chance of dying in the next 10 years than person 1.**

SURVEY 1

<u>Preview to Question 7</u>. Warm-Up Question B: Which person would you rather be (assuming that you want to live for 10 years)? Click on the box of the person you would rather be.

	Person 1:		Person 2:
Click on the box to select:	☐	*OR*	☐
Chance of dying within the next 10 years:	15 in 1,000 Chance		53 in 1,000 Chance

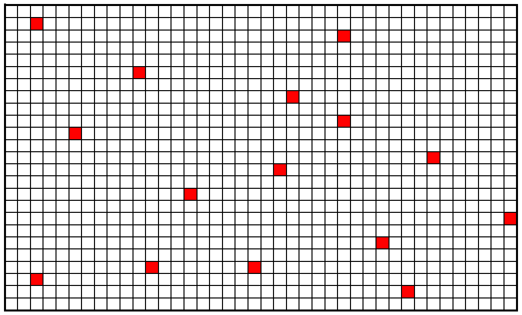

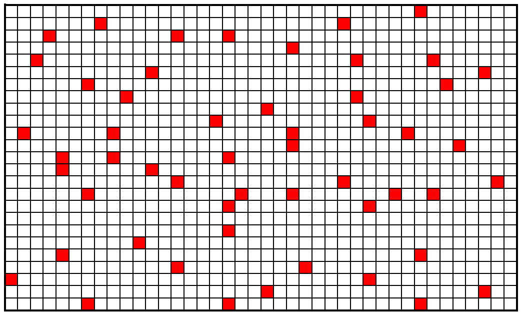

Add the following cell if they click on this person	Correct! Person 1 has a *lower* chance of dying in the next 10 years than person 2.	Not Correct. Person 2 has a *higher* chance of dying in the next 10 years than person 1.

SURVEY 1

<u>Preview to Question 7</u>. Warm-Up Question B (Try Again): Which person would you rather be (assuming that you want to live for 10 years)?
Click on the box of the person you would rather be.

	**Person 1:**		**Person 2:**
**Click on the box to select:**	☐	***OR***	☐
Chance of dying within the next 10 years:	153 in 1,000 Chance		934 in 1,000 Chance
	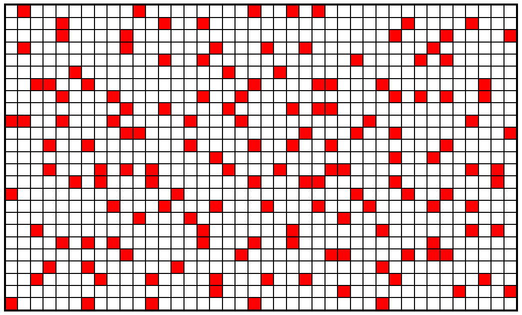		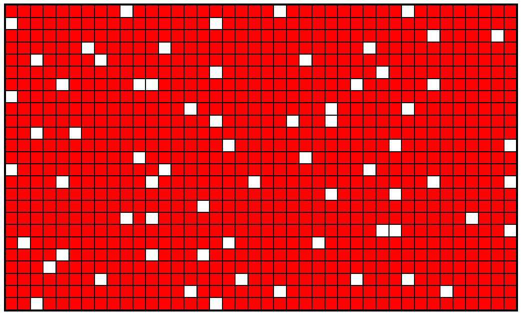
Add the following cell if they click on this person	**Correct! Person 1 has a *lower* chance of dying in the next 10 years than person 2.**		**Not Correct. Person 2 has a *higher* chance of dying in the next 10 years than person 1.**

SURVEY 1

Preview to Question 7. Warm-Up Question C: Which person has the biggest reduction in the their chance of death during the next 10 years as a result of the company's donation of medical products or safety inventions? Click on the box of the person who has the biggest reduction in the their chance of death.

	**Person 1:**		**Person 2:**
**Click on the box to select:**	☐	***OR***	☐
Company offers this number of medical products / safety inventions:	7		3
Baseline chance of dying within the next 10 years:	490 in 1,000 Chance		22 in 1,000 Chance
Chance of dying within the next 10 years ***AFTER*** medical products / safety inventions are given. (Red Squares):	483 in 1,000 Chance		19 in 1,000 Chance
	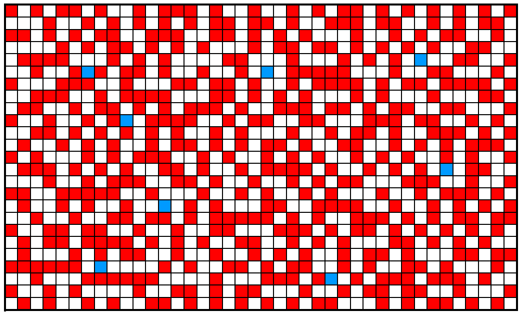		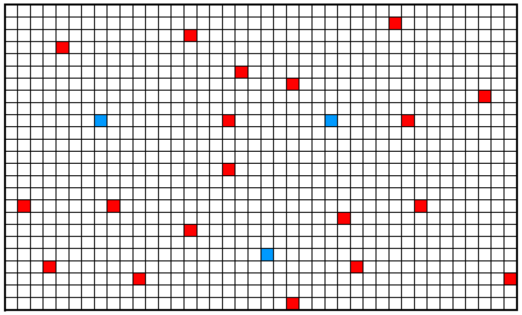
Add the following cell if they click on this person	**Correct! The reduction in the number of chances of dying during the next 10 years is greater for person 1 than person 2.**		**Not Correct. The reduction in the number of chances of dying during the next 10 years is smaller for person 2 than person 1.**

SURVEY 1

<u>Preview to Question 7</u>. Warm-Up Question C (Try Again): Which person has the biggest reduction in the their chance of death during the next 10 years as a result of the company's donation of medical products or safety inventions? Click on the box of the person who has the biggest reduction in the their chance of death.

	Person 1:		Person 2:
**Click on the box to select:**	☐	***OR***	☐
Company offers this number of medical products / safety inventions:	8		2
Baseline chance of dying within the next 10 years:	48 in 1,000 Chance		78 in 1,000 Chance
Chance of dying within the next 10 years ***AFTER*** medical products / safety inventions are given. (Red Squares):	40 in 1,000 Chance		76 in 1,000 Chance

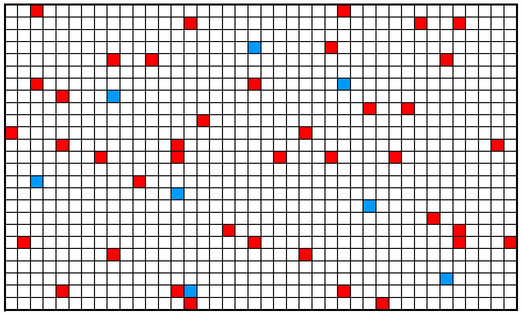

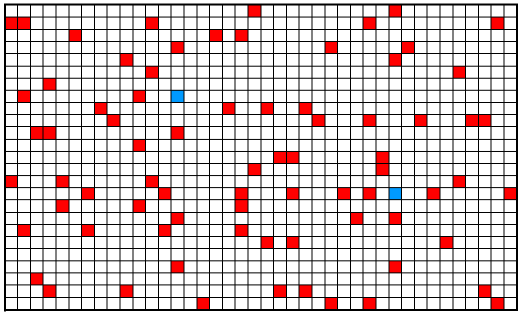

Add the following cell if they click on this person

Correct! The reduction in the number of chances of dying during the next 10 years is greater for person 1 than person 2.

Not Correct. The reduction in the number of chances of dying during the next 10 years is smaller for person 2 than person 1.

SURVEY 1

Question 7.1:
Out of the 10 medical products and safety inventions that the company gives out, how many would you like the company to give to you and how many would you like the company to give to the other person? Enter the number (between 0 and 10) in the blue box that you would like to be given to you. After you enter this number, the remainder (10 minus the number given to you) will be shown in the orange box, and the number of products/inventions given to you and the other person will be shown as blue squares in the grid below. You may change your entry in the blue box if you are not satisfied. Once you are satisfied with your choice, click the "Continue" button.

	**You**<br>**age 30-39**	**Other person is**<br>**an acquaintance**<br>**age 40-49**
**In the blue box, enter the number of medical products or safety inventions you would like to be given to you**		
Baseline chance of dying within the next 10 years:	22 in 1,000 Chance	48 in 1,000 Chance

Continue

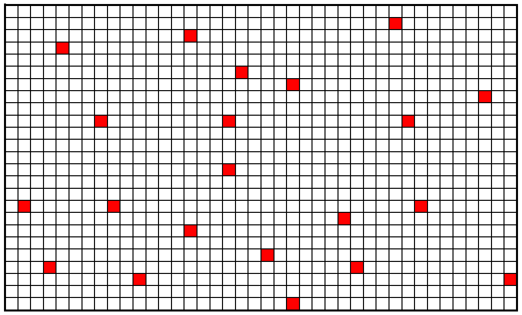
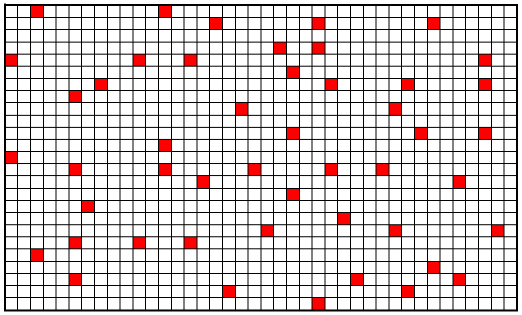

SURVEY 1

Question 7.1:
Out of the 10 medical products and safety inventions that the company gives out, how many would you like the company to give to you and how many would you like the company to give to the other person? Enter the number (between 0 and 10) in the blue box that you would like to be given to you. After you enter this number, the remainder (10 minus the number given to you) will be shown in the orange box, and the number of products/inventions given to you and the other person will be shown as blue squares in the grid below. You may change your entry in the blue box if you are not satisfied. Once you are satisfied with your choice, click the "Continue" button.

	**You**<br>**age 30-39**	**Other person is**<br>**an acquaintance**<br>**age 40-49**
**In the blue box, enter the number of medical products or safety inventions you would like to be given to you**	**7**	**3**
	**Continue**	
Baseline chance of dying within the next 10 years:	22 in 1,000 Chance	48 in 1,000 Chance
Chance of dying within the next 10 years ***AFTER*** medical products / safety inventions are given. (Red Squares):	15 in 1,000 Chance	45 in 1,000 Chance

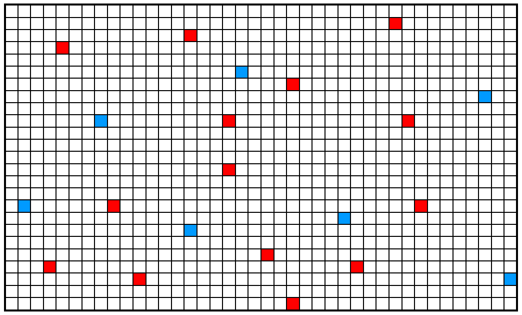

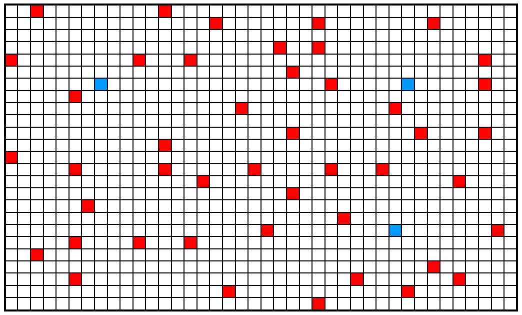

Preview to Question 8: Chance of Winning $25,000

Question 8 is about the chance of you or others winning money. Imagine that a company decided to give out ten scratch-off tickets and these ten tickets will be given to you and/or one other person. Once they are given out, assume that they cannot be given or sold to someone else. Each ticket has one chance in 1,000 of winning $25,000 from the company.

A person with one ticket has 1 chance in 1,000 to win $25,000. This relationship can be seen in this figure.

Chance of having winning $25,000 = **1** out of 1,000.

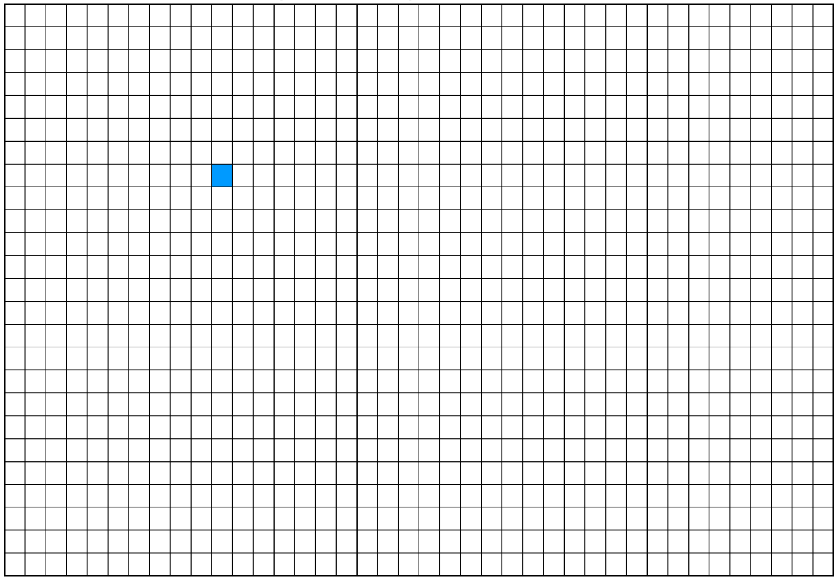

There are 1,000 squares shown. You can think of each square like the slot on a roulette wheel, where if by chance the roulette ball landed in a blue square, the person will win $25,000.

SURVEY 1

<u>Preview to Question 8 (continued)</u>

Imagine a roulette ball randomly landing in one of the squares on the grid. If the ball lands in a white square, the person will receive nothing.

Chance of Winning $25,000 = **1** out of 1,000.

(Result: Does Not Win $25,000)

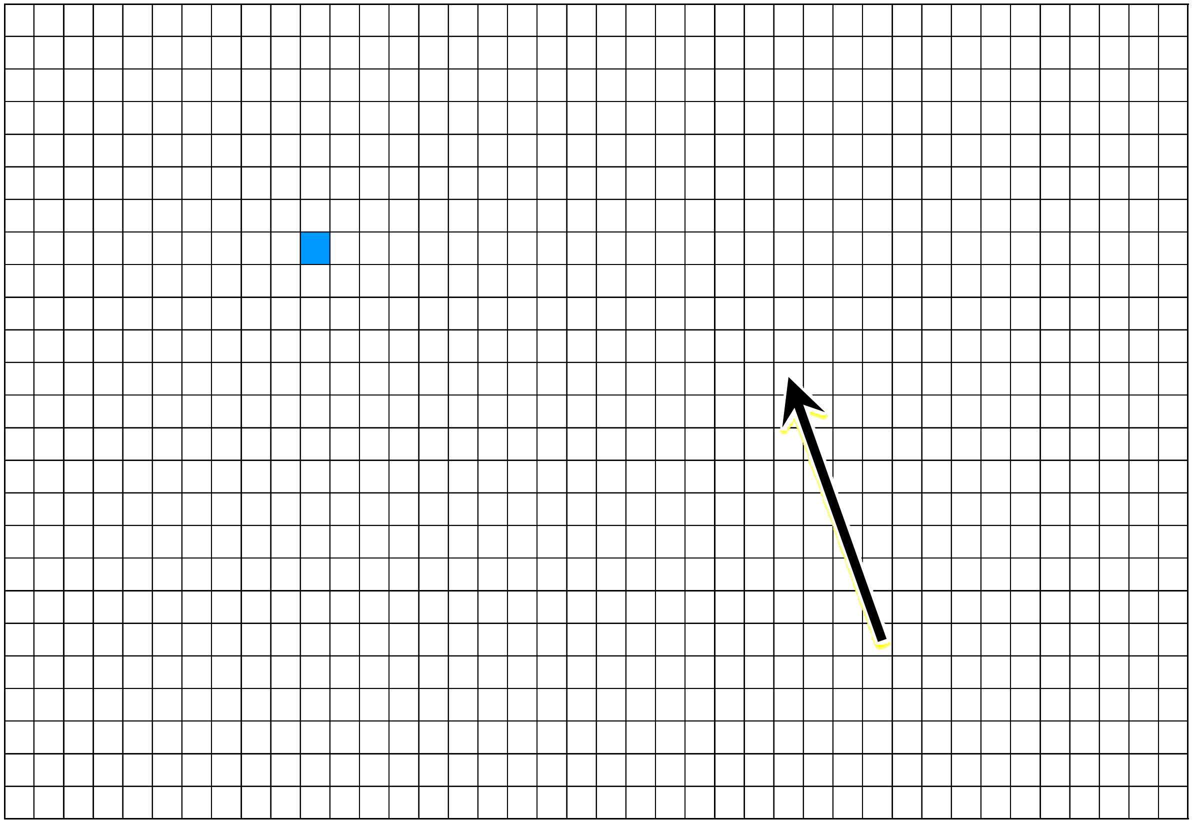

Preview to Question 8 (continued)

If the ball lands in a blue square the person will receive $25,000. Out of 1,000 spins of the roulette wheel, we would expect it to land on a blue square 1 time.

Chance of Winning $25,000 = **1** out of 1,000.

(Result: Wins $25,000)

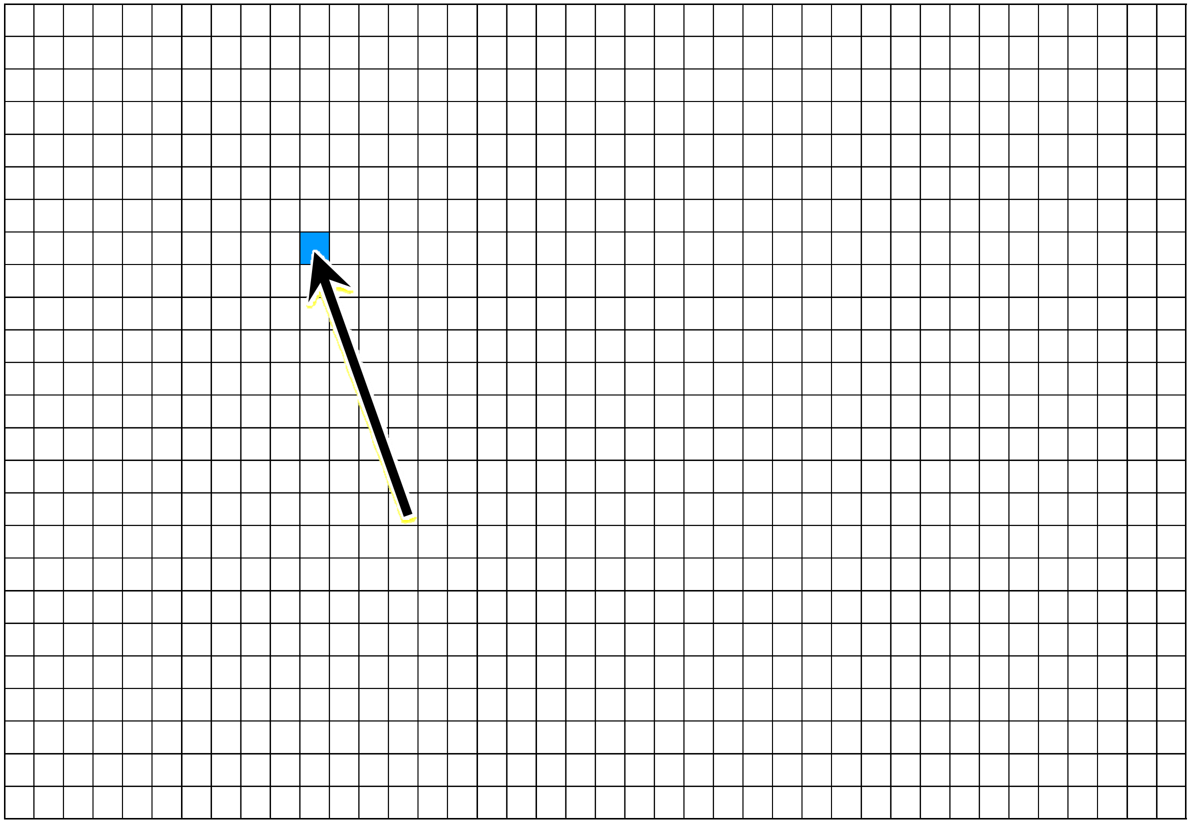

Preview to Question 8 (continued)

If the person receives 7 tickets, the person's chance of winning money would be 7 chances in 1,000. This relationship can be seen in this figure.

Chance of having a winning ticket = **7** out of 1,000.

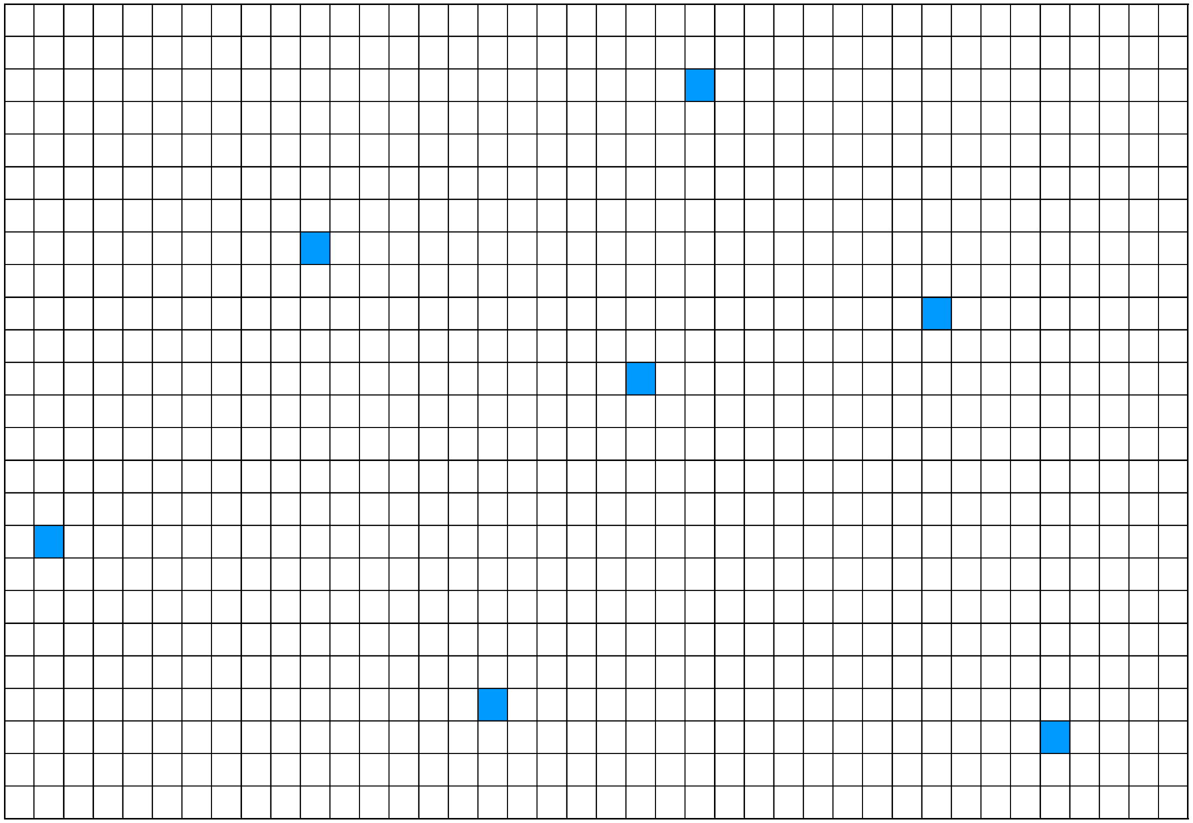

Preview to Question 8 (continued)

Suppose that the company has asked you to deal out the 10 scratch-off tickets between yourself and one other person. You are allowed to deal out the tickets in any way you choose. You can select any of the following choices:

Number given to:	
**You**	**Other Person**
All 10	None
9	1
8	2
7	3
6	4
5	5
4	6
3	7
2	8
1	9
None	All 10

In Question 8, you will be told the age range of the other person (for example, age 60-69), and their relationship to you (for example, “an acquaintance”). You will be asked ten versions of Question 8 (labeled 8ɸм-8ɸмл), with each version varying the age of the other person and/or the relationship of the other person to you. In each question, you will be asked to select the number of tickets (between 0 and 10) that you would like to give to yourself, with the remainder going to the other person.

Preview to Question 8 (continued)

For each of these questions, you must enter a number between 0-10 in a blue box. The number you enter will be the number of tickets that go to you. After you do this, you will be shown the number of tickets that go to the other person, and will be shown how this donation of tickets has changed your chance of winning money and how this donation has changed the other person's chance of winning money.

Assume that the other person will not know that you have been asked to make this choice to deal out the tickets. That is, your choice is unknown to the other person.

Before we get to Question 8, the next few questions are included to make sure you understand the concepts related to the chance of winning money from the company.

SURVEY 1

Preview to Question 8. **Warm-Up Question A: Which person has the higher chance of having a winning ticket?** Click on the box of the person who has the higher chance of having a winning ticket.

	**Person 1:**		**Person 2:**
**Click on the box to select:**	☐	***OR***	☐
Person receives this number of scratch-off tickets:	7		3
Baseline chance of winning $25,000 from the company:	0 in 1,000 Chance		0 in 1,000 Chance
Chance of winning $25,000 from the company ***AFTER*** tickets are given. (Blue Squares):	7 in 1,000 Chance		3 in 1,000 Chance
	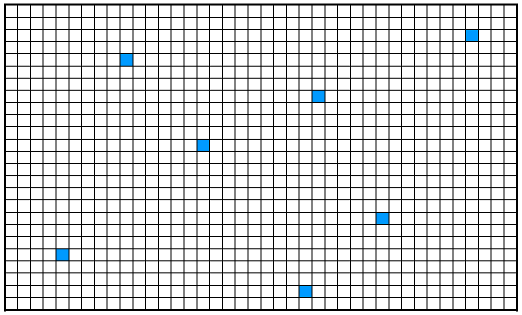		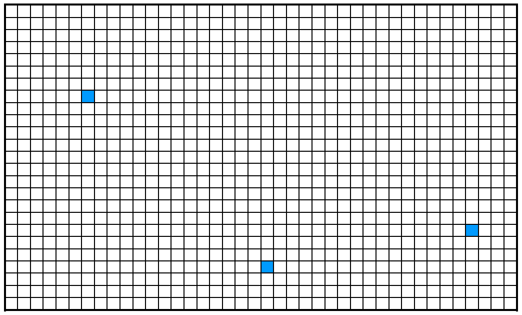
Add the following cell if they click on this person	**Correct! Person 1 has a *higher* chance of having a winning ticket than person 2.**		**Not Correct. Person 2 has a *lower* chance of having a winning ticket than person 1.**

SURVEY 1

<u>Preview to Question 8</u>. Warm-Up Question A (Try Again): Which person has the higher chance of having a winning ticket? Click on the box of the person who has the higher chance of having a winning ticket.

	Person 1:		Person 2:
**Click on the box to select:**	☐	***OR***	☐
Person receives this number of scratch-off tickets:	2		8
Baseline chance of winning $25,000 from the company:	0 in 1,000 Chance		0 in 1,000 Chance
Chance of winning $25,000 from the company ***AFTER*** tickets are given. (Blue Squares):	2 in 1,000 Chance		8 in 1,000 Chance
	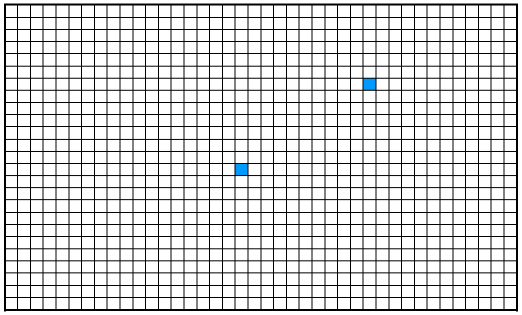		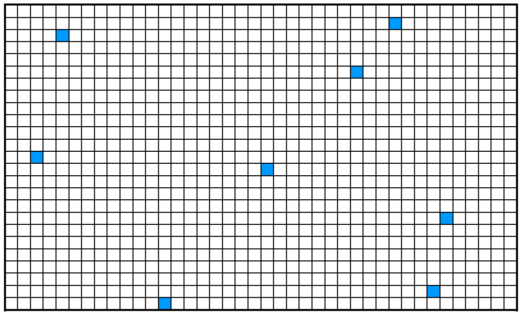
Add the following cell if they click on this person	**Not Correct. Person 1 has a *lower* chance of having a winning ticket than person 2.**		**Correct! Person 2 has a *higher* chance of having a winning ticket than person 1.**

SURVEY 1

<u>Preview to Question 8</u>. Warm-Up Question B: Which person would you rather be (assuming that you want to win $25,000 from the company)? Click on the box of the person you would rather be.

	Person 1:		Person 2:
**Click on the box to select:**	☐	***OR***	☐
Person receives this number of scratch-off tickets:	4		6
Baseline chance of winning $25,000 from the company:	0 in 1,000 Chance		0 in 1,000 Chance
Chance of winning $25,000 from the company ***AFTER*** tickets are given. (Blue Squares):	4 in 1,000 Chance		6 in 1,000 Chance
	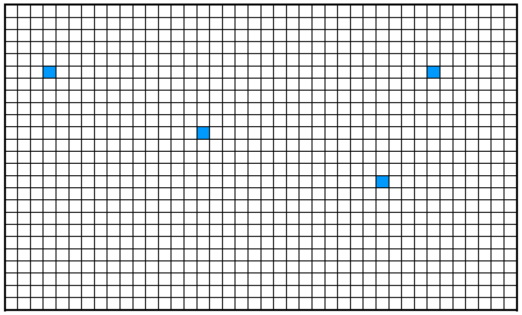		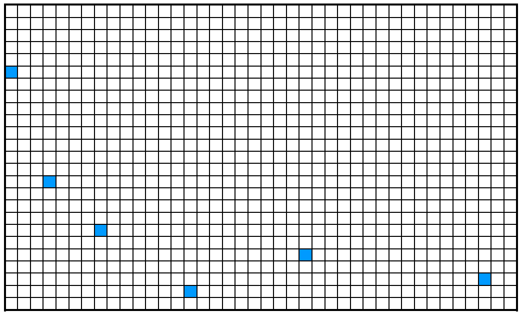
Add the following cell if they click on this person	**Not Correct. Person 1 has a *lower* chance of having a winning ticket than person 2.**		**Correct! Person 2 has a *higher* chance of having a winning ticket than person 1.**

SURVEY 1

Preview to Question 8. Warm-Up Question B (Try Again): Which person would you rather be (assuming that you want to win $25,000 from the company)? Click on the box of the person you would rather be.

	Person 1:		Person 2:
**Click on the box to select:**	☐	***OR***	☐
Person receives this number of scratch-off tickets:	2		8
Baseline chance of winning $25,000 from the company:	0 in 1,000 Chance		0 in 1,000 Chance
Chance of winning $25,000 from the company ***AFTER*** tickets are given. (Blue Squares):	2 in 1,000 Chance		8 in 1,000 Chance
	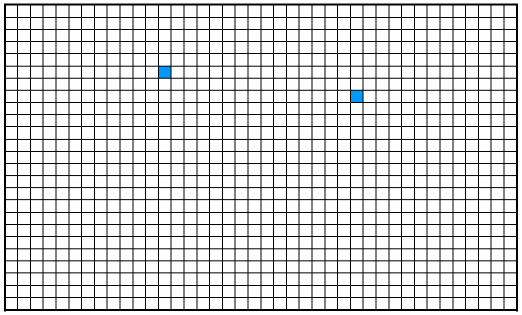		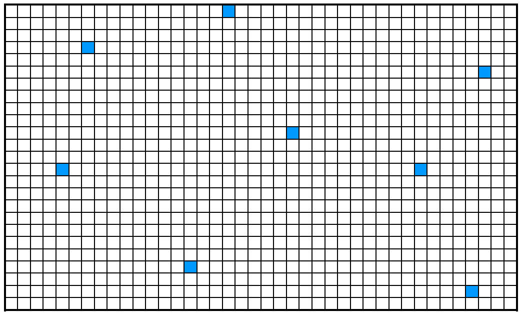
Add the following cell if they click on this person	**Not Correct. Person 1 has a *lower* chance of having a winning ticket than person 2.**		**Correct! Person 2 has a *higher* chance of having a winning ticket than person 1.**

SURVEY 1

Question 8.1:
Out of the 10 scratch-off tickets that the company gives out, how many would you like the company to give to you and how many would you like the company to give to the other person? Enter the number (between 0 and 10) in the blue box that you would like to be given to you. After you enter this number, the remainder (10 minus the number given to you) will be shown in the orange box, and the number of tickets given to you and the other person will be shown as blue squares in the grid below. You may change your entry in the blue box if you are not satisfied. Once you are satisfied with your choice, click the "Continue" button.

	**You** **age 30-39**	**Other person is** **an acquaintance** **age 40-49**
**In the blue box, enter the number of tickets you would like to go to you**		
Baseline chance of winning $25,000 from the company:	0 in 1,000 Chance	0 in 1,000 Chance

Continue

SURVEY 1

Question 8.1:
Out of the 10 scratch-off tickets that the company gives out, how many would you like the company to give to you and how many would you like the company to give to the other person? Enter the number (between 0 and 10) in the blue box that you would like to be given to you. After you enter this number, the remainder (10 minus the number given to you) will be shown in the orange box, and the number of tickets given to you and the other person will be shown as blue squares in the grid below. You may change your entry in the blue box if you are not satisfied. Once you are satisfied with your choice, click the "Continue" button.

	**You**<br>**age 30-39**	**Other person is**<br>**an acquaintance**<br>**age 40-49**
**In the blue box, enter the number of tickets you would like to go to you**	**7**	**3**
	**Continue**	
Baseline chance of winning $25,000 from the company:	0 in 1,000 Chance	0 in 1,000 Chance
Chance of winning $25,000 from the company ***AFTER*** tickets are given. (Blue Squares):	7 in 1,000 Chance	3 in 1,000 Chance

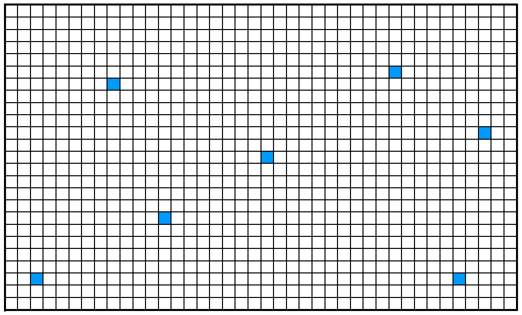

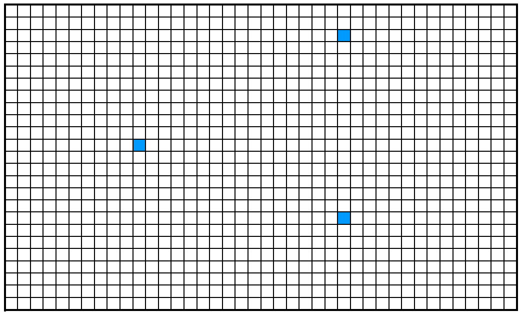

SURVEY 1

Preview to Question 9.

Question 9 is about how much you would be willing to pay for medical products and/or safety inventions that would lower your risk of dying during the next 10 years. Assume that you will use the medical products and/or safety inventions for 10 years. The amount you pay will be split into ten annual payments. For example, if you are willing to pay $1,000 in total, your annual payment will be $100 for each of 10 years.

SURVEY 1

Question 9a: Would you be willing to pay $L *in total* for medical products and/or safety inventions that would lower your risk of dying during the next 10 years by M chances in 1,000? (Your annual payment would be $L/10).

Yes ☐ No ☐

SURVEY 1

Question 9b: Would you be willing to pay $L *in total* for medical products and/or safety inventions that would lower your risk of dying during the next 10 years by M chances in 1,000? (Your annual payment would be $L/10).

Yes ☐ No ☐

SURVEY 1

Question 9c: What is the most amount of money you be willing to pay *in total* for medical products and/or safety inventions that would lower your risk of dying during the next 10 years by M chances in 1,000?

$____________ (Your annual payment would be this amount divided by 10)

OR

Question 9c: Would you be willing to pay anything at all for medical products and/or safety inventions that would lower your risk of dying during the next 10 years by M chances in 1,000?

Yes ☐ No ☐

SURVEY 1

Question 9d: What is the most amount of money you be willing to pay *in total* for medical products and/or safety inventions that would lower your risk of dying during the next 10 years by M chances in 1,000?

$____________ (Your annual payment would be this amount divided by 10)

SURVEY 1

Question 10: How would you rate your undertanding of probability?
(Click one box)

- **(A)** I do not understand probability at all
- **(B)** I have a poor understanding of probability
- **(C)** I have a fair understanding of probability
- **(D)** I have a good understanding of probability
- **(E)** I have an excellent understanding of probability

SURVEY 1

Question 11: What country or region were you born in? (Click one box)

North America

- United States of America ☐
- Canada ☐
- Mexico ☐
- El Salvador ☐
- Guatemala ☐
- Cuba ☐
- Dominican Republic ☐
- Other Country in North America (including Central America) ☐

Asia

- China ☐
- India ☐
- Phillipines ☐
- Vietnam ☐
- Other Country in Asia ☐

- Europe ☐
- South America ☐
- Africa ☐
- Australia or New Zealand ☐
- Other Pacific Island ☐
- Other Region ☐

SURVEY 1

Question 12: What state were you born in? (Click one box)

Alabama	☐	Massachusetts	☐	South Dakota	☐
Arizona	☐	Michigan	☐	Tennessee	☐
Arkansas	☐	Minnesota	☐	Texas	☐
California	☐	Mississippi	☐	Utah	☐
Colorado	☐	Missouri	☐	Vermont	☐
Connecticut	☐	Montana	☐	Virginia	☐
Delaware	☐	Nebraska	☐	Washington	☐
District of Columbia	☐	Nevada	☐	West Virginia	☐
Florida	☐	New Hampshire	☐	Wisconsin	☐
Georgia	☐	New Jersey	☐	Wyoming	☐
Hawaii	☐	New Mexico	☐	American Samoa, Guam, Puerto Rico, Virgin Islands, Other	☐
Idaho	☐	New York	☐		
Illinois	☐	North Carolina	☐		
Indiana	☐	North Dakota	☐		
Iowa	☐	Ohio	☐		
Kansas	☐	Oklahoma	☐		
Kentucky	☐	Oregon	☐		
Louisiana	☐	Pennsylvania	☐		
Maine	☐	Rhode Island	☐		
Maryland	☐	South Carolina	☐		
