# Supplementary material for: Altruism by age and social proximity
Source: PLoS One. 2017 Aug 24;12(8):e0180411. doi: 10.1371/journal.pone.0180411 (PMC5570493; doi:10.1371/journal.pone.0180411)
Supplement: S2 File — (PDF) [file pone.0180411.s002.pdf]

SURVEY 2

Question 1: What is your age? Click on the box next to your age.

- Age 18-29 ☐
- Age 30-39 ☐
- Age 40-49 ☐
- Age 50-59 ☐
- Age 60-69 ☐
- Age 70-79 ☐
- Age 80 or older ☐

SURVEY 2

Question 2: How many living immediate family members do you have in each age group? If you do not know the age of the family member, please give your best guess. If you do not have any immediate family members who are in this age group, please leave the cell blank. The sum of your entries should match the total number of living immediate family members you have of each type.

	Enter the *number* of family members of each type in each cell										
**Type of immediate family member**	**Age 0-4**	**Age 5-9**	**Age 10-17**	**Age 18-29**	**Age 30-39**	**Age 40-49**	**Age 50-59**	**Age 60-69**	**Age 70-79**	**Age 80 or older**	**Sum of your entries**
Your parents (including mother, father, stepmother, stepfather)											
Your siblings (including brothers, sisters, step-siblings, half-siblings)											
Your spouse or partner (including long-term relationships)											
Your children (including biological and adopted children)											

SURVEY 2

Question 3: How many living extended family members do you have in total by type?
Enter the TOTAL NUMBER of living extended family members you have of each type. If you do not know the exact number, please give your best guess. If you do not have any family members living in this category (for example, if your grandparents are not living or you have never had children), then enter "0".

**Type of extended family member**	**Enter the *TOTAL NUMBER* of each type in each cell**
Your grandparents<br>(your parents' / stepparents' biological parents)	☐
Your aunts and uncles<br>(your parents' / stepparents' siblings and their spouses)	☐
Your cousins<br>(children of your aunts and uncles)	☐
Your siblings' spouse(s)<br>(your sisters-in-laws & brothers-in-law via your siblings)	☐
Your siblings' children<br>(your nieces and nephews)	☐
Your spouse or partner's parents<br>(your mother-in-law, father-in-law)	☐
Your spouse or partner's siblings<br>(your sisters-in-law & brothers-in-law via your spouse)	☐
Your children's spouses<br>(your daughters-in-law, sons-in-law)	☐
Your grandchildren<br>(your children's children)	☐

SURVEY 2

Question 4: Do you have ANY living extended family members in each age group? Click on the box if you have ANY living extended family members in this age group. Please give your best guess if you are not sure of extended family members' ages.

Click on the box if you believe the you have *ANY* living extended family members in this cell.										*TOTAL NUMBER* of extended family members from question 3.
Age 0-4	Age 5-9	Age 10-17	Age 18-29	Age 30-39	Age 40-49	Age 50-59	Age 60-69	Age 70-79	Age 80 or older	

Extended family include your:
- Grandparents,
- Aunts and Uncles,
- Cousins,
- Siblings' Spouse(s),
- Siblings' Children (Nephews and Nieces),
- Spouse's Parents,
- Spouse's Siblings,
- Children's Spouse(s), and
- Grandchildren

SURVEY 2

Question 5: How many close friends, co-workers, and acquaintances do you have? Enter the TOTAL NUMBER of your close friends, co-workers, and acquaintances. We understand that you are unlikely to know the exact number -- please give your best guess. If you do not have any persons in this category (for example, you have no co-workers as you are unemployed), then enter "0".

**Type of relation**	**Enter the *TOTAL NUMBER* of each type in each cell**
Close Friends<br>(Include non-family members who you have close friendship with. For example, someone you talk with regularly and have a strong friendly bond).	☐
Co-workers<br>(Include anyone you work with regularly who is not a family member or close friend.)	☐
Acquaintances<br>(Include anyone who is not a family member, close friend, or co-worker who you know well enough that you would say "hello" to if you passed them on the street.)	☐

SURVEY 2

Question 6: What are the ages of your close friends, co-workers, and acquaintences? Click on the boxes that best reflect the ages of your close friends, co-workers, and acquaintences. For example, if most of your close friends are aged 30-39 or 40-49, then you would click on both of these boxes in the "Close Friends" row. If your co-workers are equally spread between the ages 18 and 65, you would click all of the boxes in the "Co-workers" row except Aged 0-4, 5-9, 10-17, 70-79, and 80 or older.

**Type of relation**	**Age 0-4**	**Age 5-9**	**Age 10-17**	**Age 18-29**	**Age 30-39**	**Age 40-49**	**Age 50-59**	**Age 60-69**	**Age 70-79**	**Age 80 or older**	***TOTAL NUMBER* of this type from question 5.**
Close Friends (Include non-family members who you have close friendship with. For example, someone you talk with regularly and have a strong friendly bond).											
Co-workers (Include anyone you work with regularly who is not a family member or close friend.)											
Acquaintances (Include anyone who is not a family member, close friend, or co-worker who you know well enough that you would say "hello" to if you passed them on the street.)											

(The age columns are grouped under the heading: **Click on the boxes that best reflect the ages of this type of relation**)

SURVEY 2

Preview to Questions 7 and 8

The next two sets of questions will ask you to make choices about things where chance or luck plays a role in the outcome.

An example of a chance event is the flipping of a coin. If a coin were tossed 100 times, on average it would land on heads 50 times and land on tails 50 times.

Likewise, if a coin were tossed 1,000 times, on average it would land on heads 500 times and land on tails 500 times.

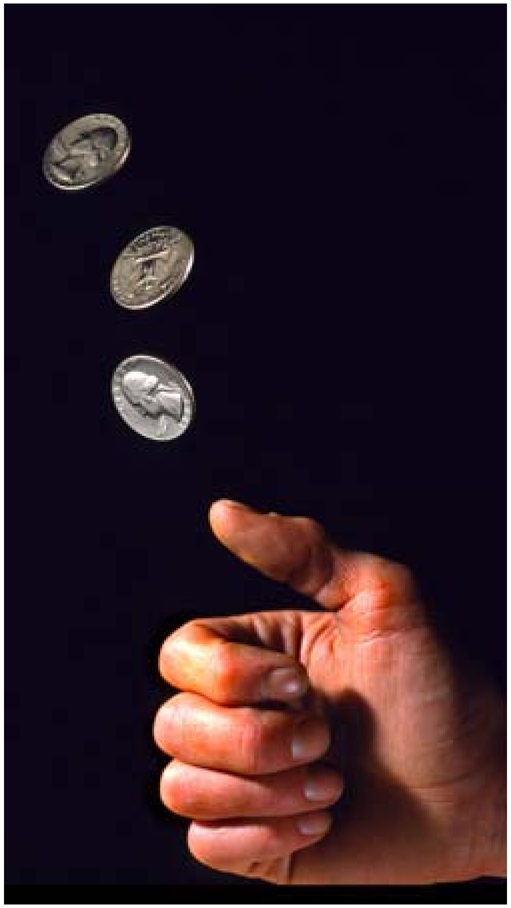

SURVEY 2

Preview to Questions 7 and 8 (Continued)

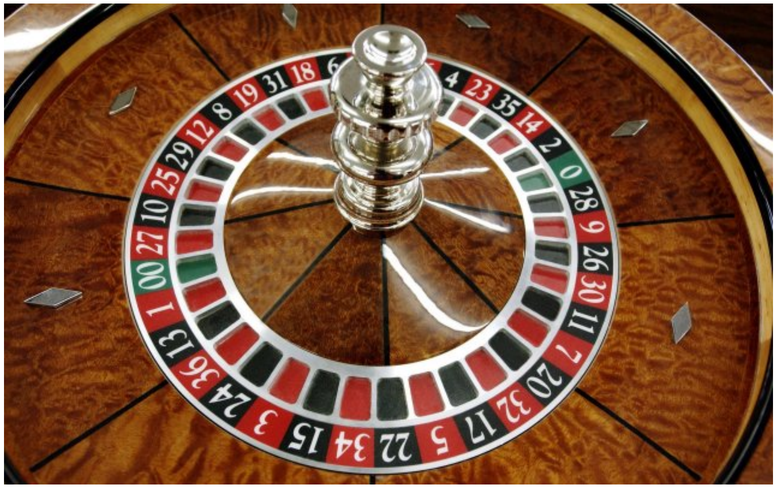

Another example is a roulette wheel.

An American roulette wheel has 38 slots.

The ball should land in any given spot (for example the #7 slot), once out of every 38 spins.

If the roulette wheel were spun 38,000 times, on average the ball will land in the #7 slot 1,000 times.

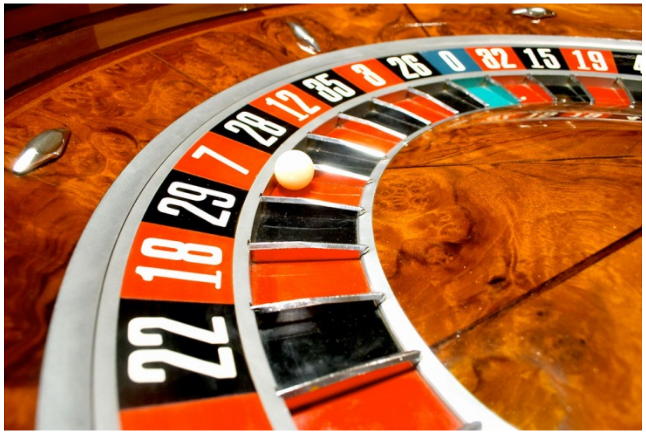

SURVEY 2

Preview to Question 7: Chance of Dying During the Next 10 Years

Question 7 will include an estimate of the chance of you or others dying during the next 10 years.

The next screens will illustrate the chance of dying within the next 10 years for people at various ages. Each screen will present a figure with 10,000 squares shown. The number of red squares is equal to the number of chances out of 10,000 that the typical person will die within 10 years. You can think of each square like the slot on a roulette wheel, where if by chance the roulette ball landed in a red square, the person would unfortunately die. As shown on the next several pages, as people age, the chance of dying during the next 10 years rises after childhood.

SURVEY 2

Age **0-4**: Chance of Dying During the Next 10 Years = **30** out of 10,000 (or 0.30%).

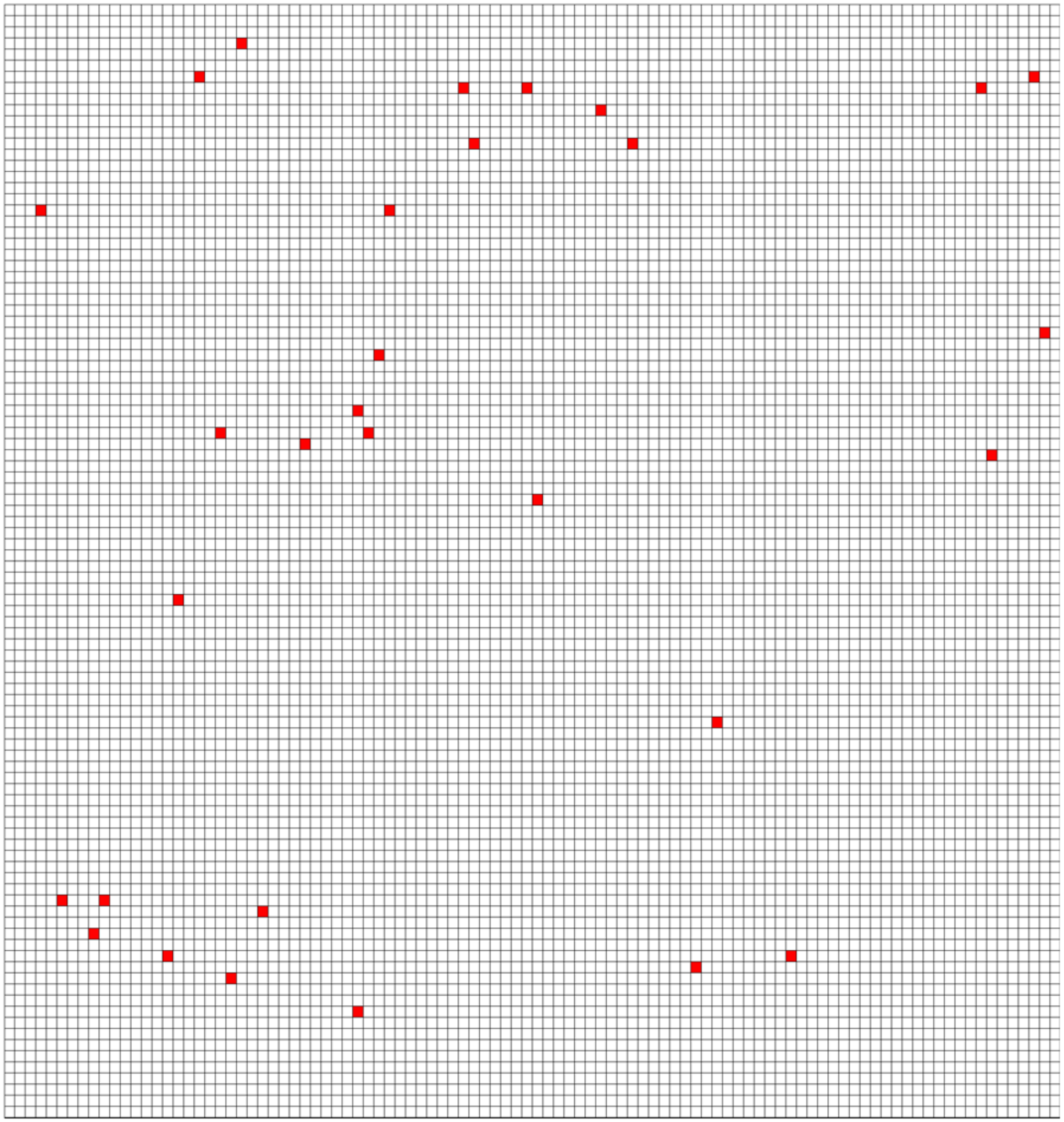

SURVEY 2

Age **5-9**: Chance of Dying During the Next 10 Years = **23** out of 10,000 (or 0.23%).

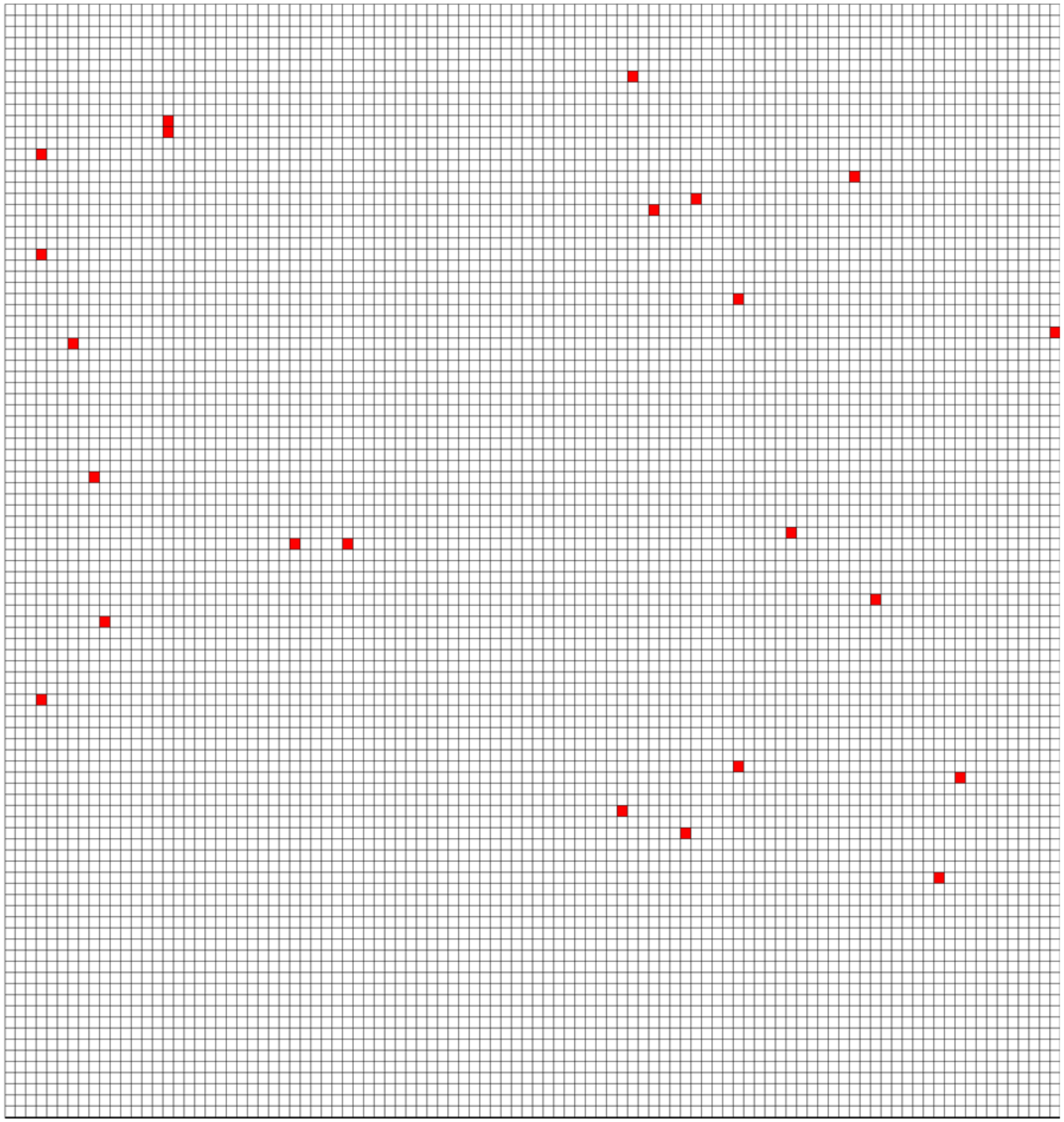

SURVEY 2

Age **10-17**: Chance of Dying During the Next 10 Years = **69** out of 10,000 (or 0.69%).

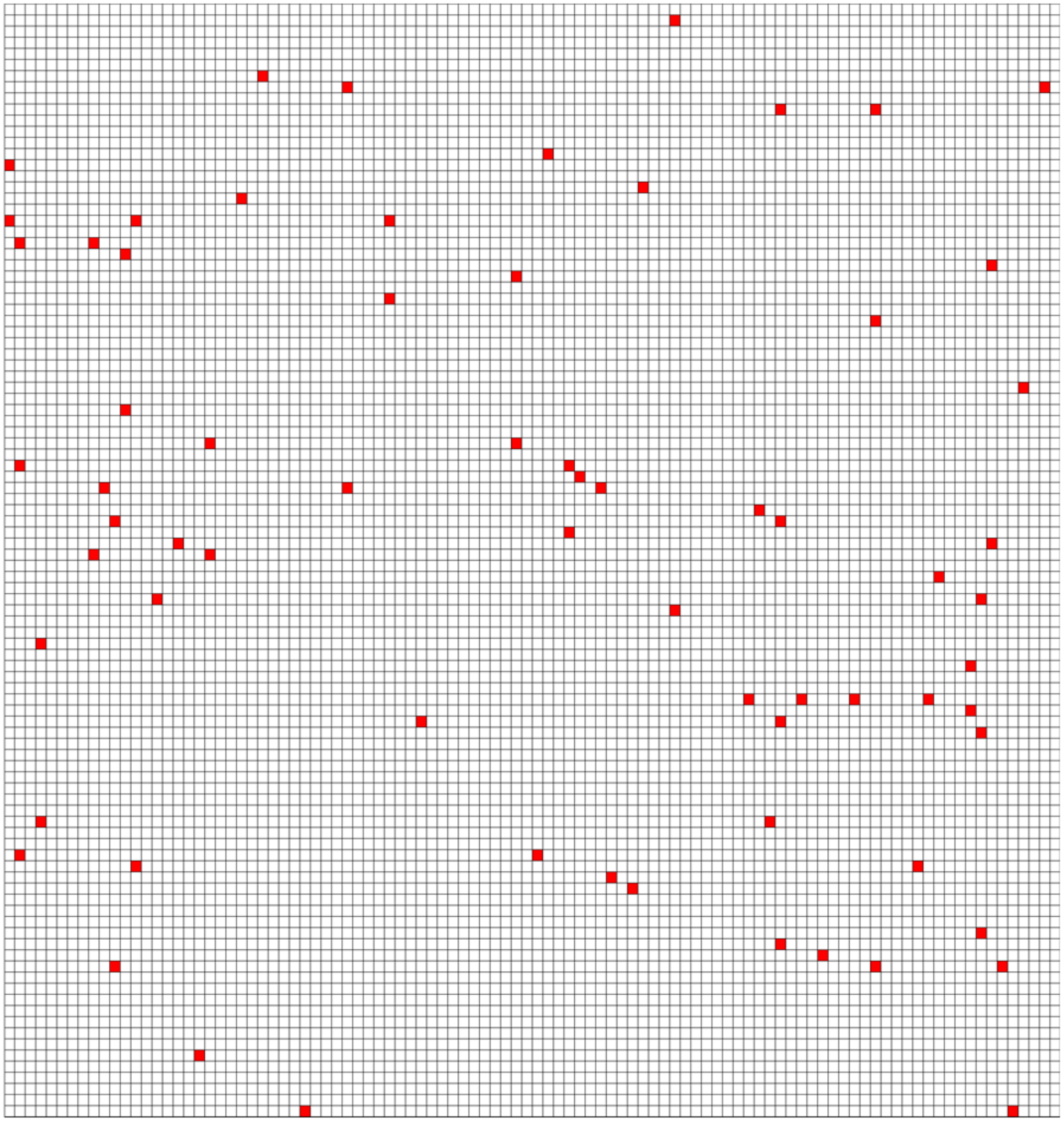

SURVEY 2

Age **18-29**: Chance of Dying During the Next 10 Years = **106** out of 10,000 (or 1.06%).

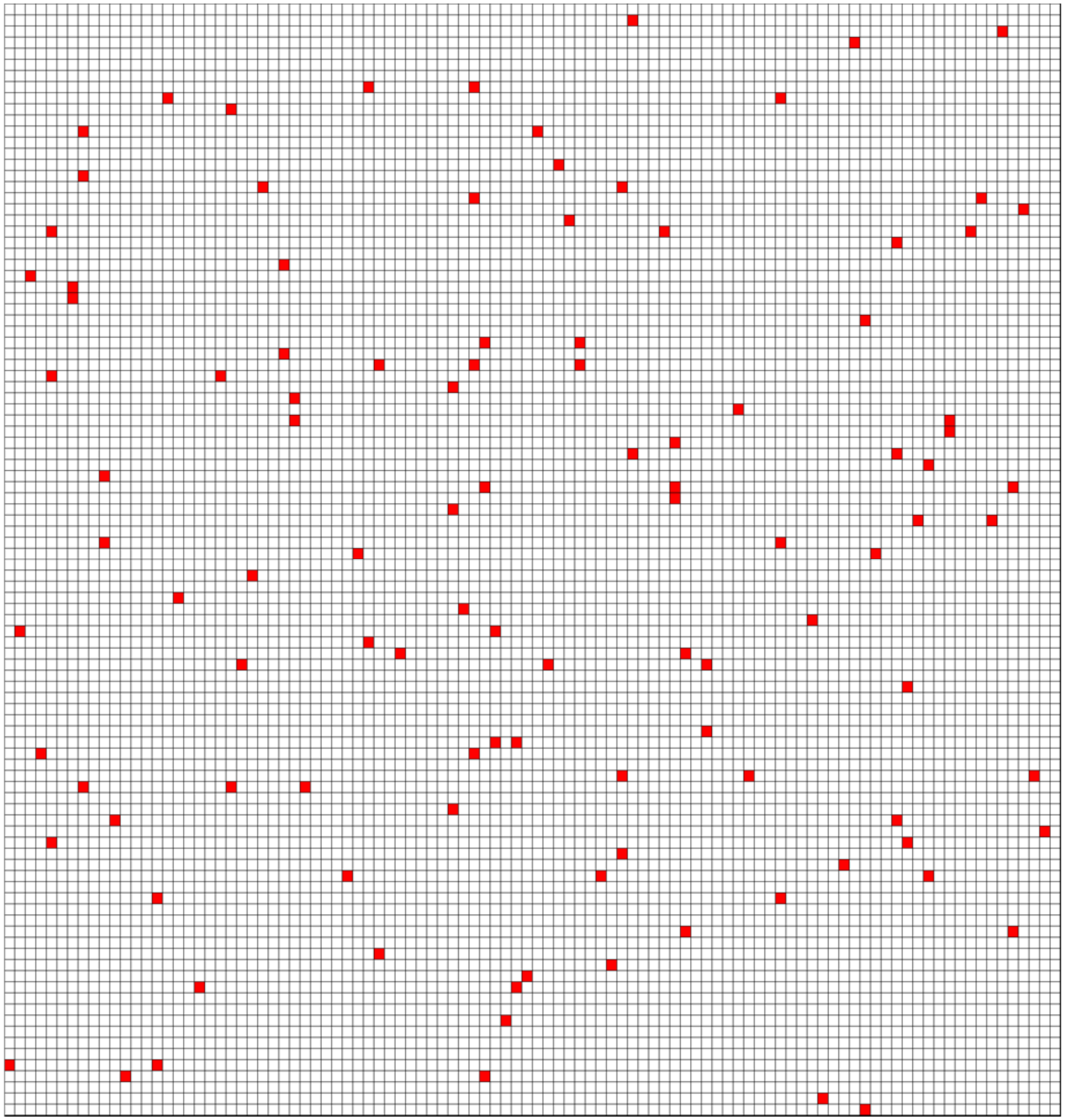

SURVEY 2

Age 30-39: Chance of Dying During the Next 10 Years = 186 out of 10,000 (or 1.86%).

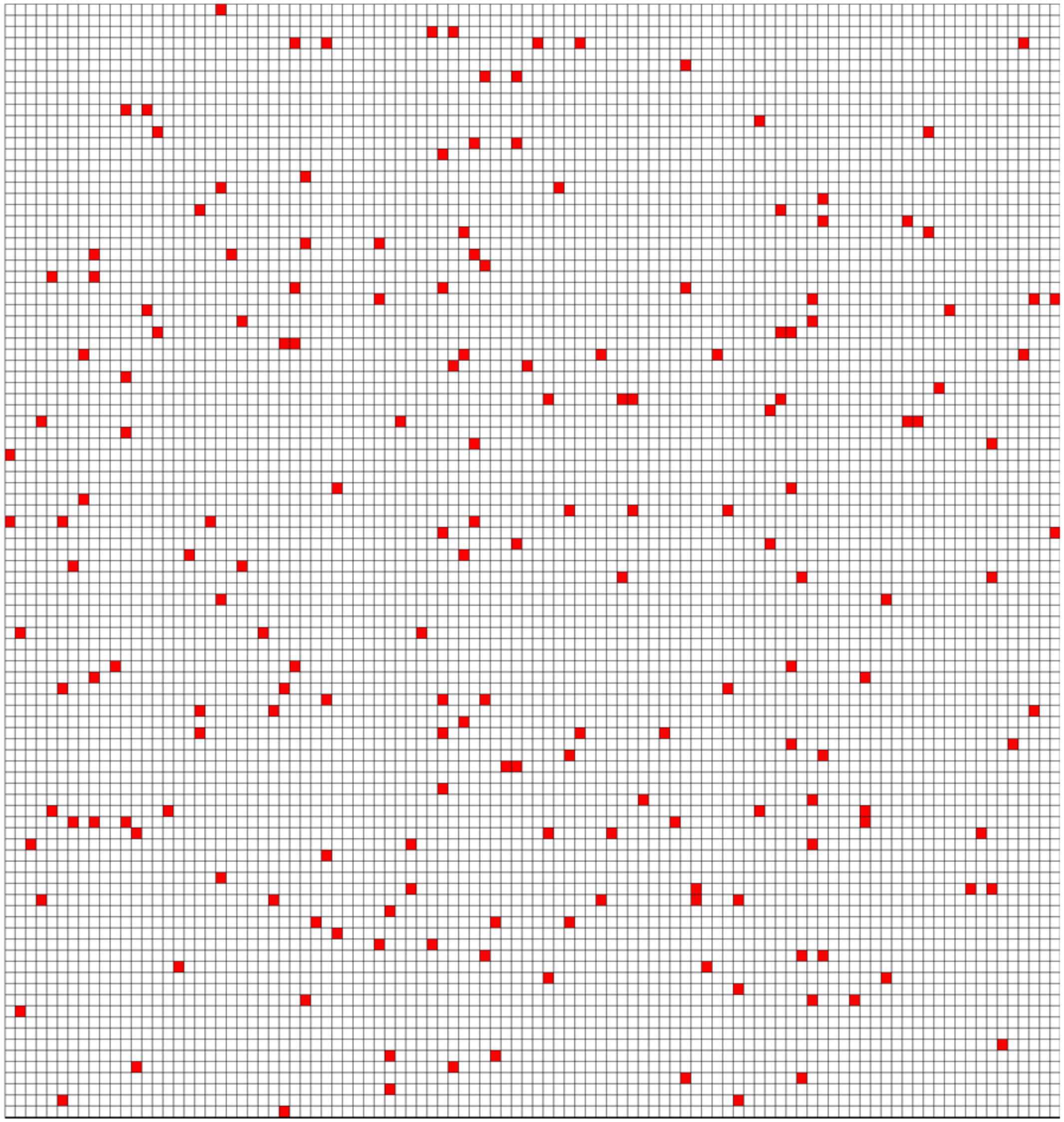

SURVEY 2

Age 40-49: Chance of Dying During the Next 10 Years = 414 out of 10,000 (or 4.14%).

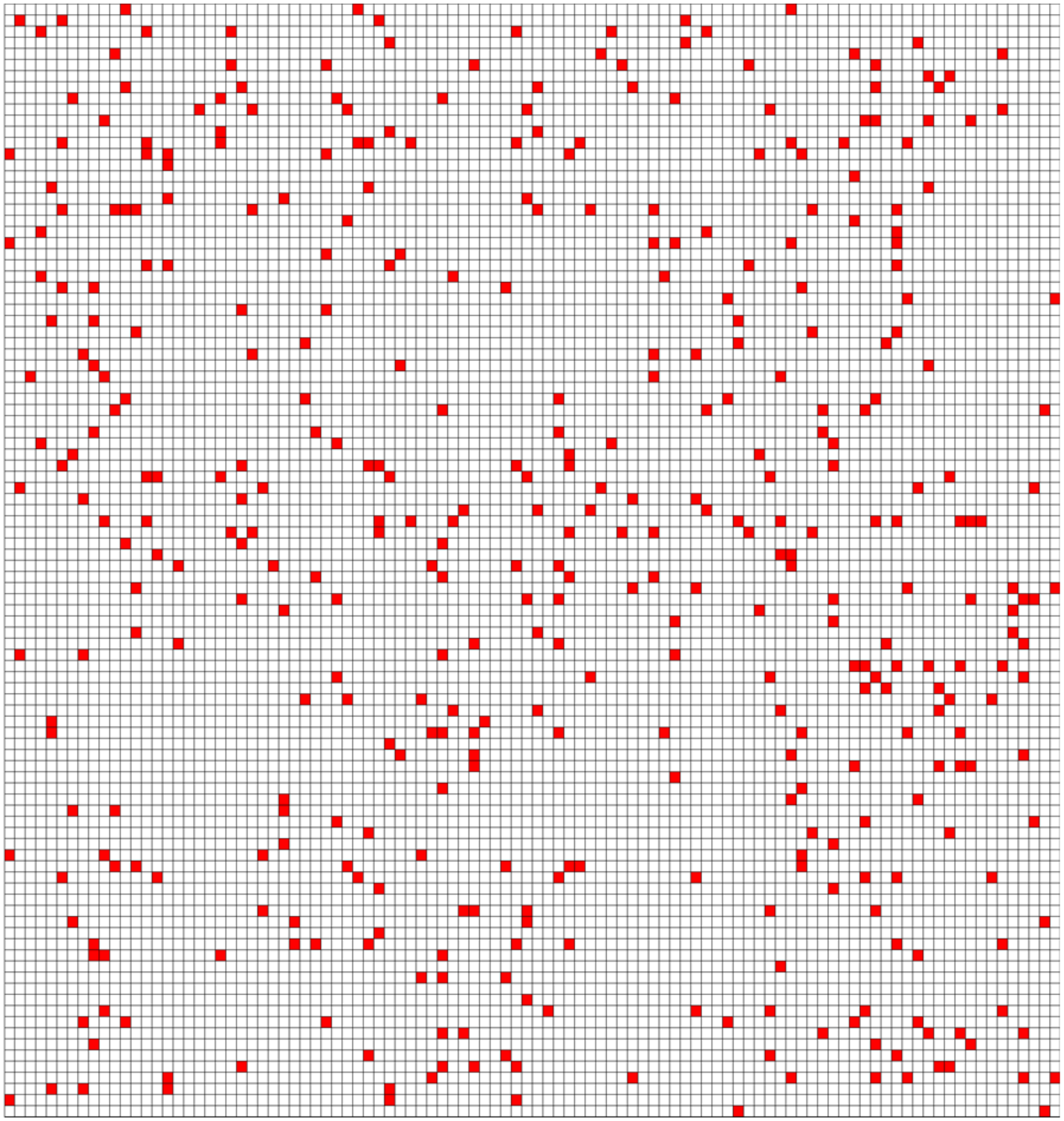

SURVEY 2

Age **50-59**: Chance of Dying During the Next 10 Years = **870** out of 10,000 (or 8.70%).

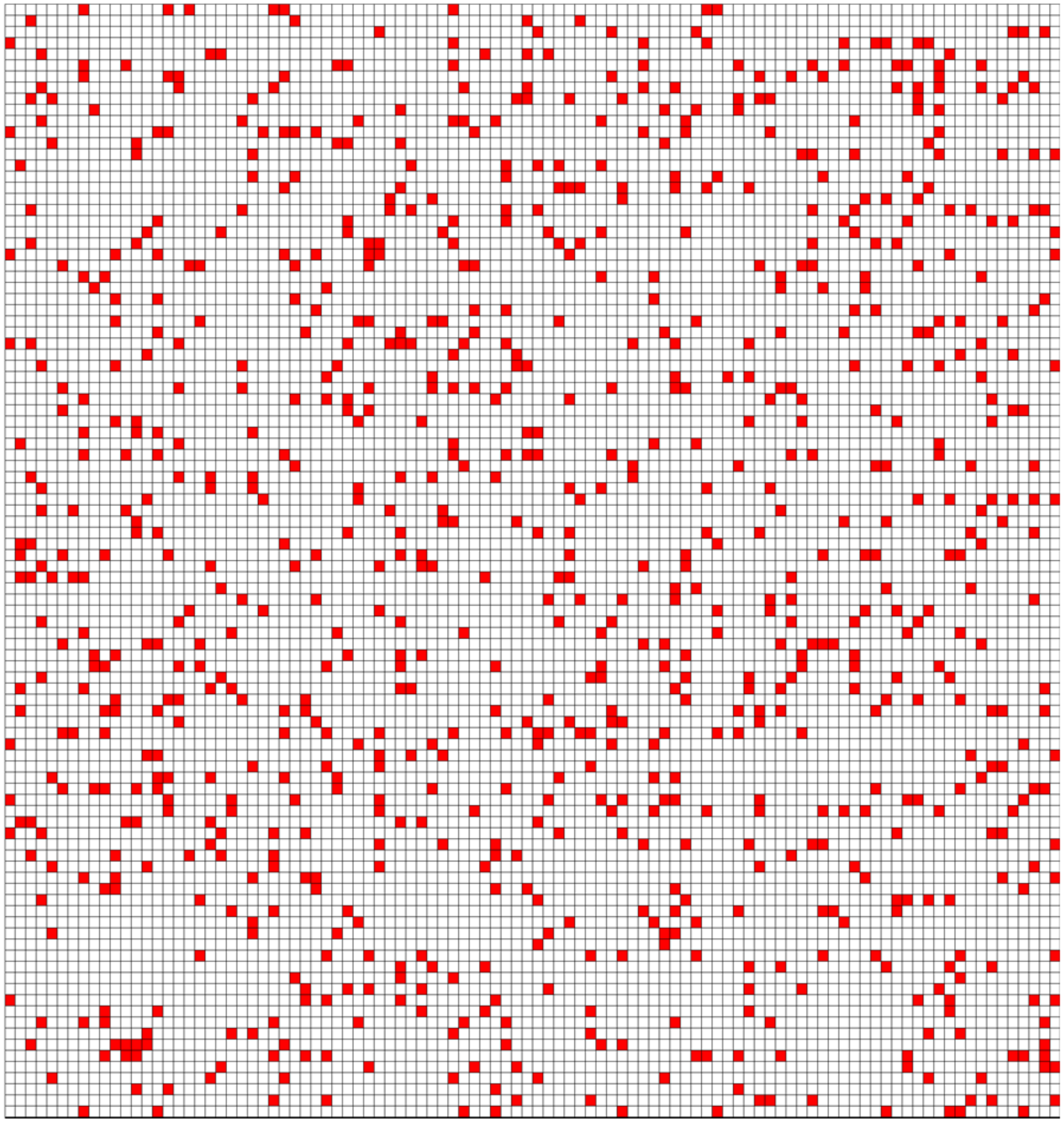

SURVEY 2

Age **60-69**: Chance of Dying During the Next 10 Years = **1,883** out of 10,000 (or 18.83%).

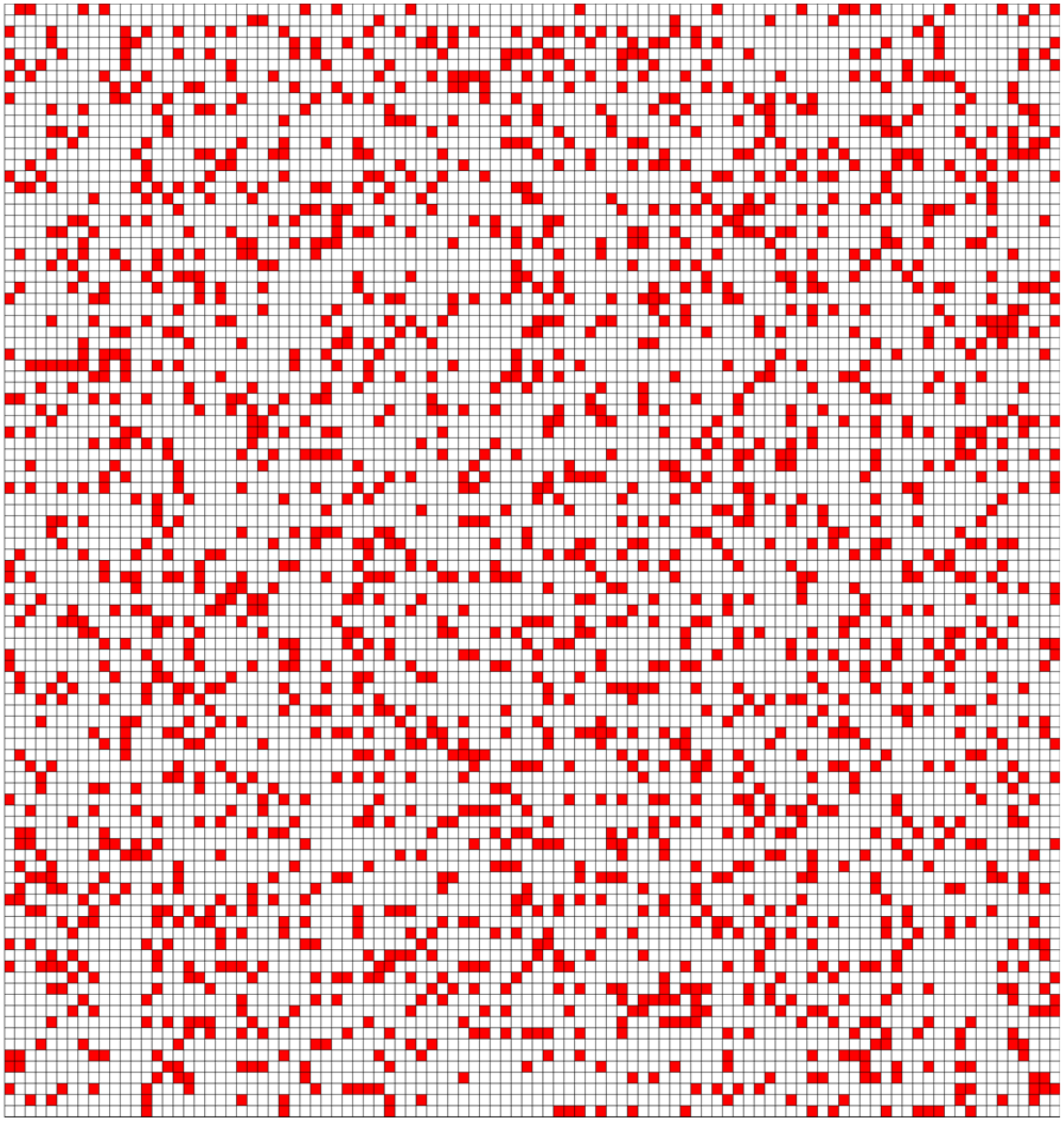

SURVEY 2

Age **70-79**: Chance of Dying During the Next 10 Years = **4,193** out of 10,000 (or 41.93%).

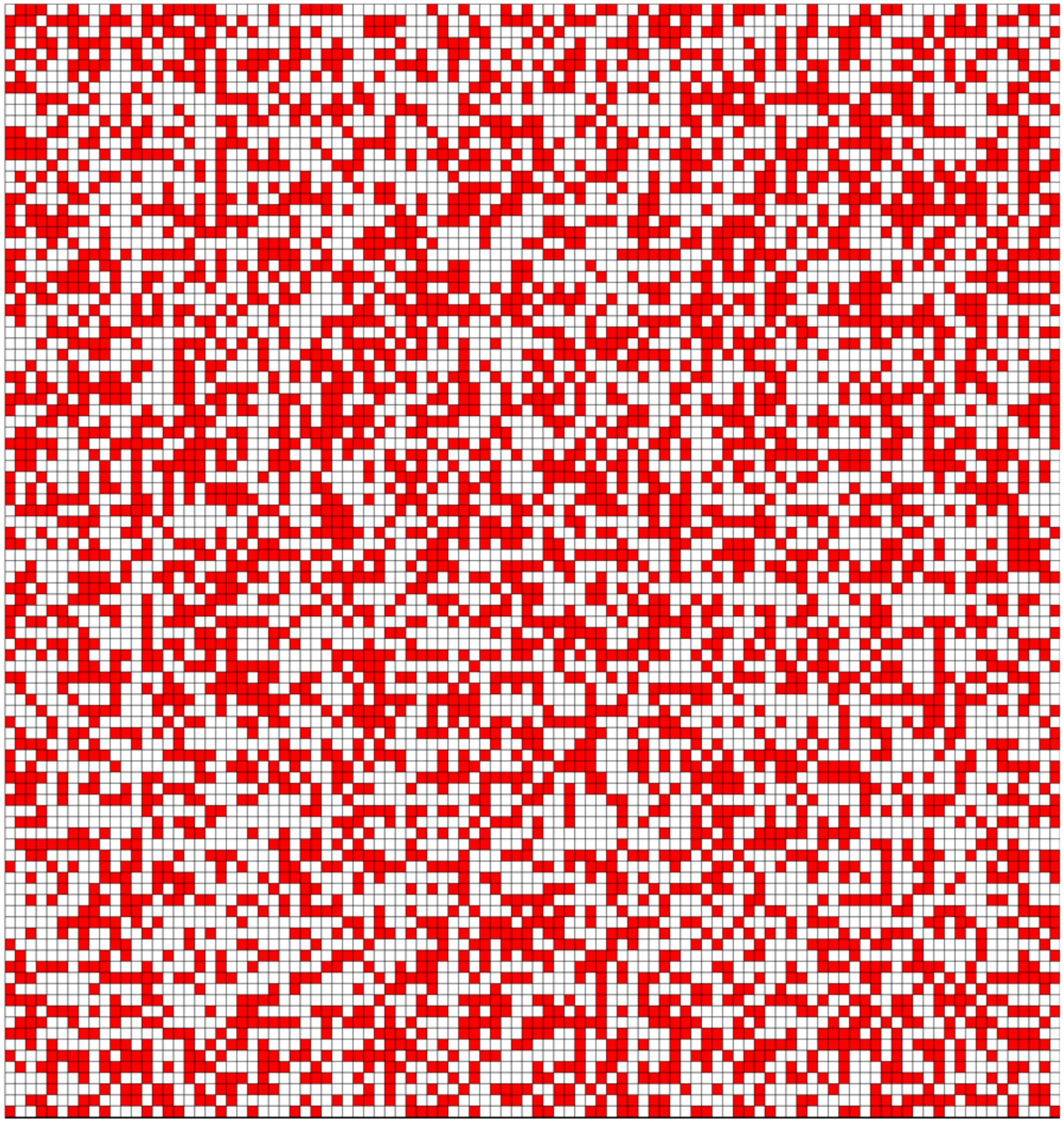

SURVEY 2

Age **80-89**: Chance of Dying During the Next 10 Years = **7,432** out of 10,000 (or 74.32%).

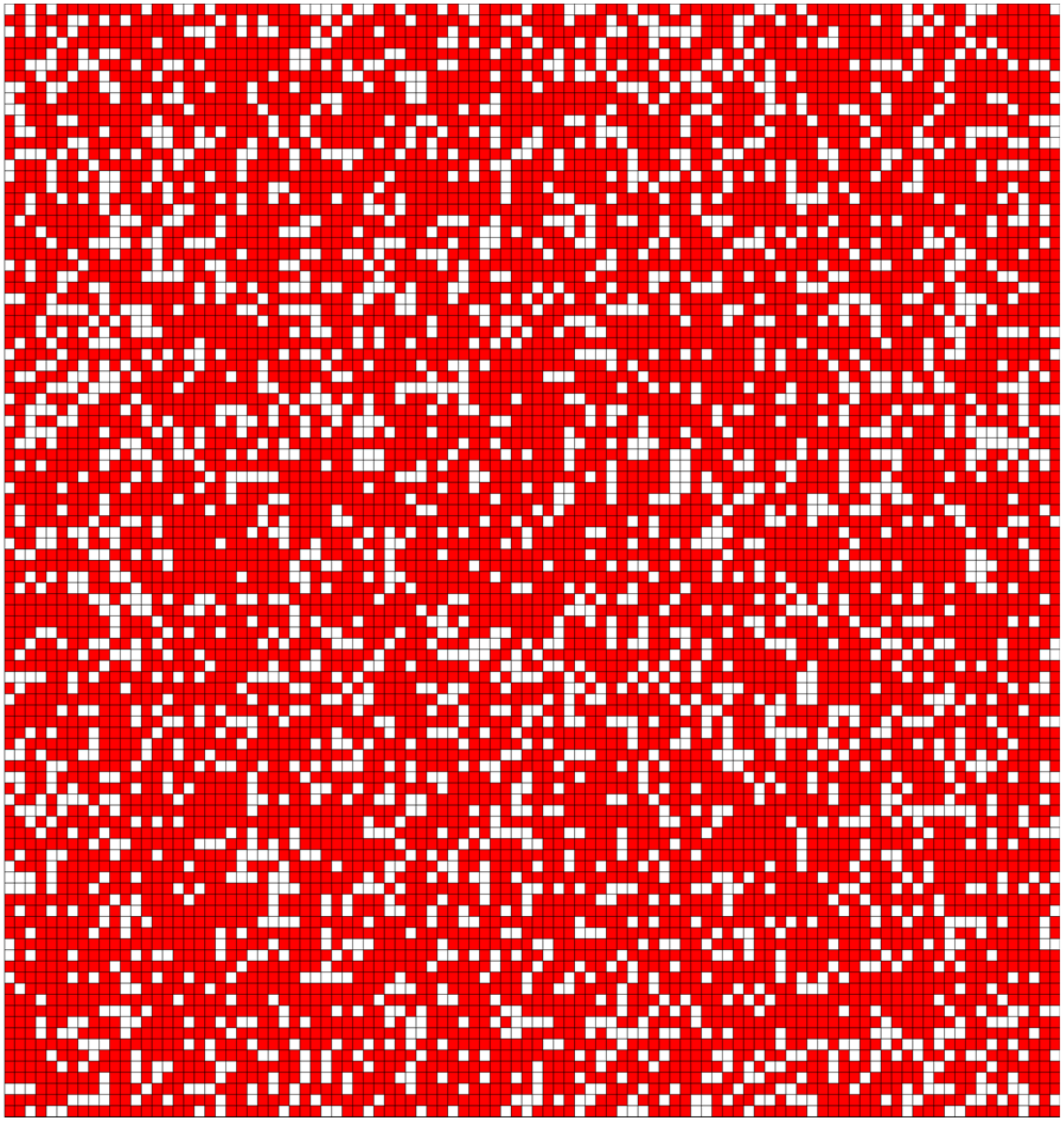

Preview to Question 7 (continued)

Take the case of someone age 30-39 years old.

Out of 10,000 people age 30-39 years old, 186 die within 10 years.

Imagine a roulette ball randomly landing in one of the squares on the grid.

If the ball lands in a white square, the person lives at least 10 more years.

Age **30-39**: Chance of Dying During the Next 10 Years = **186** out of 10,000.

(Result: Lives at least 10 more years)

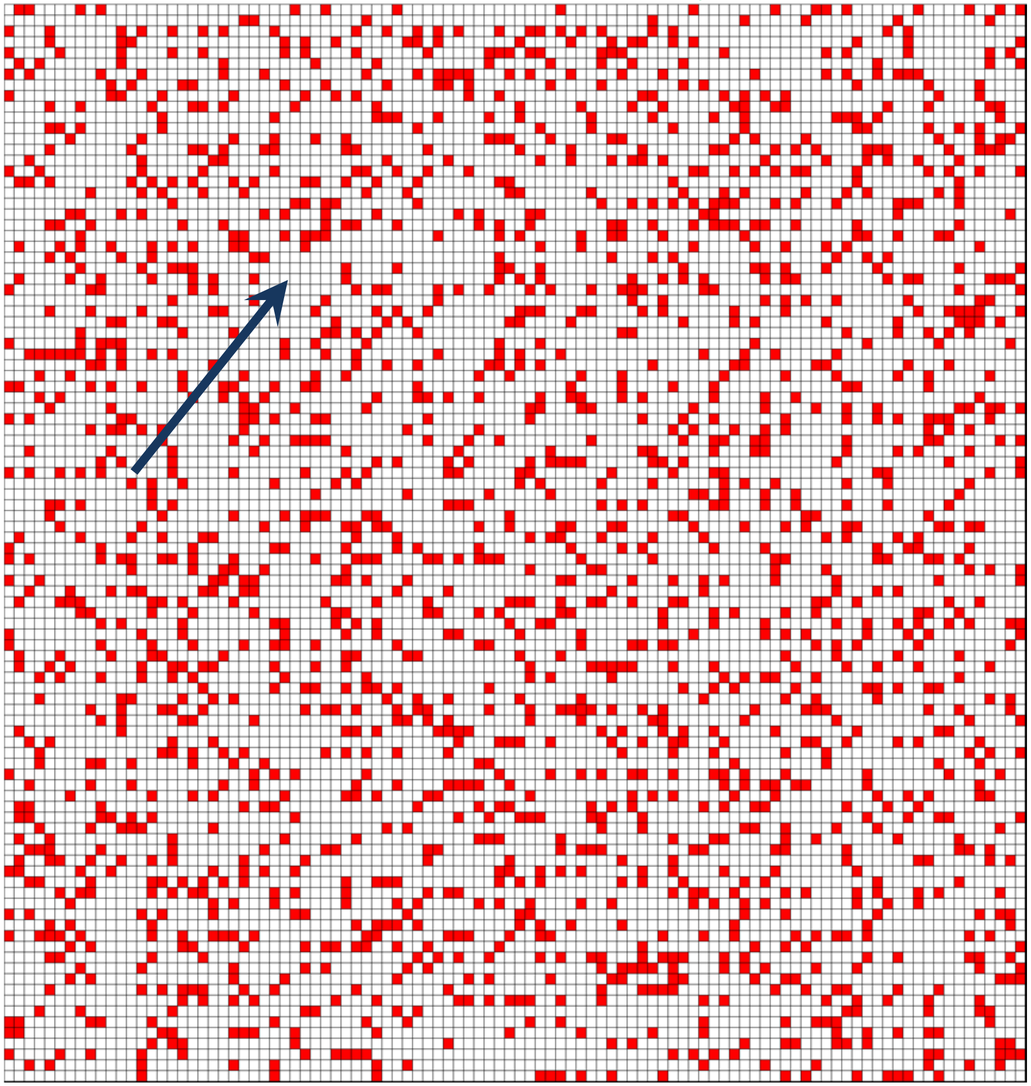

Preview to Question 7 (continued)

If the ball lands in a red square the person dies within the next 10 years.

Out of 10,000 spins of the roulette wheel, we would expect the ball to land in a red square 186 times.

Age **30-39**: Chance of Dying During the Next 10 Years = **186** out of 10,000.

(Result: Dies within the next 10 years)

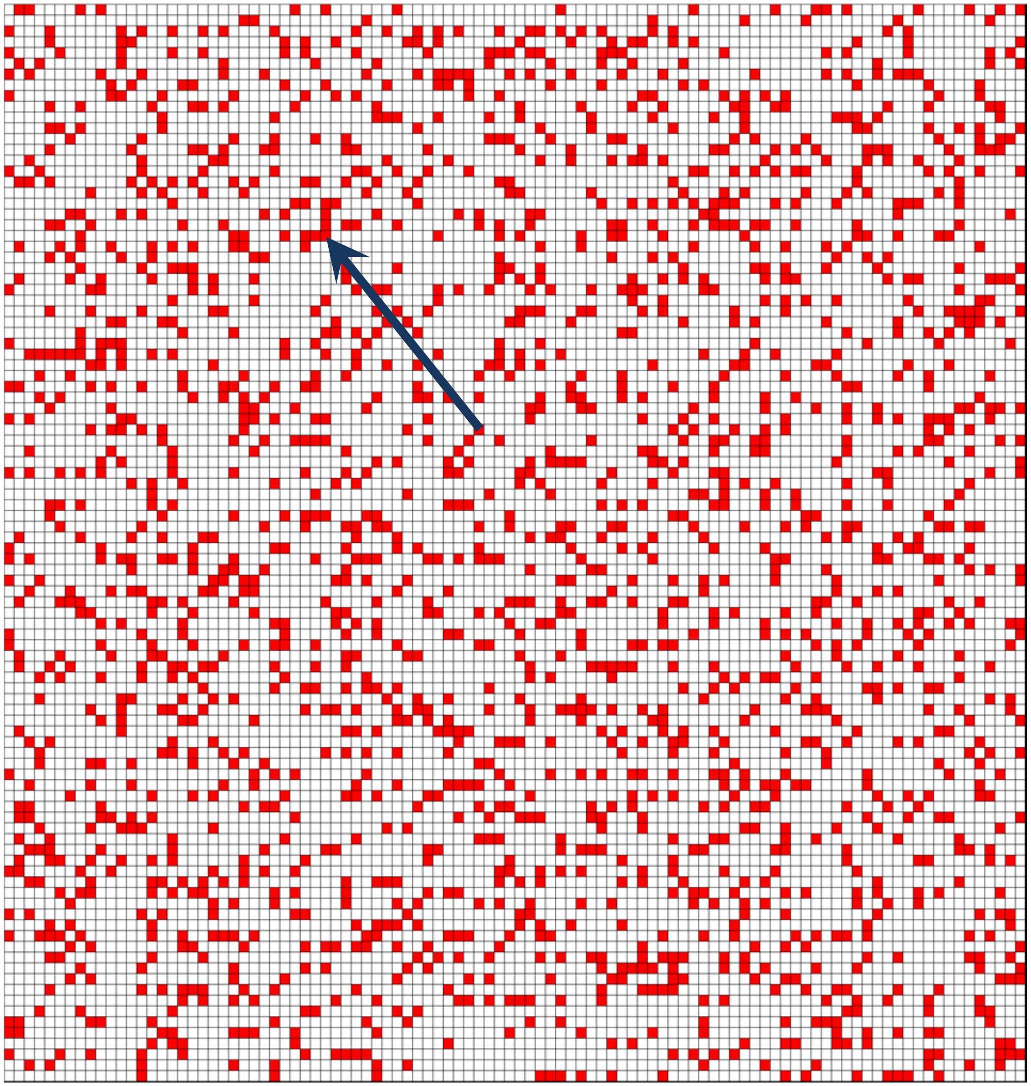

Preview to Question 7 (continued): Medical Products & Safety Inventions

Now, imagine that a company decided to give out special medical products and/or safety inventions that could lower a person's chance of dying during the next 10 years. A medical "product" could include things like drugs, immunizations, and new medical screening technologies that can catch diseases and cancers while they are still treatable. Life-saving inventions include things like car safety devices that lower the risk of serious accidents or technologies that help prevent accidental drowning, poisoning, falling, electrocution, etc. Assume that these products and inventions have no side effects and that these products / inventions are not available on the market and can be obtained only by the company's donation to a person.

Assume that the company has decided to give out some of these medical products and/or safety inventions for free and they will be given to either you or another person. Once they are given out, assume that they cannot be given or sold to someone else. The company will not reveal the name of the recipient to the public.

Each product or invention given will lower the recipient's chance of dying during the next 10 years by one chance in 10,000. Thus, if the company gives 5 medical products or inventions to a person, that person's chance of dying during the next 10 years would decline by 5 chances in 10,000.

Preview to Question 7 (continued)

Imagine 10 medical products or safety inventions are given to a 30-39 year old.

The 10 products or inventions would lower the person's chance of dying during the next 10 years from: 186 out of 10,000
to: 176 out of 10,000.

The change in the person's chance of death is shown in the following figure. The 10 blue squares were previously red squares, and reflect the reduction in the chance of death that is caused by the receipt of the 10 medical products or safety inventions.

Age **30-39**: Chance of Dying During Next 10 Years = ***176*** out of 10,000 after the donation by the company.

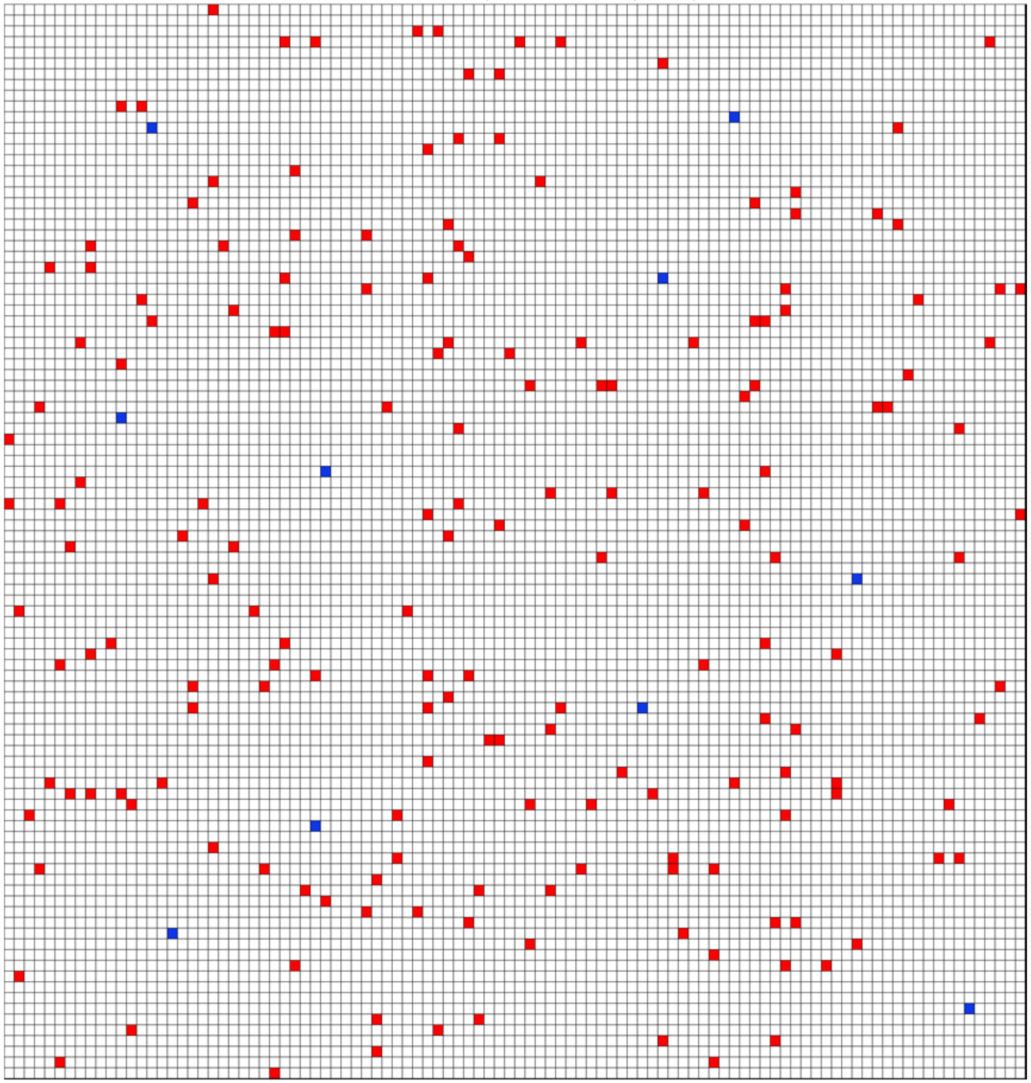

Preview to Question 7 (continued)

If the roulette ball happens to land in one of these 10 blue squares, then the person's life is extended by the person having the 10 new products/inventions.

Age **30-39**: Chance of Dying During Next 10 Years = ***176*** out of 10,000 after the donation by the company. *(Result: Saved by Product)*

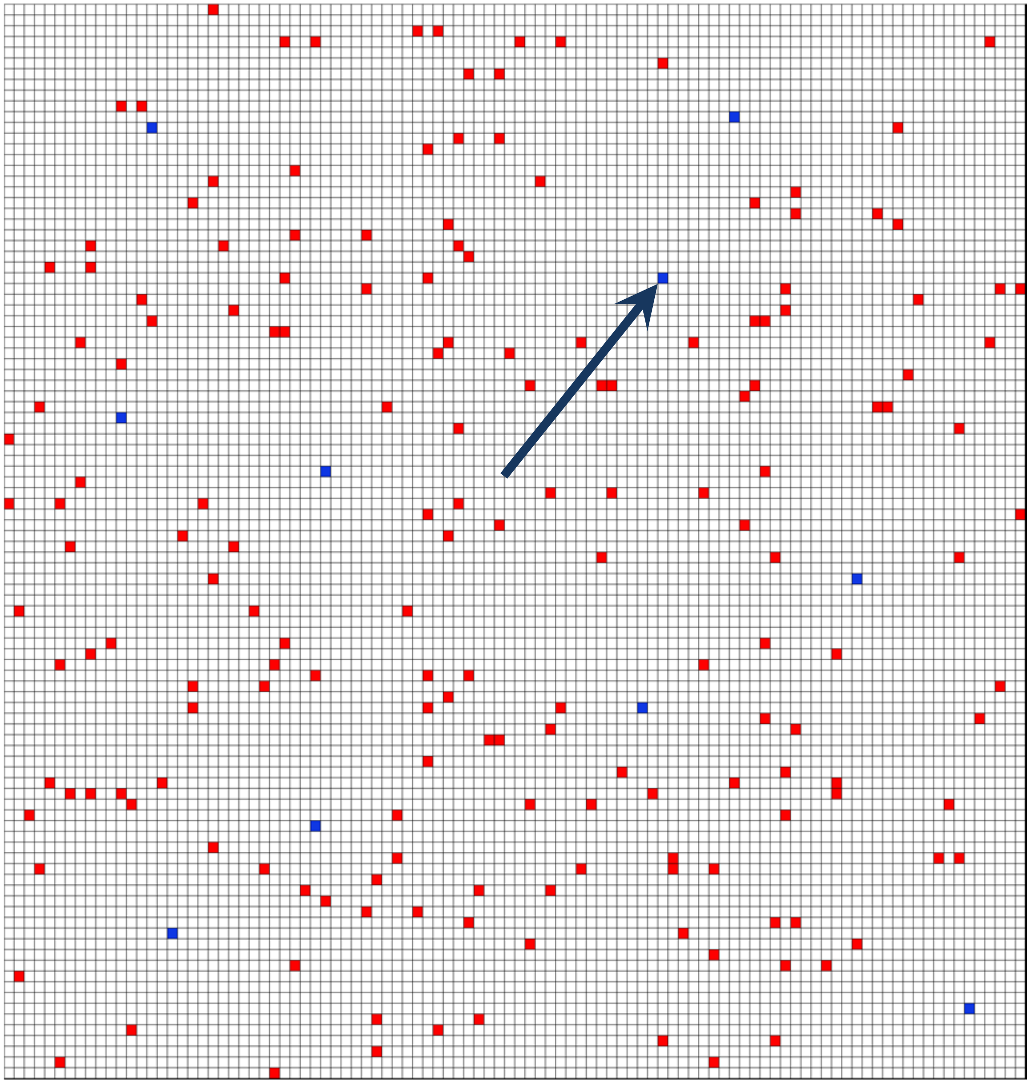

SURVEY 2

Preview to Question 7 (continued)

If the ball lands in a red square, the person dies during the next 10 years, and this reflects a death that the 10 new products / inventions could not prevent.

Age **30-39**: Chance of Dying During Next 10 Years = ***176*** out of 10,000 after the donation by the company. *(Result: Dies Despite Having 10 Products)*

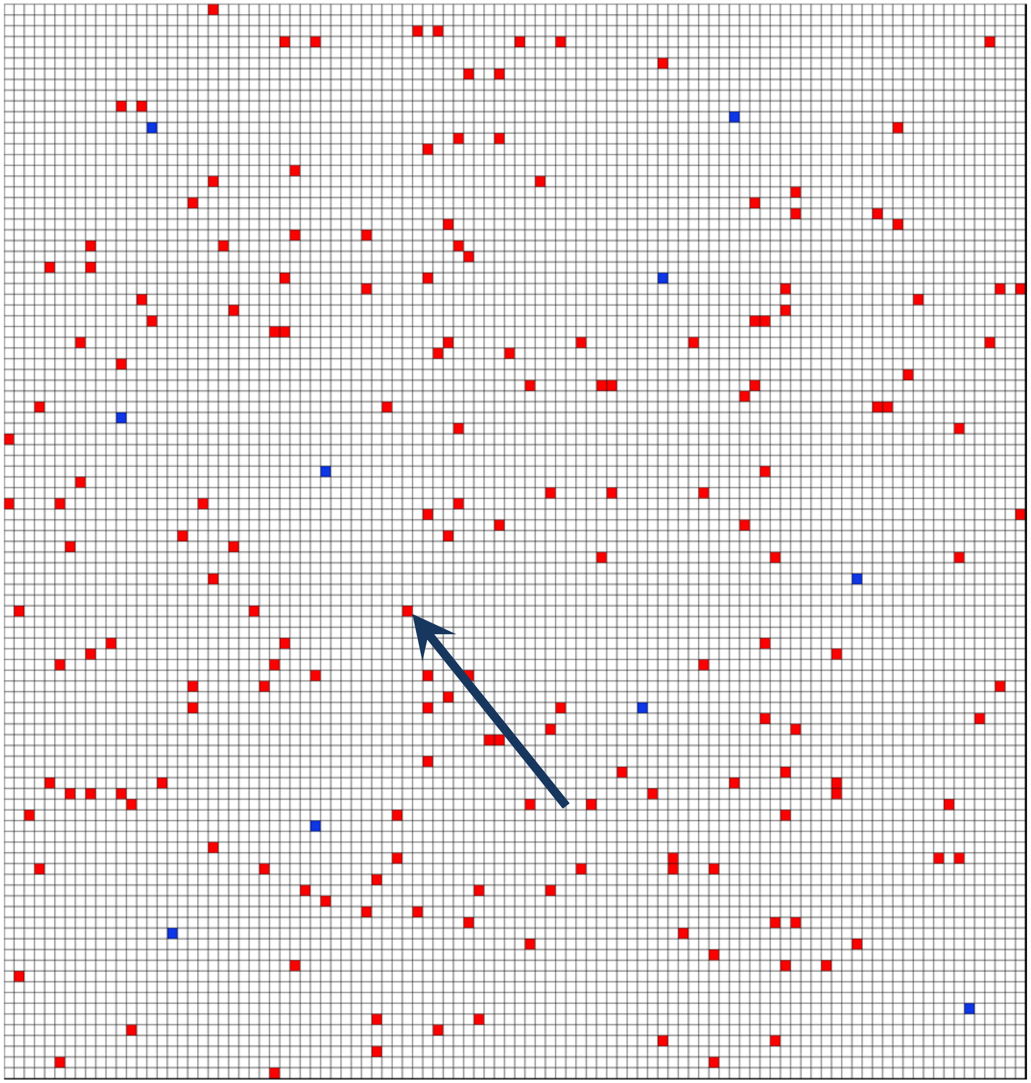

Preview to Question 7 (continued)

Finally, if the ball lands in a white square, the person does not die during the next 10 years for reasons unrelated to having the 10 new products / inventions.

Age **30-39**: Chance of Dying During Next 10 Years = ***176*** out of 10,000 after the donation by the company. *(Result: Lives 10 Years Not Due to Products)*

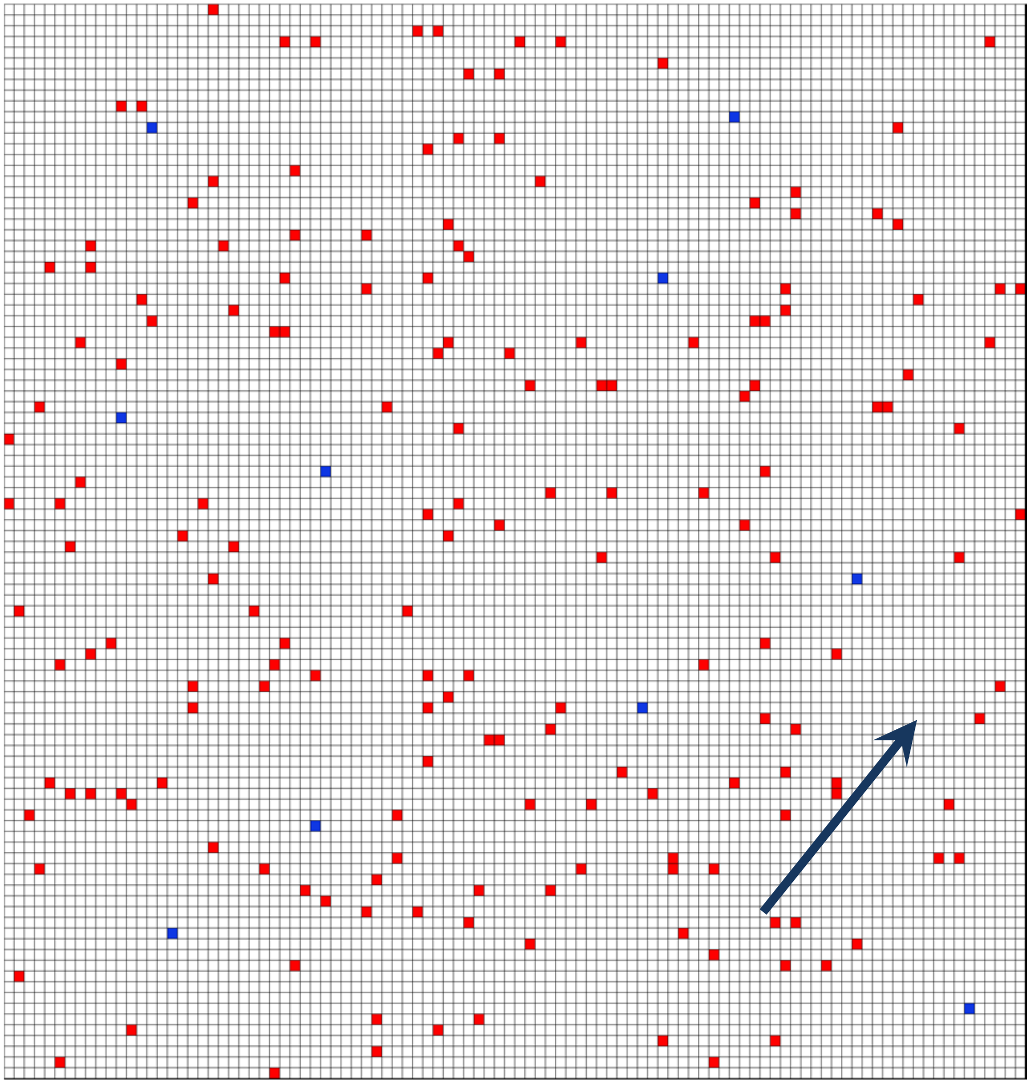

Preview to Question 7 (continued)

In Question 7, you will be asked to decide if the company should either give **you** a given number of medical products or safety inventions or give some **other person** a given number of medical products or safety inventions. The company will not give these medical products or safety inventions to anyone else. And, remember that these products/inventions are not available on the market and can be obtained only by the company's donation to a person.

You will be told the age range of the other person (for example, age 60-69), and their relationship to you (for example, "an acquaintance"). Assume that the other person will not know that you have been asked to make this choice of who will receive the products/inventions. That is, your choice is unknown to the other person.

You will be asked several versions of this question, with each version varying the number of inventions/products offered to you, the number of inventions/products offered to the other person, the age of the other person, or the relationship of the other person to you.

In each question, you will be asked to select whether you would like the products/inventions to go to you or to the other person.

Before we get to these questions, the next few questions are included to make sure you understand the concepts related to the chance of death within the next 10 years.

SURVEY 2

Preview to Question 7. Warm-Up Question A: Which person has the *lower* chance of dying in the next 10 years?

Person 1:

☐ Chance of dying within the next 10 years is *755* out of 10,000.

Person 2:

☐ Chance of dying within the next 10 years is *382* out of 10,000.

If the respondent answers Person 2, show the following text: "Correct! Person 2 has a lower chance of dying in the next 10 years than person 1."
If the respondent answers Person 1, show the following text: "Not Correct. Person 1 has a higher chance of dying in the next 10 years than person 2."

SURVEY 2

Preview to Question 7. Warm-Up Question A (Try Again): Which person has the *lower* chance of dying in the next 10 years?

Person 1:
☐ Chance of dying within the next 10 years is *127* out of 10,000.

Person 2:
☐ Chance of dying within the next 10 years is *214* out of 10,000.

If the respondent answers Person 1, show the following text: "Correct! Person 1 has a lower chance of dying in the next 10 years than person 1."
If the respondent answers Person 2, show the following text: "Not Correct. Person 2 has a higher chance of dying in the next 10 years than person 2."

SURVEY 2

Preview to Question 7. Warm-Up Question B: Which person would you rather be (assuming that you want to live for 10 years)?

Person 1:

☐ Chance of dying within the next 10 years is *159* out of 10,000.

Person 2:

☐ Chance of dying within the next 10 years is *537* out of 10,000.

If the respondent answers Person 1, show the following text: "Correct! Person 1 has a lower chance of dying in the next 10 years than person 1."
If the respondent answers Person 2, show the following text: "Not Correct. Person 2 has a higher chance of dying in the next 10 years than person 2."

Preview to Question 7. Warm-Up Question B (Try Again): Which person would you rather be (assuming that you want to live for 10 years)?

Person 1:

☐ Chance of dying within the next 10 years is *1,534* out of 10,000.

Person 2:

☐ Chance of dying within the next 10 years is *9,347* out of 10,000.

If the respondent answers Person 1, show the following text: "Correct! Person 1 has a lower chance of dying in the next 10 years than person 1."
If the respondent answers Person 2, show the following text: "Not Correct. Person 2 has a higher chance of dying in the next 10 years than person 2."

SURVEY 2

Preview to Question 7. Warm-Up Question C: Which person has the biggest reduction in his/her chance of death during the next 10 years as a result of the company's donation of medical products or safety inventions?

Person 1:

☐ Company gives this person *10* medical products / safety inventions.

Person 2:

☐ Company gives this person *6* medical products / safety inventions.

If the respondent answers Person 1, show the following text: "Correct! The reduction in the number of chances of dying during the next 10 years is greater for person 1 than person 2."

If the respondent answers Person 2, show the following text: "Not Correct. The reduction in the number of chances of dying during the next 10 years is smaller for person 2 than person 1."

SURVEY 2

Preview to Question 7. Warm-Up Question C (Try Again): Which person has the biggest reduction in his/her chance of death during the next 10 years as a result of the company's donation of medical products or safety inventions?

☐ **Person 1:**
Company gives this person *12* medical products / safety inventions.

☐ **Person 2:**
Company gives this person *4* medical products / safety inventions.

If the respondent answers Person 1, show the following text: "Correct! The reduction in the number of chances of dying during the next 10 years is greater for person 1 than person 2."
If the respondent answers Person 2, show the following text: "Not Correct. The reduction in the number of chances of dying during the next 10 years is smaller for person 2 than person 1."

SURVEY 2

Question 7
Select the option that you most prefer:

Remember: Each product/invention increases the recipient's chance of surviving 10 years by 1 chance in 10,000.

☐ **Option 1:**
The company gives 8 medical products or safety inventions to you (and gives nothing to the other person).

☐ **Option 2:**
The company gives 8 medical products or safety inventions to a 40-49 year old acquaintance (and gives nothing to you).

SURVEY 2

Preview to Question 8: Chance of Winning $25,000

Question 8 is about the chance of you or others winning money.

Imagine that a company decided to give out scratch-off tickets and that each ticket has one chance in 10,000 of winning $25,000 from the company. Only one prize will be given. Thus, if the company gives 5 scratch-off tickets to a person, that person's chance of winning $25,000 would be 5 chances in 10,000.

Assume that the company has decided to give out these scratch-off tickets for free and they will be given to either you or another person. Once they are given out, assume that they cannot be given or sold to someone else. The company will not reveal the name of the recipient to the public. No one else is eligible to participate and the tickets can only be obtained only by the company's donation to a person.

Preview to Question 8 (continued)

Chance of having winning $25,000 = **10** out of 10,000.

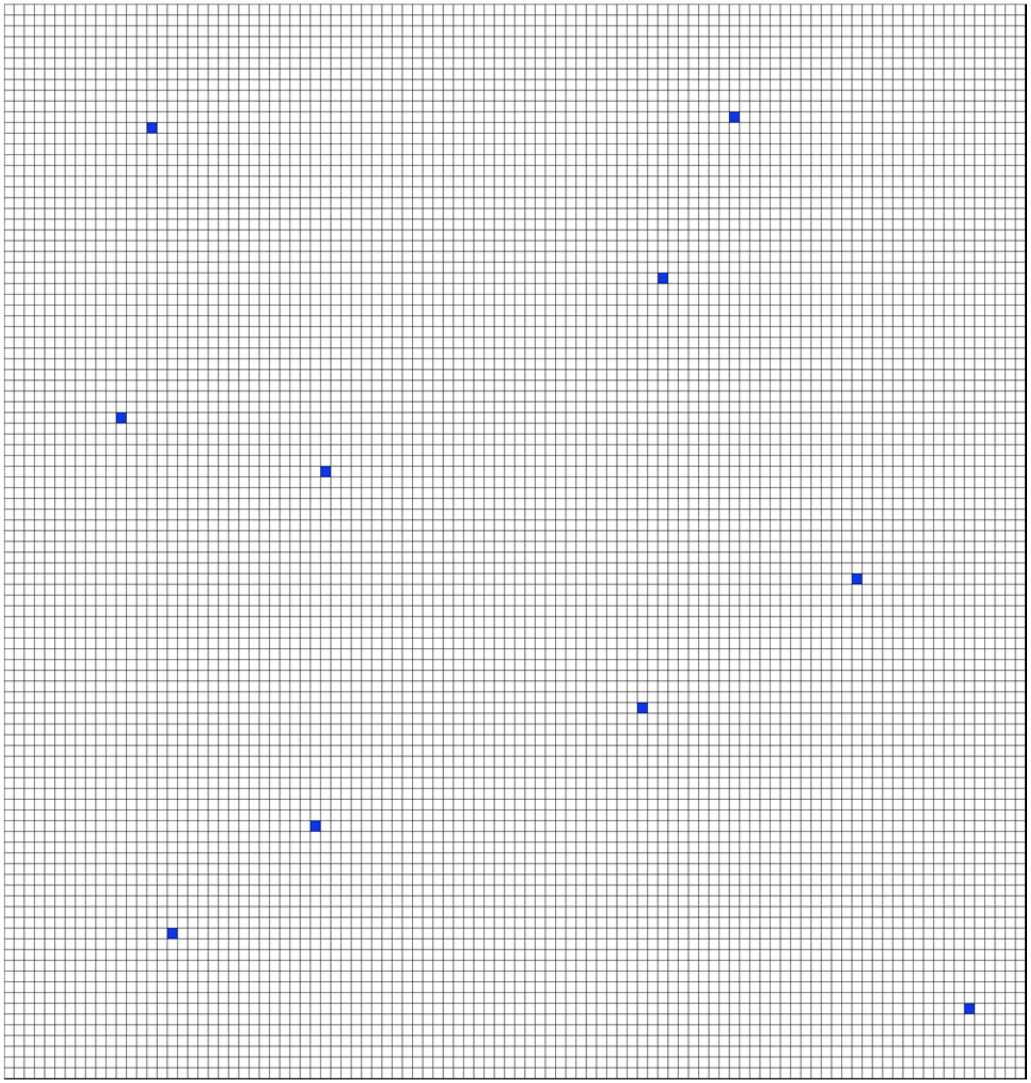

There are 10,000 squares shown. You can think of each square like the slot on a roulette wheel, where if by chance the roulette ball landed in a blue square, the person will win $25,000.

A person with 10 tickets has 10 chances in 10,000 to win $25,000.

Preview to Question 8 (continued)

If the ball lands in a blue square the person will receive $25,000.

Out of 10,000 spins of the roulette wheel, we would expect it to land on a blue square 10 times.

Chance of having winning $25,000 = **10** out of 10,000.

(Result: Wins $25,000)

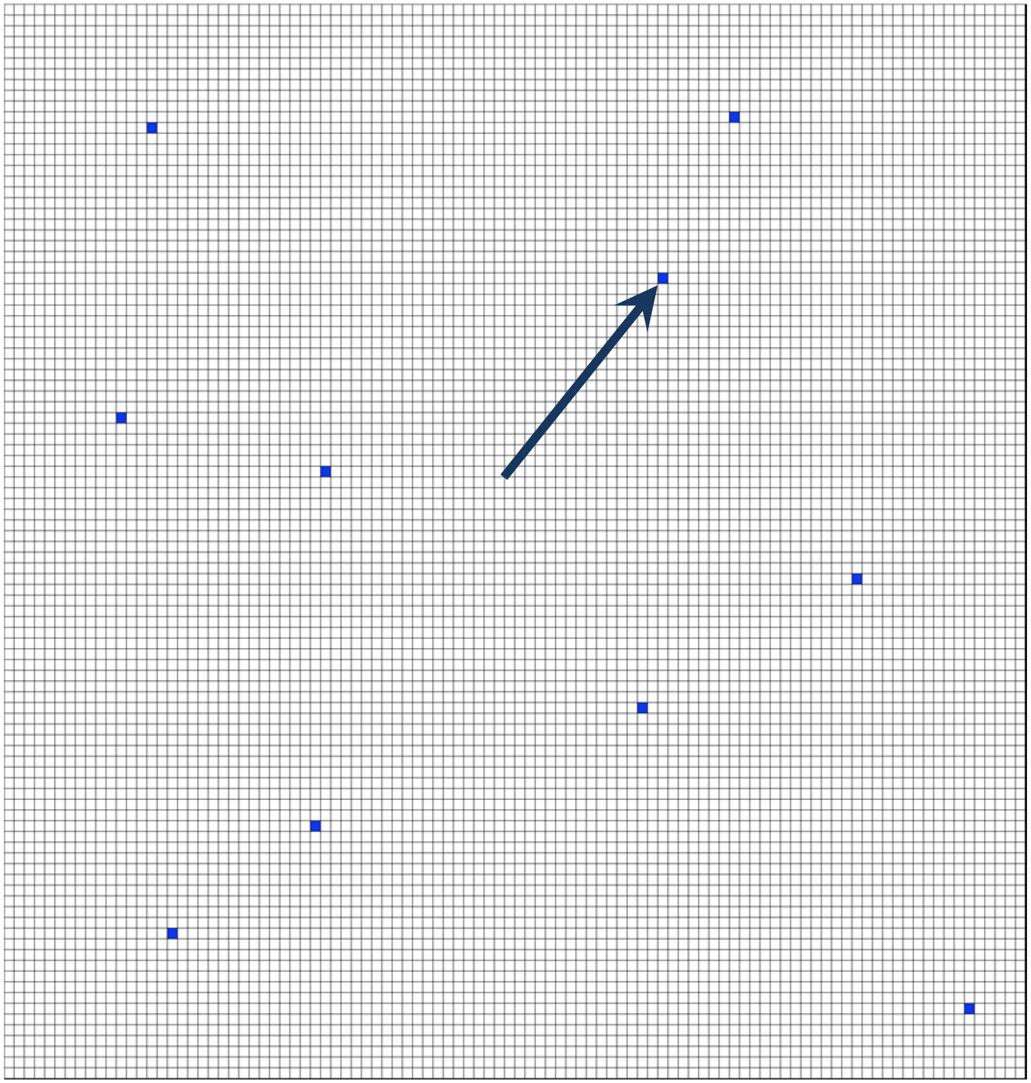

Preview to Question 8 (continued)

If the ball lands in a white square, the person will receive nothing.

Chance of Winning $25,000 = **10** out of 10,000.

(Result: Does Not Win $25,000)

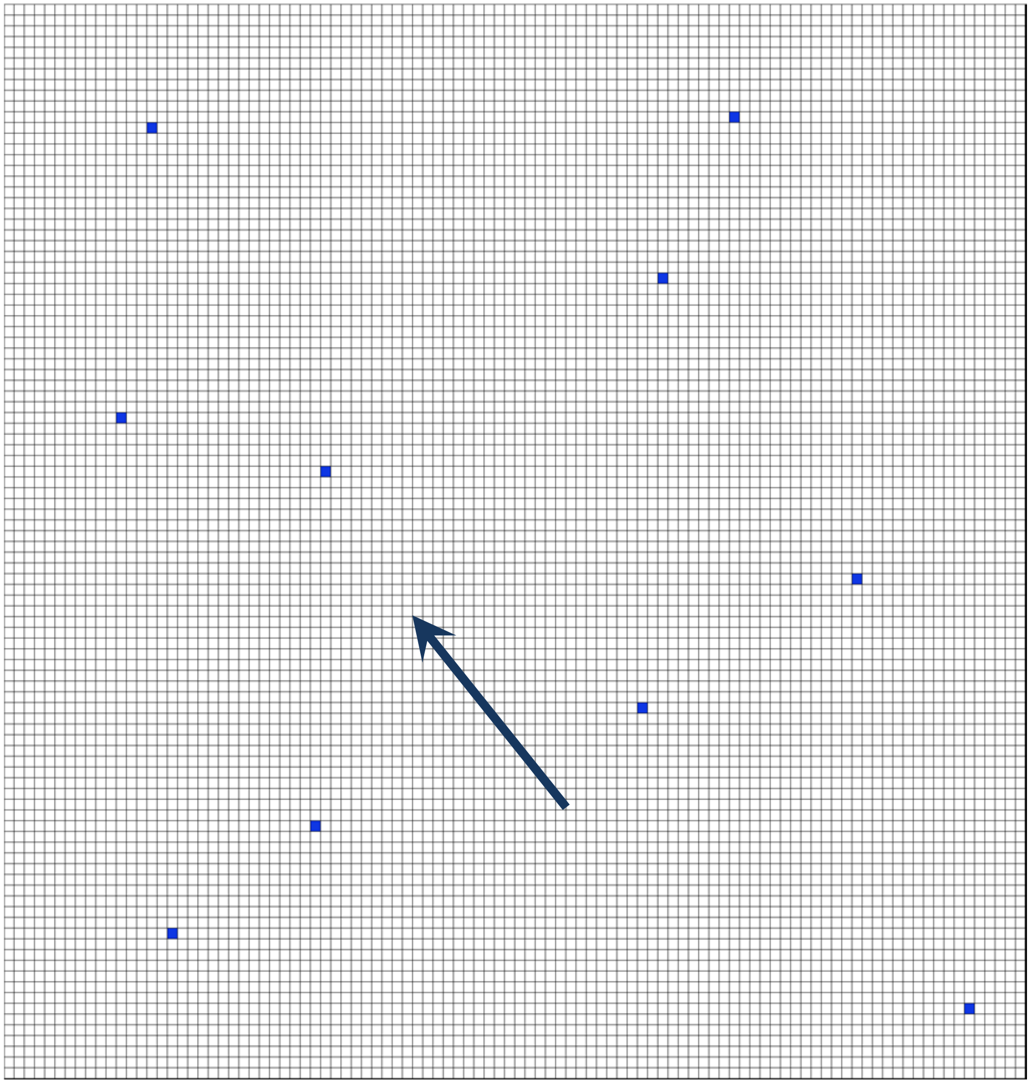

Preview to Question 8 (continued)

In Question 8, you will be asked to decide if the company should either give **you** a given number of scratch-off tickets or give some **other person** a given number of scratch-off tickets. The company will not give scratch-off tickets to anyone else. And, remember that these scratch-off tickets can be obtained only by the company's donation to a person.

You will be told the age range of the other person (for example, age 60-69), and their relationship to you (for example, "an acquaintance"). Assume that the other person will not know that you have been asked to make this choice to deal out the tickets. That is, your choice is unknown to the other person.

You will be asked several versions of this question, with each version varying the number of scratch-off tickets offered to you, the number of scratch-off tickets offered to the other person, the age of the other person, or the relationship of the other person to you.

In each question, you will be asked to select whether you would like the scratch-off tickets to go to you or to the other person.

Before we get to these questions, the next few questions are included to make sure you understand the concepts related to the chance of winning money from the company.

SURVEY 2

Preview to Question 8. Warm-Up Question A: Which person has the higher chance of having a winning ticket?

Person 1:

☐ Company gives this person *13* scratch-off tickets.

Person 2:

☐ Company gives this person *3* scratch-off tickets.

If the respondent answers Person 1, show the following text: "Correct! Person 1 has a higher chance of having a winning ticket than person 2."
If the respondent answers Person 2, show the following text: "Not Correct. Person 2 has a lower chance of having a winning ticket than person 1."

SURVEY 2

Preview to Question 8. Warm-Up Question A (Try Again): Which person has the higher chance of having a winning ticket?

Person 1:
☐ Company gives this person *2* scratch-off tickets.

Person 2:
☐ Company gives this person *14* scratch-off tickets.

If the respondent answers Person 2, show the following text: "Correct! Person 2 has a higher chance of having a winning ticket than person 1."
If the respondent answers Person 1 show the following text: "Not Correct. Person 1 has a lower chance of having a winning ticket than person 2."

SURVEY 2

Preview to Question 8. Warm-Up Question B: Which person would you rather be (assuming that you want to win $25,000 from the company)?

Person 1:

☐ Company gives this person *7* scratch-off tickets.

Person 2:

☐ Company gives this person *9* scratch-off tickets.

If the respondent answers Person 2, show the following text: "Correct! Person 2 has a higher chance of having a winning ticket than person 1."
If the respondent answers Person 1 show the following text: "Not Correct. Person 1 has a lower chance of having a winning ticket than person 2."

SURVEY 2

Preview to Question 8. Warm-Up Question B (Try Again): Which person would you rather be (assuming that you want to win $25,000 from the company)?

Person 1:

☐ Company gives this person *5* scratch-off tickets.

Person 2:

☐ Company gives this person *11* scratch-off tickets.

If the respondent answers Person 2, show the following text: "Correct! Person 2 has a higher chance of having a winning ticket than person 1."

If the respondent answers Person 1 show the following text: "Not Correct. Person 1 has a lower chance of having a winning ticket than person 2."

SURVEY 2

Question 8
Select the option that you most prefer:

Remember: Each scratch-off ticket increases the recipient’s chance of winning $25,000 by 1 chance in 10,000.

☐ **Option 1:**
The company gives 8 scratch-off tickets to you (and gives nothing to the other person).

☐ **Option 2:**
The company gives 8 scratch-off tickets to a 40-49 year old acquaintance (and gives nothing to you).

SURVEY 2

Question ф: How would you rate your undertanding of probability?
(Click one box)

(A) I do not understand probability at all ☐
(B) I have a poor understanding of probability ☐
(C) I have a fair understanding of probability ☐
(D) I have a good understanding of probability ☐
(E) I have an excellent understanding of probability ☐

SURVEY 2

Question 1л: What country or region werŜ you born in? (Click one box)

North America

- United States of America
- Canada
- Mexico
- El Salvador
- Guatemala
- Cuba
- Dominican Republic
- Other Country in North America (including Central America)

Asia

- China
- India
- Phillipines
- Vietnam
- Other Country in Asia

Europe
South America
Africa
Australia or New Zealand
Other Pacific Island
Other Region

SURVEY 2

Question 11: What state were you born in? (Click one box)

Alaska	☐	Maryland	☐	South Carolina	☐
Alabama	☐	Maine	☐	South Dakota	☐
Arkansas	☐	Michigan	☐	Tennessee	☐
Arizona	☐	Minnesota	☐	Texas	☐
California	☐	Missouri	☐	Utah	☐
Colorado	☐	Mississippi	☐	Virginia	☐
Connecticut	☐	Montana	☐	Vermont	☐
District of Columbia	☐	North Carolina	☐	Washington	☐
Delaware	☐	North Dakota	☐	Wisconsin	☐
Florida	☐	Nebraska	☐	West Virginia	☐
Georgia	☐	New Hampshire	☐	Wyoming	☐
Hawaii	☐	New Jersey	☐		
Iowa	☐	New Mexico	☐		
Idaho	☐	Nevada	☐	American Samoa, Guam, Puerto Rico, Virgin Islands, or Other	☐
Illinois	☐	New York	☐		
Indiana	☐	Ohio	☐		
Kansas	☐	Oklahoma	☐		
Kentucky	☐	Oregon	☐		
Louisiana	☐	Pennsylvania	☐		
Massachusetts	☐	Rhode Island	☐		
