# Supplementary material for: Altruism by age and social proximity
Source: PLoS One. 2017 Aug 24;12(8):e0180411. doi: 10.1371/journal.pone.0180411 (PMC5570493; doi:10.1371/journal.pone.0180411)

Question Progression for Survey 2

Panel A: One-third of the question sets started with an even allocation

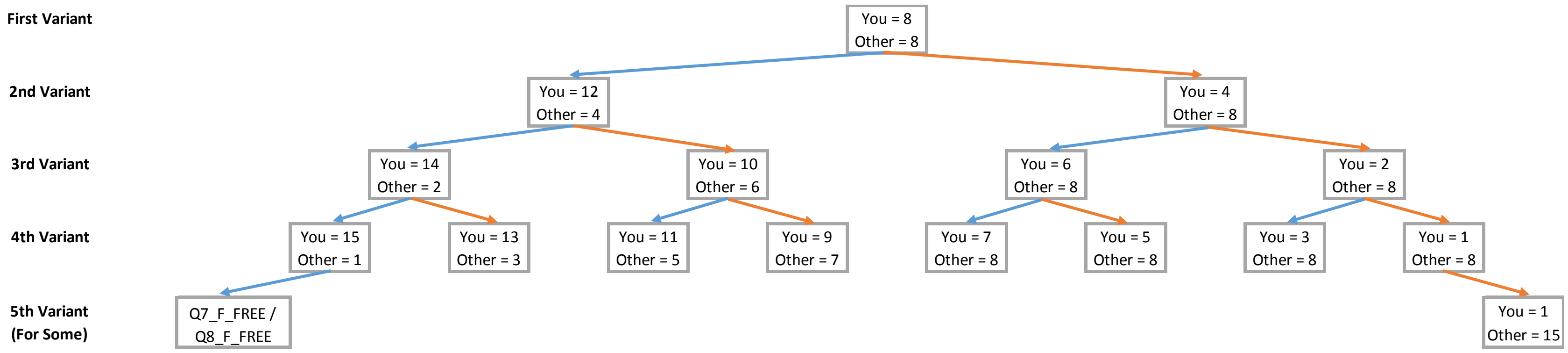

Orange Arrows Denote Respondent Chose "You"
Blue Arrows Denote Respondent Chose "Other"

Panel B: One-third of the question sets started with a less generous initial allocation

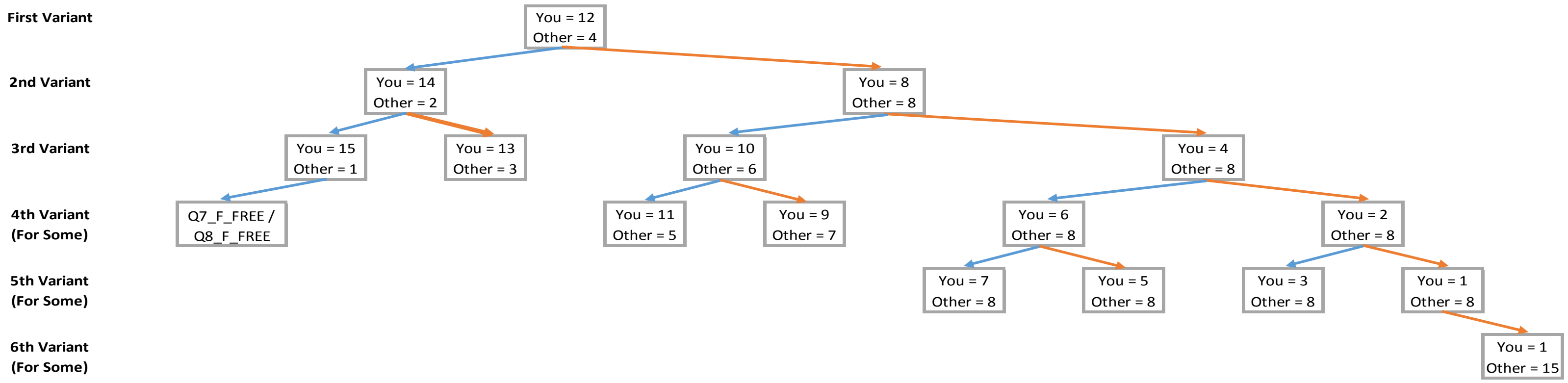

Orange Arrows Denote Respondent Chose "You"
Blue Arrows Denote Respondent Chose "Other"

Question Progression for Survey 2 (Continued)

Panel C: One-third of the question sets started with a more generous initial allocation

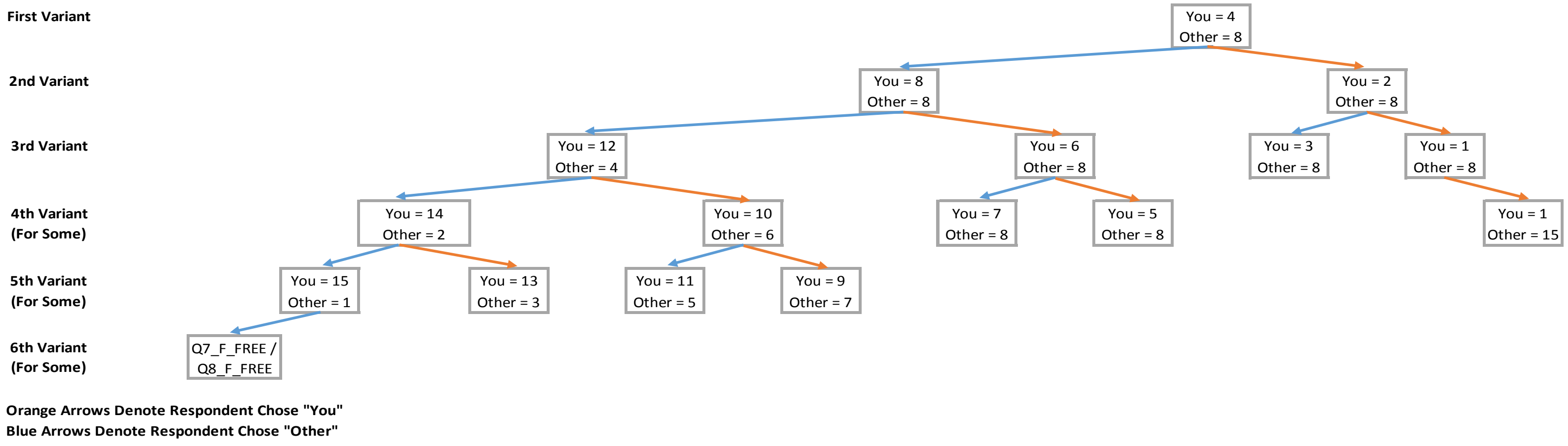

Supplement: S1 Fig — (PDF) [file pone.0180411.s003.pdf]
