# Supplementary material for: Altruism by age and social proximity
Source: PLoS One. 2017 Aug 24;12(8):e0180411. doi: 10.1371/journal.pone.0180411 (PMC5570493; doi:10.1371/journal.pone.0180411)
Supplement: S1 Table — (PDF) [file pone.0180411.s004.pdf]

Appendix Table 1: Predictors of the Respondent's Level of Health and Wealth Altruism Including Additional Respondent Characteristics (Ordered Probit Estimated Coefficients)

	Survey 1: Amount Given in Dictator Game							Survey 2: MRS Implied by Dichotomous Choices						
	Health			Wealth				Health			Wealth			
	Coef.	(s.e.)	Sig.	Coef.	(s.e.)	Sig.	Sig. of Diff.	Coef.	(s.e.)	Sig.	Coef.	(s.e.)	Sig.	Sig. of Diff.
**Question is about Wealth**	Baseline			-1.50	(0.67)	**		Baseline			-0.16	0.71		
**Characteristics of the Other Person**														
Social Proximity (relative to Stranger: Rest of World):														
Immediate Family Member	0.61	(0.08)	***	0.63	(0.07)	***		0.65	(0.06)	***	0.60	(0.06)	***	
Extended Family Member	0.53	(0.06)	***	0.46	(0.06)	***		0.52	(0.06)	***	0.41	(0.05)	***	
Close Friend	0.58	(0.08)	***	0.65	(0.08)	***		0.31	(0.08)	***	0.53	(0.07)	***	**
Co-Worker	0.21	(0.09)	**	0.41	(0.09)	***		0.01	(0.08)		0.21	(0.08)	**	*
Acquaintance	0.29	(0.06)	***	0.31	(0.06)	***		0.26	(0.06)	***	0.16	(0.06)	***	
Stranger: U.S.	0.09	(0.04)	**	0.13	(0.04)	***		0.15	(0.04)	***	0.08	(0.04)	**	
Age of other person (relative to age 80 and older):														
0-4	0.20	(0.09)	**	-0.17	(0.11)		**	0.37	(0.09)	***	-0.21	(0.07)	***	***
5-9	0.16	(0.09)	*	-0.06	(0.08)		*	0.42	(0.10)	***	-0.15	(0.08)	**	***
10-17	0.07	(0.10)		0.11	(0.08)			0.23	(0.09)	***	-0.19	(0.08)	**	***
18-29	-0.04	(0.09)		0.04	(0.06)			0.16	(0.08)	**	0.05	(0.06)		
30-39	0.13	(0.09)		0.04	(0.09)			0.12	(0.07)	*	-0.06	(0.06)		**
40-49	0.05	(0.08)		0.07	(0.07)			0.14	(0.07)	*	0.01	(0.06)		
50-59	0.10	(0.08)		0.03	(0.06)			0.12	(0.07)		-0.03	(0.07)		
60-69	0.20	(0.08)	***	0.09	(0.07)			0.18	(0.08)	**	0.04	(0.06)		
70-79	0.11	(0.08)		0.05	(0.07)			0.16	(0.08)	**	0.03	(0.06)		
**Characteristics of the Respondent**														
Female	-0.02	(0.10)		0.06	(0.10)			0.16	(0.06)	***	0.06	(0.06)		*
Age	-0.057	(0.020)	***	-0.016	(0.020)		*	-0.021	(0.012)	*	0.004	(0.012)		**
Age^2	0.00038	(0.00021)	*	0.00007	(0.00021)			0.00019	(0.00012)		-0.00004	(0.00013)		*
Relative to those who reside in birth state:														
Live in Different State from Birth, Within 500 Miles	0.01	(0.13)		0.11	(0.13)			-0.02	(0.07)		-0.02	(0.07)		
Live in Different State from Birth, 500-999 Miles	-0.21	(0.19)		-0.41	(0.17)	**		-0.07	(0.11)		-0.06	(0.10)		
Live in Different State from Birth, 1000+ Miles	0.00	(0.15)		-0.05	(0.14)			-0.01	(0.11)		0.05	(0.10)		
Born Outside U.S.	0.27	(0.22)		0.10	(0.19)			-0.20	(0.10)	**	-0.05	(0.10)		
**Framing Effects**														
Asked health altruism questions first	0.15	(0.15)		0.02	(0.12)			0.32	(0.07)	***	0.16	(0.07)	**	**
Other person placed on left (before the respondent)	0.00	(0.15)		-0.05	(0.12)			-0.10	(0.09)		-0.14	(0.08)	*	
Interaction of above framing issues	0.33	(0.20)	*	0.21	(0.19)			-0.01	(0.11)		0.02	(0.11)		
Relative to initially asked about an equal allocation:														
Initially asked about a less generous allocation								0.07	(0.04)	*	0.07	(0.04)	**	
Initially asked about a more generous allocation								-0.09	(0.04)	**	-0.01	(0.04)		

Appendix Table 1 is continued on the next page

	Survey 1: Amount Given in Dictator Game							Survey 2: MRS Implied by Dichotomous Choices						
	**Health**			**Wealth**				**Health**			**Wealth**			
	Coef.	(s.e.)	Sig.	Coef.	(s.e.)	Sig.	Sig. of Diff.	Coef.	(s.e.)	Sig.	Coef.	(s.e.)	Sig.	Sig. of Diff.
**Additional Control Variables**														
Household Income (Relative to Less than $15,000):														
$15,000 to $19,999	0.35	(0.35)		0.33	(0.26)			-0.04	(0.18)		-0.04	(0.18)		
$20,000 to $24,999	0.11	(0.36)		0.09	(0.27)			-0.21	(0.17)		0.12	(0.16)		**
$25,000 to $29,999	-0.01	(0.28)		-0.02	(0.25)			0.08	(0.16)		0.16	(0.15)		
$30,000 to $34,999	-0.28	(0.30)		0.12	(0.27)			0.26	(0.15)	*	0.13	(0.15)		
$35,000 to $39,999	0.18	(0.29)		0.45	(0.23)	**		-0.23	(0.15)		-0.25	(0.16)		
$40,000 to $49,999	0.04	(0.27)		-0.01	(0.23)			-0.09	(0.14)		0.04	(0.14)		
$50,000 to $59,999	-0.06	(0.26)		0.21	(0.24)			-0.09	(0.14)		-0.15	(0.13)		
$60,000 to $74,999	-0.35	(0.24)		0.03	(0.22)		*	-0.07	(0.13)		-0.10	(0.13)		
$75,000 to $84,999	0.41	(0.28)		0.39	(0.30)			0.02	(0.14)		-0.18	(0.15)		
$85,000 to $99,999	-0.34	(0.24)		-0.02	(0.25)			-0.13	(0.16)		-0.12	(0.14)		
$100,000 to $124,999	-0.28	(0.23)		-0.05	(0.23)			-0.01	(0.13)		-0.08	(0.14)		
$125,000 to $149,999	-0.32	(0.23)		-0.28	(0.24)			-0.35	(0.17)	**	-0.24	(0.16)		
$150,000 to $174,999	-0.27	(0.44)		-0.44	(0.28)			-0.39	(0.23)	*	-0.38	(0.20)	*	
$175,000 or more	-0.20	(0.27)		0.25	(0.30)			-0.36	(0.16)	**	-0.06	(0.16)		*
Education (Relative to High School Dropout):														
High School Graduate	-0.55	(0.20)	***	-0.38	(0.18)	**		0.23	(0.11)	**	0.04	(0.12)		*
Some College, No Degree	-0.34	(0.20)	*	-0.25	(0.20)			0.28	(0.12)	**	0.19	(0.13)		
Associate's Degree	-0.53	(0.25)	**	-0.40	(0.24)	*		0.31	(0.13)	**	0.17	(0.14)		
Bachelor's Degree	-0.84	(0.22)	***	-0.69	(0.21)	***		0.21	(0.13)		0.02	(0.14)		
Master's Degree	-0.53	(0.26)	**	-0.20	(0.25)			0.33	(0.14)	**	0.30	(0.15)	**	
Professional Degree or Doctorate	-0.74	(0.33)	**	-0.27	(0.26)			0.47	(0.21)	**	-0.03	(0.20)		***
Number of warm-up questions answered incorrectly (Relative to 0):														
1	-0.10	(0.13)		-0.10	(0.11)			-0.06	(0.07)		0.08	(0.07)		**
2	-0.07	(0.16)		0.21	(0.16)		*	0.25	(0.12)	**	0.18	(0.12)		
3	0.17	(0.21)		0.64	(0.27)	**	**	0.29	(0.14)	**	0.41	(0.15)	***	
4 or 5	0.71	(0.33)	**	1.04	(0.28)	***		0.33	(0.36)		0.43	(0.25)	*	
Self-Reported Understanding of Probability (Relative to Refused):														
I do not understand probability at all	-0.18	(0.37)		-0.14	(0.43)			0.42	(0.33)		0.52	(0.47)		
I have a poor understanding of probability	-0.33	(0.35)		-1.00	(0.38)	***	*	0.26	(0.33)		0.19	(0.46)		
I have a fair understanding of probability	-0.35	(0.30)		-0.78	(0.36)	**		0.20	(0.32)		0.02	(0.45)		
I have a good understanding of probability	-0.25	(0.31)		-0.76	(0.34)	**	*	0.29	(0.32)		0.01	(0.45)		
I have an excellent understanding of probability	-0.18	(0.33)		-0.88	(0.35)	**	**	0.24	(0.33)		-0.05	(0.45)		

Appendix Table 1 is continued on the next page

	Survey 1: Amount Given in Dictator Game							Survey 2: MRS Implied by Dichotomous Choices						
	Health			Wealth				Health			Wealth			
	Coef.	(s.e.)	Sig.	Coef.	(s.e.)	Sig.	Sig. of Diff.	Coef.	(s.e.)	Sig.	Coef.	(s.e.)	Sig.	Sig. of Diff.
**Additional Control Variables (Continued)**														
Relative to White, Non-Hispanic:														
Black, Non-Hispanic	-0.13	(0.18)		-0.05	(0.16)			-0.07	(0.09)		0.18	(0.09)	**	***
Other, Non-Hispanic	-0.29	(0.34)		-0.14	(0.34)			0.07	(0.15)		0.14	(0.15)		
Hispanic	-0.04	(0.16)		0.06	(0.17)			0.10	(0.10)		0.35	(0.09)	***	***
2+ Races, Non-Hispanic	0.15	(0.21)		0.38	(0.18)	**		-0.39	(0.20)	**	-0.06	(0.15)		*
Household Head	0.33	(0.13)	**	0.20	(0.13)			0.05	(0.09)		-0.02	(0.09)		
Household Size (Relative to Living Alone):														
2	0.07	(0.18)		0.02	(0.16)			0.19	(0.09)	**	0.09	(0.09)		
3	0.13	(0.21)		-0.05	(0.20)			0.11	(0.11)		0.07	(0.11)		
4	0.56	(0.24)	**	0.06	(0.22)		**	0.29	(0.14)	**	0.10	(0.13)		
5	0.59	(0.33)	*	-0.18	(0.28)		**	0.32	(0.19)	*	0.06	(0.19)		
6 or more	0.93	(0.37)	**	-0.36	(0.33)		***	0.37	(0.20)	*	-0.10	(0.19)		**
House Type (Relative to One-Family Detached House):														
One-Family Attached House	0.10	(0.22)		-0.02	(0.22)			-0.06	(0.10)		-0.05	(0.10)		
Building with 2+ Apartments	0.46	(0.16)	***	0.18	(0.14)		*	-0.05	(0.08)		-0.03	(0.08)		
Mobile Home, Boat, RV, Van, etc.	0.34	(0.28)		0.51	(0.24)	**		-0.18	(0.16)		0.14	(0.14)		**
Marital Status (Relative to Married):														
Widowed	0.07	(0.30)		0.10	(0.26)			-0.15	(0.18)		0.04	(0.18)		
Divorced	-0.30	(0.16)	*	-0.39	(0.16)	**		0.00	(0.12)		-0.32	(0.12)	***	***
Separated	-0.42	(0.30)		0.47	(0.55)		*	0.20	(0.20)		-0.04	(0.19)		
Never Married	-0.54	(0.19)	***	-0.24	(0.17)		*	0.12	(0.10)		0.14	(0.09)		
Living with Partner	-0.46	(0.20)	**	-0.25	(0.18)			0.02	(0.11)		-0.16	(0.12)		
Lives in Metropolitan Statistical Area	-0.04	(0.13)		-0.13	(0.13)			-0.10	(0.09)		-0.04	(0.08)		
Census Region (Relative to Northeast):														
Midwest	0.00	(0.16)		0.11	(0.17)			0.13	(0.09)		0.06	(0.09)		
South	-0.10	(0.15)		-0.05	(0.15)			0.16	(0.09)	*	-0.08	(0.08)		***
West	-0.19	(0.18)		-0.09	(0.17)			0.25	(0.09)	***	0.03	(0.09)		**
Current Employment Status (Relative to Paid Employee):														
Self-employed	-0.06	(0.19)		-0.02	(0.17)			0.29	(0.14)	**	0.09	(0.12)		*
Not working (Looking for Work or Temp. Layoff)	-0.14	(0.16)		-0.09	(0.17)			0.23	(0.11)	**	0.11	(0.11)		
Not Working (Retired)	0.00	(0.17)		0.14	(0.18)			-0.14	(0.11)		0.02	(0.12)		
Not Working (Disabled)	-0.31	(0.20)		0.34	(0.22)		***	0.21	(0.12)	*	0.23	(0.11)	**	
Not Working (Other)	-0.23	(0.19)		-0.02	(0.17)			0.22	(0.11)	**	0.09	(0.11)		

Appendix Table 1 is continued on the next page

	Survey 1: Amount Given in Dictator Game							Survey 2: MRS Implied by Dichotomous Choices						
	Health			Wealth				Health			Wealth			
	Coef.	(s.e.)	Sig.	Coef.	(s.e.)	Sig.	Sig. of Diff.	Coef.	(s.e.)	Sig.	Coef.	(s.e.)	Sig.	Sig. of Diff.
**Additional Control Variables (Continued)**														
Number of Household Members Under 18 (Relative to 0):														
1	-0.34	(0.17)	*	0.10	(0.14)		**	-0.06	(0.10)		0.01	(0.10)		
2	-0.47	(0.28)	*	0.23	(0.25)		**	-0.02	(0.13)		0.06	(0.13)		
3	-0.76	(0.34)	**	0.13	(0.27)		***	-0.01	(0.23)		0.24	(0.20)		
4 or more	-0.89	(0.45)	**	0.37	(0.43)		***	-0.69	(0.36)	*	-0.24	(0.28)		

**P-Value for Test of Joint Significance of:**	Survey 1 Health		Survey 1 Wealth		Survey 1 Combined		Survey 2 Health		Survey 2 Wealth		Survey 2 Combined	
Relationship	0.0%	***	0.0%	***	0.0%	***	0.0%	***	0.0%	***	0.0%	***
Age of other person	2.0%	**	29.3%		0.7%	***	0.0%	***	0.0%	***	0.0%	***
Age and Age^2	0.0%	***	18.2%		0.0%	***	20.0%		95.0%		20.0%	
Migration from birth location	46.8%		6.8%	*	14.5%		36.7%		86.5%		66.8%	
Framing Effects	0.2%	***	44.9%		1.9%	**	0.0%	***	0.1%	***	0.0%	***

	Survey 1	Survey 2
**Number of Observations in the Regression**	9,829	10,834
**Number of Respondents in the Regression**	496	1,125

Note: Robust standard errors are used, clustered by survey respondent. Two-tailed statistical significance at the 1%, 5%, and 10% levels are indicated, respectively, by ***, **, and *.
